# Orthogonal photoswitching in a multifunctional molecular system

**DOI:** 10.1038/ncomms12054

**Published:** 2016-07-12

**Authors:** Michael M. Lerch, Mickel J. Hansen, Willem A. Velema, Wiktor Szymanski, Ben L. Feringa

**Affiliations:** 1 Centre for Systems Chemistry, Stratingh Institute for Chemistry, University of Groningen, Nijenborgh 4, 9747 AG Groningen, The Netherlands; 2 Department of Radiology, University of Groningen, University Medical Center Groningen, Hanzeplein 1, 9713 GZ Groningen, The Netherlands

## Abstract

The wavelength-selective, reversible photocontrol over various molecular processes in parallel remains an unsolved challenge. Overlapping ultraviolet-visible spectra of frequently employed photoswitches have prevented the development of orthogonally responsive systems, analogous to those that rely on wavelength-selective cleavage of photo-removable protecting groups. Here we report the orthogonal and reversible control of two distinct types of photoswitches in one solution, that is, a donor–acceptor Stenhouse adduct (DASA) and an azobenzene. The control is achieved by using three different wavelengths of irradiation and a thermal relaxation process. The reported combination tolerates a broad variety of differently substituted photoswitches. The presented system is also extended to an intramolecular combination of photoresponsive units. A model application for an intramolecular combination of switches is presented, in which the DASA component acts as a phase-transfer tag, while the azobenzene moiety independently controls the binding to α-cyclodextrin.

## Abstract (web summary)

While molecular photoswitches have proven useful in many fields, selective and reversible control over multiple switches is an unsolved challenge. Here, the authors report on a system consisting of two classes of photoswitches that can be addressed orthogonally and demonstrate the applicability in phase-transfer control.

## Graphical abstract

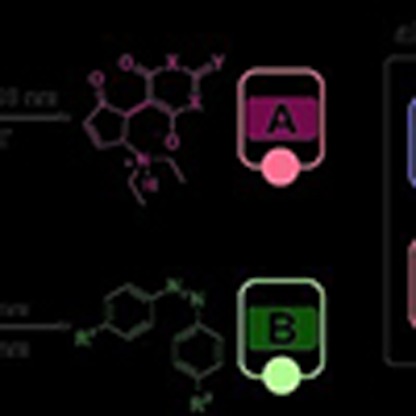

Light as an external stimulus has been extensively used in chemistry, biology and material sciences to control processes and properties in a non-invasive manner with high spatiotemporal precision^1^,^2^,^3^,^4^,^5^,^6^,^7^. However, as systems grow more complex, this control often needs to be extended to processes that act simultaneously, using multiple sources of light at different wavelenghts^8^ in an orthogonal manner. When Bochet and co-workers^9^,^10^ reported the proof-of-principle for the irreversible wavelength-selective removal of photolabile protecting groups (PPGs; Fig. 1a) in 2000, it paved the way for numerous impressive applications^8^. These include functionalized surfaces^11^, hydrogels^12^ and lithography^13^, control of nucleic acids^14^, gene expression^15^,^16^, regulation of enzyme activity^17^,^18^ and interfering with neuronal processes^19^. Despite the successful use of this concept, light-mediated uncaging is an inherently irreversible process^6^, which limits future applications towards responsive systems both in material and life sciences.

On the other hand, the field of molecular photoswitches exploits the reversibility of the photoswitching process^2^,^3^,^20^,^21^,^22^,^23^,^24^. However, the concept of addressing photoactive molecules in parallel with light of different wavelengths has not been translated from wavelength-selective uncaging of PPGs to independent photoswitching (Fig. 1b). Such orthogonal control has great potential for employment in various fields, as many of the above-mentioned applications used for wavelength-selective uncaging would benefit from the reversibility of activation. Furthermore, reversible orthogonal control would enable independent modulation of different material properties and investigation of complex networks of signalling pathways in biological systems. The wavelength selectivity (orthogonality) of the photoswitching is essential and the lack thereof currently represents a major limiting factor for successful applications (N.B.: throughout this article we refer to orthogonality as a characteristic of a system, where each component of the system can be controlled independently and irrespective of the order of addressing)^25^.

Although the idea of combining two different photoswitches in an intermolecular manner and addressing them independently may seem simple, its execution remains a major challenge^26^. Wavelength-selective control was so far confined to irreversible processes likely because of the fact that many of the most abundantly used photoswitches, such as azobenzenes, diarylethenes and spiropyrans, have overlapping absorption bands in the ultraviolet-visible region^2^,^8^. Thus, it is difficult to find spectral regions with large differences in photoswitching efficiency (defined, at a given wavelength, as a product of extinction coefficient, *ɛ* and quantum yield, *φ*). Moreover, the possibility of undesirable energy transfer between the chromophores^27^ can lead to a major loss of selectivity. Herein, we present an orthogonal, intermolecular combination of two classes of photoswitches and their intramolecular combination as a first-generation orthogonally addressable, dual functional molecular system. The reported intermolecular combination of photoswitches is based on a donor–acceptor Stenhouse adduct (DASA, Fig. 1c) and an azobenzene (Fig. 1d). Both photoswitches can be independently controlled by irradiation with light of three different wavelengths and by taking advantage of a thermal isomerization process. The modular and experimentally robust combination allows for functionalization of each constituent. A model application for an intramolecular combination of switches is presented, in which the DASA component acts as a phase-transfer tag, while the azobenzene moiety independently controls the binding to α-cyclodextrin (α-CD).

## Results

### Selecting a compatible photoswitch pair

**Figure 1:**
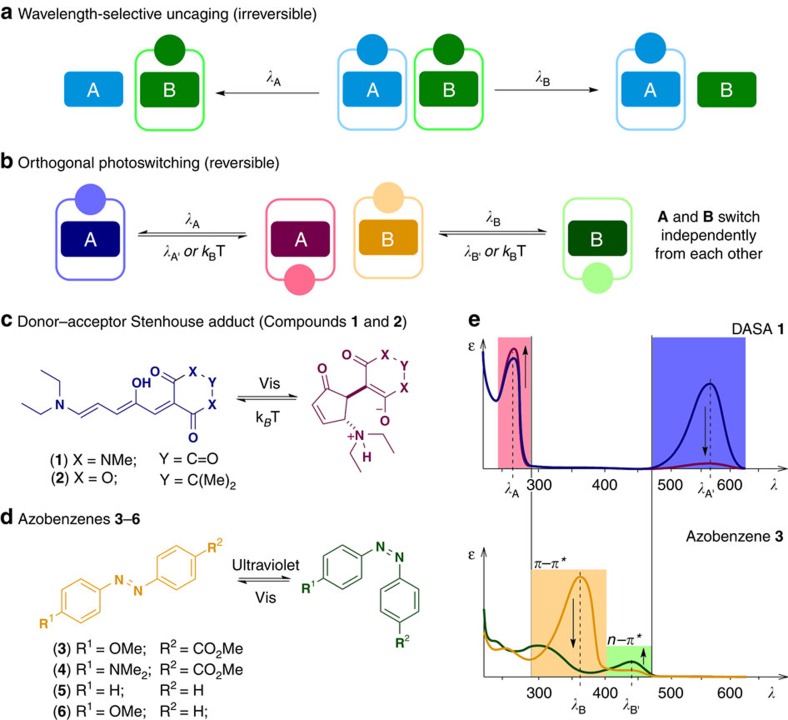
Orthogonal reversible photoswitching. Comparison of concepts described in this manuscript: wavelength-selective uncaging of photolabile protecting groups (**a**) and orthogonal photoswitching (**b**). Molecular structures and switching of DASAs (DASA **1** and **2** (**c**)) and azobenzenes (**3**–**6** (**d**)) with a conceptual representation of the ultraviolet-visible spectra of compounds **1** and **3** (**e**).

Defining a photochromically compatible pair of photoswitches would constitute the first example of wavelength-selective/orthogonal control of multiple photoswitches in one solution. For this reason, we aimed to find compatible classes of photoswitches (Fig. 1), especially those that would exhibit a high complementarity in the 300–800 nm spectral region where photoswitches normally operate (Fig. 1e). We chose azobenzene photoswitches as one class: they have been successfully applied in molecular photocontrol^20^,^21^,^22^,^23^,^24^,^28^,^29^,^30^,^31^, are easily accessible and allow for custom modification, functionalization and spectral fine-tuning. Azobenzenes have their main absorption bands located between 300 and 500 nm (*π*–π* and *n*–π* transitions; Fig. 1d,e and Table 1)^20^,^32^. We envisioned that the T-type^2^ DASAs (DASA **1** and DASA **2**, Fig. 1c) could be used as the complementary class^33^,^34^ since they show very little absorption between 300 and 500 nm (Fig. 1c,e and Table 1)^35^,^36^,^37^. Owing to the fact that the reverse switching of DASAs is thermally driven^33^,^34^,^36^,^37^, three different wavelengths would be sufficient for wavelength-selective control. Importantly, for the design of orthogonal systems, one can exploit the tunability of thermal half-lives of the thermodynamically unstable states of the photoswitches in use. By changing the substitution pattern of azobenzenes, both very long and very short thermal half-lives have been attained. Increased thermal stability could be achieved, for example, through *ortho*-substitution^32^,^38^,^39^,^40^,^41^. On the other hand, compounds with shortened thermal half-lives can be obtained by tuning of the electron density, through incorporation of both electron-donating groups (for example, -NMe_2_ and -OMe), electron-withdrawing groups and combinations of both (push–pull systems)^42^. For the DASAs a clear relation between half-life and structure has not been determined yet^33^,^34^. However, it has been shown that changing the acceptor can influence the half-life to some extent^33^,^34^. Methylbarbituric acid-based cyclized DASAs (Fig. 1c, X=NMe; Y=C=O) are generally less thermally stable in comparison with the ones based on Meldrum's acid (Fig. 1c, X=O; Y=C(Me)_2_). Finally, tuning half-lives of switches is often relatively independent of structural features required for function. This leaves ample space to tune the thermal relaxation rate of switches to magnitudes needed for different applications^20^,^21^,^22^,^23^,^24^,^28^,^29^,^30^,^31^,^43^.

### Intermolecular combination of photoswitches

Initially, we studied push–pull azobenzene **3** in combination with DASA **1** (Fig. 1 and Table 1). The 4′-methoxy group in azobenzenes increases photostationary states^44^ (PSS, defined as the percentage of the thermodynamically unstable isomer that can be produced upon irradiation at a given wavelength), while the ester moiety represents a handle for convenient functionalization. The absorption spectrum of the mixture of **1** and **3** is, to a large degree, a linear combination of the spectra of the separate photoswitches, indicating a low level of intermolecular energy transfer (Supplementary Fig. 39)^26^.

**Figure 2:**
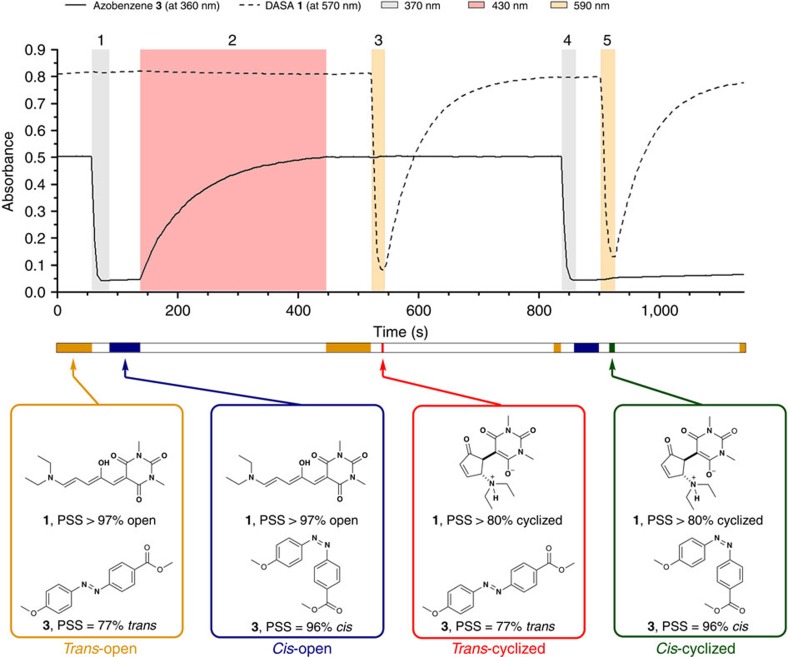
Orthogonal control of a mixture of photoswitches. Proof-of-concept for the reversible and orthogonal photocontrol in a mixture of DASA **1** (—) and azobenzene **3** (- - -). The orthogonal photoswitching of both compounds in one solution (**1**: ∼4 μM; **3**: ∼20 μM; toluene, room temperature) was monitored at characteristic wavelengths for each photoswitch (*λ*=360 nm for **3** and *λ*=570 nm for **1**). Steps 1–5 indicate distinct irradiation experiments. Coloured areas indicate different wavelengths of irradiation. The coloured bar below the figure indicates the different states of the photoswitches. The structure of photoswitches and their corresponding photostationary states (determined by ^1^H-NMR and ultraviolet-visible spectroscopy) are given for the different states during photoswitching.

The combination of photoswitches **1** and **3** showed a remarkably high level of wavelength selectivity, apparent in ultraviolet-visible spectra and reversible photochromism plots (Fig. 2). Irradiation of azobenzene **3** led to a selective, nearly quantitative *trans–cis* isomerization (*λ*=370 nm, irradiation 1, Fig. 2), which proceeds with a quantum yield of *ϕ*=0.15. The relatively long half-life of the *cis*-isomer of azobenzene **3** (*t*_1/2_>8 h; Table 2) makes it thermally stable on the timescale of the experiment (<1 h). Irradiation of the *cis*-**3** isomer (*λ*=430 nm, irradiation 2, Fig. 2) allowed selective *cis–trans* isomerization without affecting photoswitch **1**. DASA **1** could be selectively cyclized by longer wavelength irradiation (*λ*=590 nm, irradiation 3, Fig. 2), without affecting the azobenzene, after which it showed a short thermal half-life for cycloreversion (37 s at room temperature; Table 2). DASA **1** can also be switched selectively (irradiation 5, Fig. 2) without affecting the azobenzene **3** after it has been switched to the *cis*-isomer (irradiation 4, Fig. 2). By using a broad-spectrum visible light source (white light, Supplementary Table 1), both switches could be addressed at the same time (Supplementary Fig. 41) and different combinations of PSS could be attained by adjusting the irradiation intensity and/or duration (Supplementary Fig. 41). Moreover, this intermolecular combination operates successfully throughout a large concentration range (Supplementary Figs 51–53).

In summary, in a mixture of photoswitches **1** and **3** (Fig. 2), azobenzene **3** could be selectively switched from *trans* to *cis* (irradiation 1) and back from *cis* to *trans* (irradiation 2). The same applies for DASA photoswitch **1**, which could be selectively cyclized (irradiation 3) and then relaxed back to its thermally stable state. It is important to note that full chromatic orthogonality was observed because both states of **1**, as well as the thermal relaxation process, were not affected by light pulses that address **3** (irradiations 1, 2 and 4). Similarly, both states of **3** were not affected by light pulses addressing **1** (irradiations 3 and 5).

### Structural scope of photoswitches

**Figure 3:**
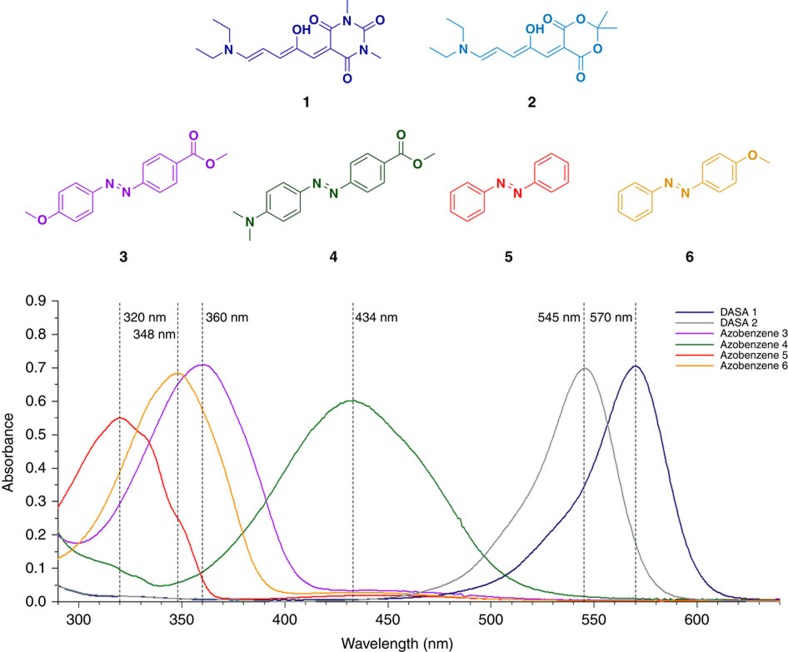
Structural scope of photoswitches. Structural scope of DASAs (**1** and **2**) and azobenzenes (**3**–**6**). Overlay of the ultraviolet-visible absorption spectra of compounds **1**–**6** (compounds **1** and **2**: ∼4 μM; compounds **3**–**6**: ∼20 μM; toluene; room temperature) with their corresponding absorption maxima.

Subsequently, we investigated whether other azobenzenes could be used in a two-switch system with compound **1**. Towards that end, azobenzenes **4**–**6** (Fig. 3 and Table 2) were selected. These photochromes are not only structurally diverse, but they also exhibit very different functional characteristics: the *λ*_max_ of **4** is bathochromically shifted^20^,^38^,^39^,^40^,^41^,^42^, whereas *λ*_max_ of **5** and **6** are hypsochromically shifted (Fig. 3 and Table 2) as compared with compound **3**. Moreover, they differ markedly in the half-lives of their thermally unstable *cis*-isomers as compared with **3**, with a shortened half-life for **4** 20,32,38,39,40,41,42, comparable half-life for **6** and a longer half-life for **5** (ref. 20; Table 2; Supplementary Figs 16, 20 and 24). All combinations of DASA **1** with azobenzenes **3**, **4, 5** or **6** showed chromatic orthogonality, thus showing the tolerance of this photoswitch combination towards structural modification of the azobenzene core with functionally different behaviour (Supplementary Figs 43–48).

We then investigated possibilities for structural diversity of DASAs that can be incorporated in an orthogonal switching system with azobenzenes. In principle, changing the acceptor part of this photochromic compound affects the absorption maximum and thermal half-life, while the donor moiety has little influence on *λ*_max_, affecting only its solubility^33^,^34^. DASA **2** (Fig. 3), with a Meldrum's acid-derived acceptor moiety, shows a hypsochromically shifted absorption maximum (Fig. 3 and Table 2) and its cyclized form exhibits a longer half-life (∼130 s; Supplementary Fig. 8), when compared with that of DASA **1** (refs 33, 34). We selected azobenzene **6** (Fig. 3) to study the orthogonality of photoswitching in solution since it is compatible with DASA **2** because of its hypsochromically shifted *π*–π* absorption band (Fig. 3, Table 2 and Supplementary Fig. 22). Gratifyingly, both switches **2** and **6** can also be selectively addressed in parallel with light of different wavelengths (Supplementary Figs 49 and 50). The flexibility with regard to the DASA photoswitch highlights the robustness and modularity of the photoswitch combination and bodes well for versatile use in different applications.

### Orthogonal photoswitching in an intramolecular system

Apparently, loss of selectivity through energy transfer did not pose a problem for the intermolecular system described thus far, even at high concentrations up to ∼80 μM (**1**) and ∼400 μM (**3**; Supplementary Figs 51–53). Therefore, the reversible photocontrol in the intermolecular case is purely dependent on the spectral properties of the two photoswitches involved. For an intramolecular combination, on the other hand, linker design, geometry and distance of the photochromes become more important^26^. While intramolecular energy transfer between chromophores can drastically reduce the switching selectivity^8^,^26^,^27^, it can be partially controlled by adjusting the lifetime of the photo-excited state and/or by changing the design of the linker^8^,^26^,^27^. Furthermore, properties that arise from energy transfer and the interplay of the combination of two photoswitches can sometimes be desired. This is the case, for example, in molecular logics^26^,^45^,^46^,^47^,^48^, where such hybrid multiswitches (or multiphotochromes) allow (sequential) access to multiple different states^45^,^46^, usually in combination with external stimuli other than light^49^,^50^,^51^,^52^,^53^,^54^,^55^,^56^.

**Figure 4:**
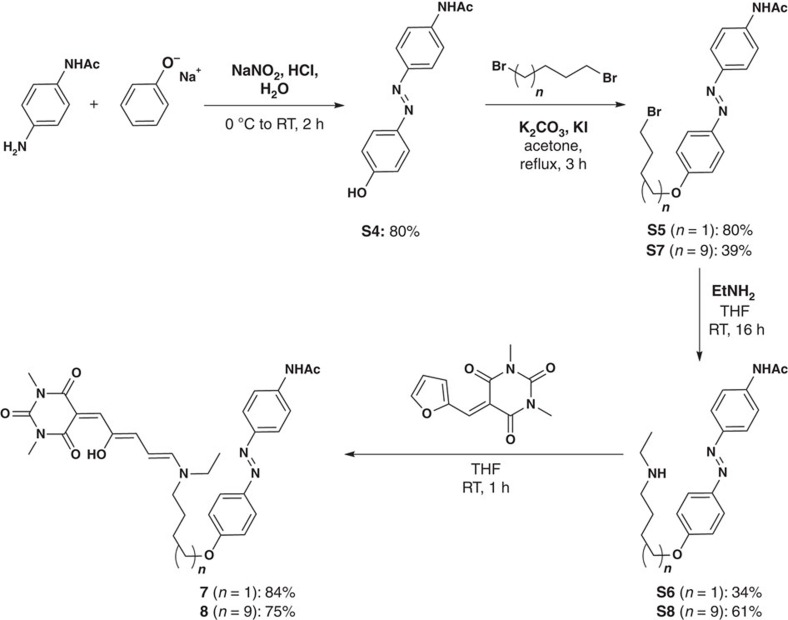
Synthesis of compounds **7** and **8**. Compounds **7** and **8** were used to probe the chromatic selectivity in intramolecular photoswitching.

**Figure 5:**
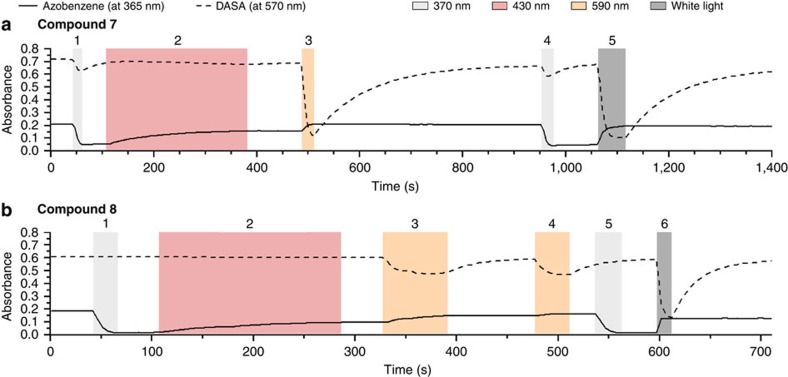
Selectivity in intramolecular photoswitching. Reversible photochromism of an intramolecular photoswitch combination of azobenzene and DASA with different linker lengths. Compound **7** (**a**) and compound **8** (**b**; ∼4 μM; toluene, room temperature). Steps 1–6 indicate distinct irradiation experiments.

To probe whether the level of selectivity of the intermolecular combination could be retained in the intramolecular system, or whether new properties could be observed, we combined an azobenzene and a DASA photoswitch into a single molecule. Towards that end, compounds **7** and **8**, which differ only in the length of the linker, were synthesized (Fig. 4) and the selectivity of their photoresponsiveness was evaluated (Fig. 5).

Importantly, the selective switching of the azobenzene moiety is retained. However, irradiation with *λ*=590 nm not only switched the DASA moiety but also affected, to some extent, the azobenzene part in both **7** and **8** (Fig. 5). Generally, improved selectivities were observed with the elongated linker unit (from -(CH_2_)_4_- to -(CH_2_)_12_-). In compound **7** (shorter linker), irradiation at *λ*=370 nm (Fig. 5a) does partially affect the DASA moiety, which is not observed for compound **8** (longer linker). Interestingly, compound **8** shows a slight decrease in addressability of exclusively the DASA photoswitch with *λ*=590 nm, but not with white light (Fig. 5b). No such effect is observed for the compound with shorter linker (compound **7**). As a control experiment, the intermolecular switching of the corresponding azobenzenes in combination with the DASA photoswitch **1** was studied and orthogonality was observed (Supplementary Figs 54–58).

### Modulation of phase transfer and supramolecular interactions

**Figure 6:**
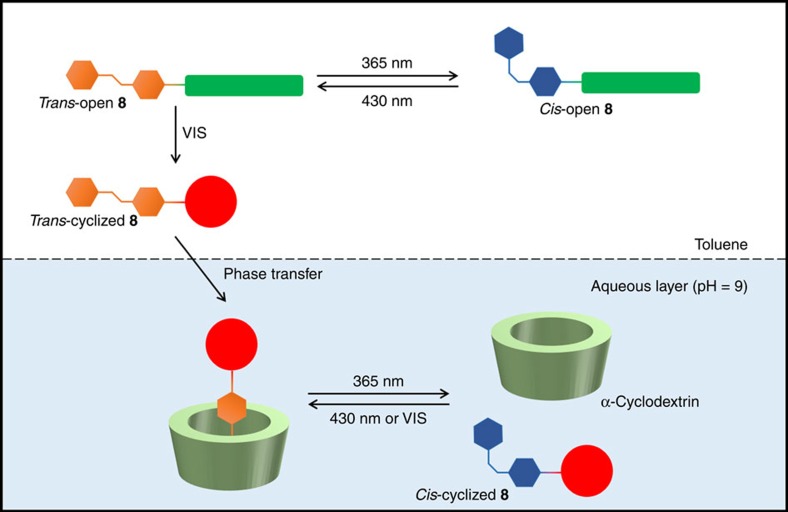
Design principle of switchable phase transfer and binding. Schematic outline of the application of light-controlled guest **8** with light-controlled phase-transfer properties. Compound **8** (*trans*-open) is soluble in toluene. Irradiation with *λ*=365 nm (*trans*–*cis* isomerization) and *λ*=430 nm (*cis*–*trans* isomerization) controls the state of the azobenzene. Cyclization of the DASA moiety with visible light results in phase transfer of compound **8**. In the aqueous layer (pH ⩾9), the azobenzene binds the water-soluble α-CD in its *trans*-state, but not in the *cis*-state.

We proceeded to apply this intramolecular system for controlling the location and the function of the molecule. Previously reported extraction experiments of DASA molecules^33^,^34^ established the potential of DASAs as a phase-transfer tag^34^,^37^. Reversible photoswitching of DASAs is only observed in aromatic solvents. Polar protic solvents (as, for example, methanol or water) stabilize DASA's zwitterionic cyclized state, whereas halogenated solvents favour the elongated triene structure. Photoswitching results in formation of the hydrophilic zwitterionic cyclized form and phase transfer to the aqueous layer^34^. We envisioned exploiting this behaviour to transport photoswitchable cargo that is, an azobenzene. On an independent level of photocontrol, the isomerization of the azobenzene could be used to manipulate a certain function, for example, the well-known host–guest binding to CDs (especially α-CD)^57^,^58^,^59^,^60^. Therefore, we set off to evaluate a model system with dual functionality (Fig. 6) in which the DASA moiety of **8** controls the phase where the molecule resides (location), whereas the azobenzene moiety controls the supramolecular binding to α-CD (function). In toluene, the azobenzene moiety of compound **8** could be switched selectively between *trans*- and *cis*-forms (Fig. 5b). Upon cyclization of the DASA moiety, phase transfer of compound **8** to the aqueous layer is expected. In the aqueous layer, the photocontrol of the azobenzene moiety would allow control over binding to the water-soluble α-CD.

**Figure 7:**
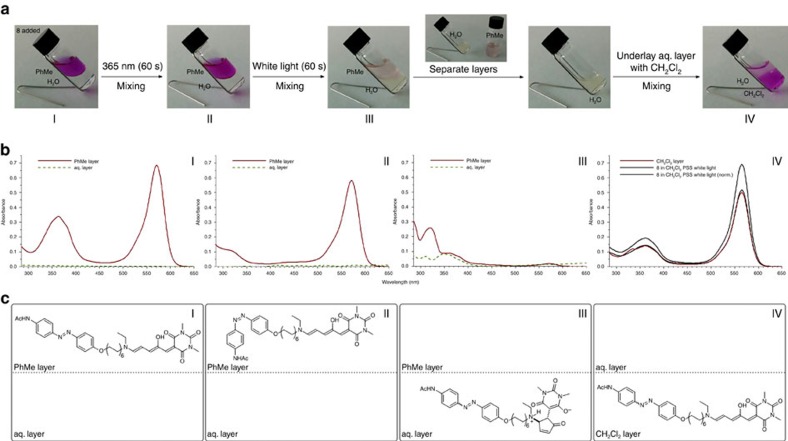
Demonstration of light-controlled phase transfer. Dynamic phase transfer of compound **8** in a toluene/aqueous NaHCO_3_ (pH=9) mixture. The independent switching and phase-transfer events can be monitored by phase-colour (**a**) and ultraviolet-visible spectroscopy (**b**). *Stage I*: initial conditions, compound **8** resides in the organic phase (purple colour). *Stage II*: the azobenzene part of compound **8** was switched with irradiation at *λ*=365 nm. The DASA part remains untouched. *Stage III*: irradiation with white light results in the cyclization of the DASA moiety, decolouration and phase transfer of compound **8**. *Stage IV*: separating the layers and back-extraction of the aqueous layer with dichloromethane results in back-transfer of compound **8** to the organic phase, as can be observed by the recolouration of this phase (purple colour). The presence of compounds in the different phases is schematically depicted (**c**).

Towards that end, we established the phase-transfer behaviour of DASA in the intramolecular system **8**. Inspired by traditional log*P*^I^_(oct/wat)_ measurements^61^, a functional phase-transfer system was designed to operate at pH 9 (using a saturated aqueous NaHCO_3_ solution to adjust the pH Fig. 7). The extraction experiment described in Fig. 7 establishes the photoswitching and light-controlled phase-transfer behaviour of compound **8**. Compound **8** is dissolved in toluene and the organic layer is underlayed with water (pH 9). Upon irradiation of the biphasic mixture (stage I, Fig. 7) with *λ*=365 nm, the azobenzene part of **8** is selectively switched, as can be observed by ultraviolet-visible spectroscopy (stage II, Fig. 7). Subsequent irradiation with white light results in a successful phase transfer of **8** to the aqueous layer (stage III, Fig. 7). Separation of the biphasic mixture and addition of dichloromethane to the aqueous phase results in back-extraction and recolouration of the organic solution (stage IV, Fig. 7), taking advantage of the short half-life of the DASA **1**-type moiety. Importantly, the geometry of the azobenzene moiety can be controlled without affecting the DASA moiety in the organic phase (Fig. 7). Isolation of phase-transferred compound **8** and photoswitching in water (pH ⩾9, K_2_CO_3_) established reversibility with negligible fatigue of the azobenzene moiety in the basic aqueous phase (Supplementary Figs 61, 62 and 69).

Having established the light-induced phase transfer of compound **8** through DASA cyclization (light-control of location), we studied the possibility of controlling host–guest binding of compound **8** in water (light control of function). CDs are well known as host molecules with a multitude of studied guest molecules^57^,^58^,^60^. α-CD binds *trans*-azobenzenes but not *cis*-azobenzenes^59^,^62^.

**Figure 8:**
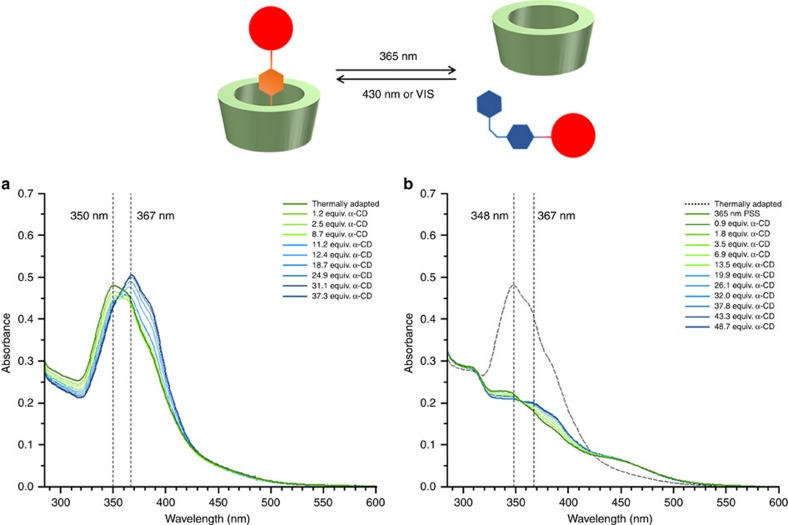
Binding between azobenzene and α-CD. Binding studies on the host–guest binding of α-CD and compound **8**. Titration of α-CD to an aqueous solution (pH ⩾9) of compound **8** (11 μM). A clear change of the absorption spectrum is observed, indicating the binding of the *trans*-azobenzene moiety of **8** (**a**). Binding is significantly reduced in a photostationary state reached under 365 nm irradiation (inducing *trans*–*cis* isomerization; **b**).

Compound **8** was isolated after phase transfer and was then subjected to binding studies with α-CD in water (pH ⩾9; ref. 57). A clear change in the absorption spectrum upon titration of the host was observed (Supplementary Fig. 63) for the thermally adapted compound **8**, indicating the binding of *trans*-azobenzene moiety of **8** to α-CD. For irradiated samples, this effect was markedly reduced (Supplementary Figs 64–67), suggesting a much weaker binding of the *cis*-azobenzene moiety of **8** to α-CD. Photoswitching is retained even in the bound state, where interestingly different PSS values are observed (Supplementary Figs 68–70). Thus, by switching the azobenzene moiety, the affinity for the α-CD host can be tuned (Fig. 8).

Overall, the azobenzene moiety of compound **8** can be independently controlled by light in the organic phase with irradiation at *λ*=365 nm (*trans–cis* isomerization; Figs 5b and 7) and *λ*=430 nm (*cis–trans* isomerization; Fig. 5b). Upon switching of the DASA moiety with white light, compound **8** undergoes phase transfer to the basic aqueous layer. Herein, the *trans*-azobenzene moiety binds to α-CD more strongly than the *cis*-azobenzene one. Compound **8** can be recovered from the aqueous layer by back-extraction to dichloromethane. In summary, compound **8** incorporates the two independently photocontrolled, functionally different capabilities for phase transfer, induced by DASA cyclization and host–guest binding, dependent on the geometry of the azobenzene moiety.

## Discussion

In conclusion, an intermolecular two-switch system consisting of a DASA and an azobenzene was developed by a rational, spectra-guided design. The robustness of the photoswitch combination is illustrated by the compatibility of differently substituted azobenzenes, as well as DASAs. The flexibility in functional group substitution emphasizes the generality and modularity of this approach and may potentially also allow combination with other classes of photoswitches or PPGs. Furthermore, control of thermal relaxation allows specific tailoring of the switch to the needs of the system. Recent non-orthogonal applications of independent switches in the same solution highlight the great potential of our approach^63^,^64^,^65^,^66^,^67^,^68^. The intramolecular photoswitching extends the applicability of this combination, which was exemplified by a dual functional molecule where both the binding to α-CD and phase transfer can be controlled with different wavelengths of light. The intramolecular combination shows interesting switching behaviour dependent on the linker length—an aspect that will be studied in-depth in the future. The presented study show levels of orthogonal selectivity that are unparalleled both for wavelength-selective uncaging^8^ and multiphotochromes^26^. It represents a major step towards the development of future orthogonal and reversible photoswitchable tools that can be used for non-invasively interfering with and the manipulation of multifunctional molecular systems. Future research should focus on overcoming the strong solvent dependence of the photoswitching of the DASAs and orthogonal switching in the near infrared region of the spectrum^38^,^39^,^40^,^41^.

## Methods

### Synthesis of photoswitches and combinations thereof

The synthesis of compounds **1**–**9** is detailed in Supplementary Methods. For NMR characterization of the described compounds, see Supplementary Figs 71–83.

### Photochemical characterization

Irradiation of samples in toluene (Uvasol) was performed with different light sources and filters (Supplementary Tables 1 and 2). Photoswitching was observed with an Agilent 8453 UV-Visible Spectrophotometer in a 10 mm quartz cuvette using Uvasol grade solvents. Generally, the concentrations of the photoswitches were adjusted to result in a measured absorbance between 0.5 and 1.0 (a.u.). Half-lives of photoswitches were measured at *λ*_max_ and fitted with single exponential process.

For the photochemical characterization of photoswitches **1**–**9**, see Supplementary Figs 1–31. For the determination of photostationary states reached, see Supplementary Figs 32–38.

### Determination of quantum yields

The quantum yield was determined by following initial photoswitching kinetics (percentage of switching ∼10%) as decrease in absorbance at *λ*_max_ and using the light intensity determined from standard ferrioxalate actinometry^69^. A more detailed description can be found in Supplementary Methods.

### Photoswitching experiments

Each mixture of compounds was analysed by measuring ultraviolet-visible spectra of the different accessible states and by kinetic measurement of the time-dependent reversible photochromism. Henceforth, each photoswitch was followed at its *λ*_max_. Importantly, samples were irradiated during measurements at an ∼90° angle to the light path of the ultraviolet-visible spectrometer with distances from light source and their respective intensities at this distance detailed in Supplementary Tables 1 and 2. For the photochemical characterization of two-photoswitch mixtures, see Supplementary Figs 39–50. For photoswitching at higher concentrations, see Supplementary Figs 51–53. For the photochemical characterization of the intramolecular combinations of both switches, see Supplementary Figs 54–58.

### Model application studies

For an analysis of the phase-transfer behaviour of compounds **3** and **9**, see Supplementary Fig. 59. For the phase-transfer behaviour of compound **8**, see Supplementary Fig. 60. Experimental details for the binding studies and switching behaviour of the azobenzene moiety of ***cyclized*****-8** can be found in the Supplementary Methods section and Supplementary Figs 61–70 and Supplementary Tables 3–7.

### Data availability

The authors declare that all data supporting the findings of this study are available within the article and its Supplementary Information Files.

## Additional information

**How to cite this article**: Lerch, M. M. *et al*. Orthogonal photoswitching in a multifunctional molecular system. *Nat. Commun.* 7:12054 doi: 10.1038/ncomms12054 (2016).

## Supplementary Material

Supplementary Information Supplementary Figures 1-83, Supplementary Tables 1-7, Supplementary Methods and Supplementary References

## Figures and Tables

**Table 1:** Properties of photoswitches **1** and **3**.

**Entry**	***λ*** **(nm)**	***Open*-DASA 1, ɛ** **(10**^**2 **^**M**^**−1 **^**cm**^**−1**^)	***Trans*-azobenzene 3, ɛ** **(10**^**2 **^**M**^**−1 **^**cm**^**−1**^)
**1**	360	<3	356.5±4.1
**2**	365	<3	349.4±4.2
**3**	370	<3	324.8±4.1
**4**	430	<3	17.0±0.8
**5**	445	5.7±1.5	16.7±0.7
**6**	515	279.7±2.9	4.3±0.7
**7**	570	1758.0±15.3	<3

DASA, donor–acceptor Stenhouse adduct.

Overview of molar absorptivities of photoswitches **1** and **3** at relevant wavelengths.

**Table 2:** Overview of photoswitch characteristics.

**Entry**	**Switch**	***t***_**1/2**_	***λ***_**max**_ **(nm)**	**PSS for irradiation at** λ**			
				**365**	**430**	**590**	**White light**
**1**	Azo **3**	>8 h	360 (*π*–π*)	4:96	70:30	ND	77:23
			445 (*n*–π*)				
**2**	Azo **4**	180 s	434 (*π*–π*)	ND	ND	ND	ND
			374 (*n*–π*)				
**3**	Azo **5**	>8 h	320 (*π*–π*)	32:68†	82:18	ND	77:23
			436 (*n*–π*)				
**4**	Azo **6**	>8 h	348 (*π*–π*)	<3:97	69:31	ND	76:24
			444 (*n*–π*)				
**5**	DASA **1**	37 s	570	ND	ND	<20:80‡	<20:80‡
**6**	DASA **2**	130 s	545	ND	ND	<20:80‡§	<20:80^‡^

DASA, donor–acceptor Stenhouse adduct; ND, not determined; PSS, photostationary state.

^**^Trans/cis

^†^Same for 312 nm.

^‡^Open/cyclized.

^§^546 nm.
