# Supplementary material for: Orthogonal photoswitching in a multifunctional molecular system
Source: Nat Commun. 2016 Jul 12;7:12054. doi: 10.1038/ncomms12054 (PMC4945879; doi:10.1038/ncomms12054)
Supplement: Supplementary Information — Supplementary Figures 1-83, Supplementary Tables 1-7, Supplementary Methods and Supplementary References [file ncomms12054-s1.pdf]

UV-vis Spectra and Photoswitching

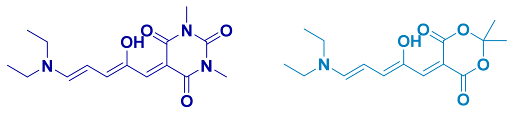

1 2

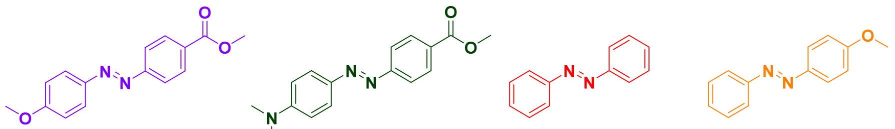

3 4 5 6

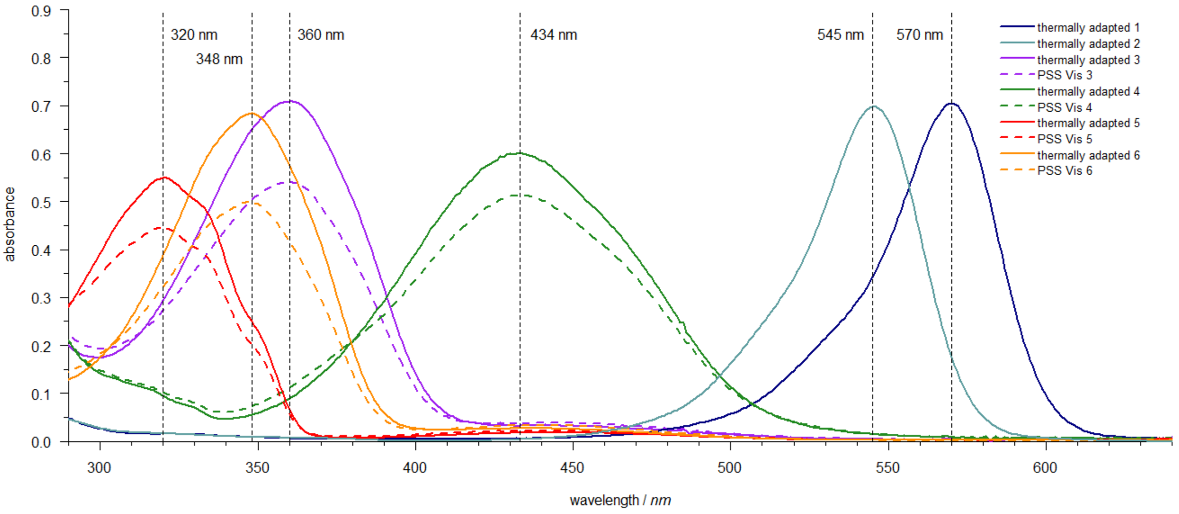

Supplementary Figure 1 | Overlay of the UV-vis absorption spectra of compound **1** – **6** (compound **1** and **2**: ~4 µM; compounds **3** – **6**: ~20 µM; toluene; room temperature) with their corresponding absorption maxima. Both the thermally adapted and photostationary states under white light irradiation (PSS) are indicated.

Photochemical Characterization of Compounds 1 – 9

Compound 1

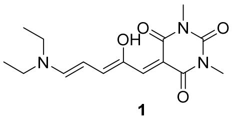

1

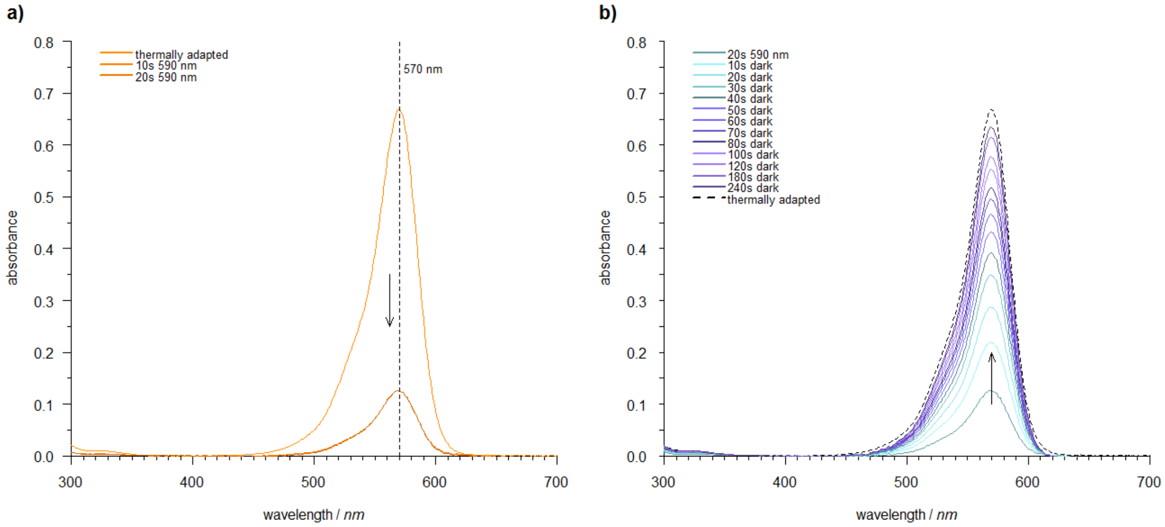

Supplementary Figure 2 | Absorption spectra for the photoisomerization of compound **1** (λ_{max} = 570 nm; ~4 µM in toluene; room temperature): a) cyclization with λ = 590 nm (Supplementary Table **1**, entry **10**; 604-APTD1608SYC/J3,) and b) thermal relaxation. Irradiation times are indicated.

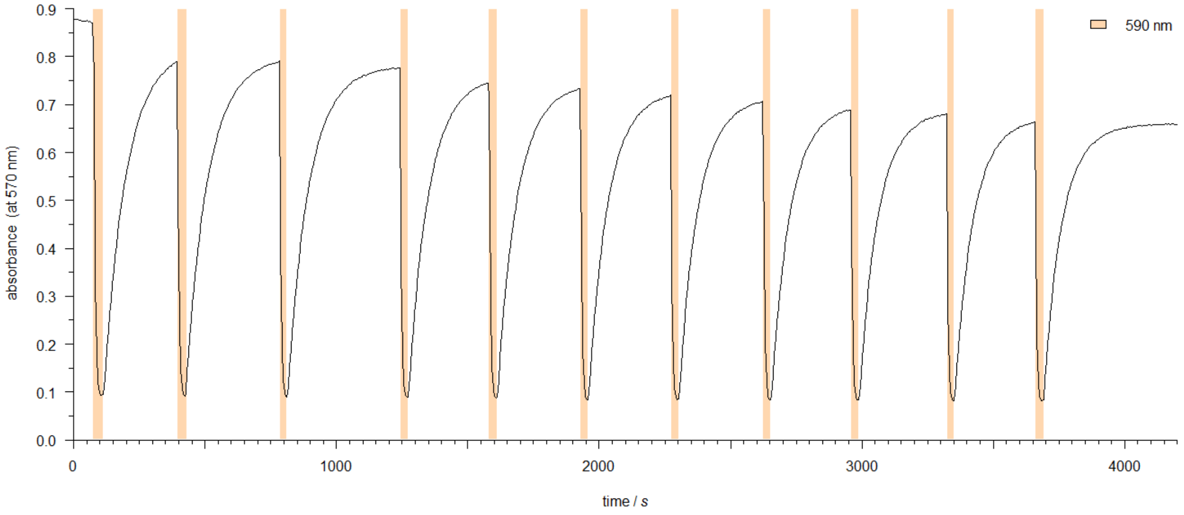

Supplementary Figure 3 | Reversible photochromism for repeated switching cycles of compound **1** (~4 µM in toluene; room temperature) observed at λ_{max} = 570 nm: Switching with λ = 590 nm (Supplementary Table **1**, entry **10**; 604-APTD1608SYC/J3,) and thermal relaxation.

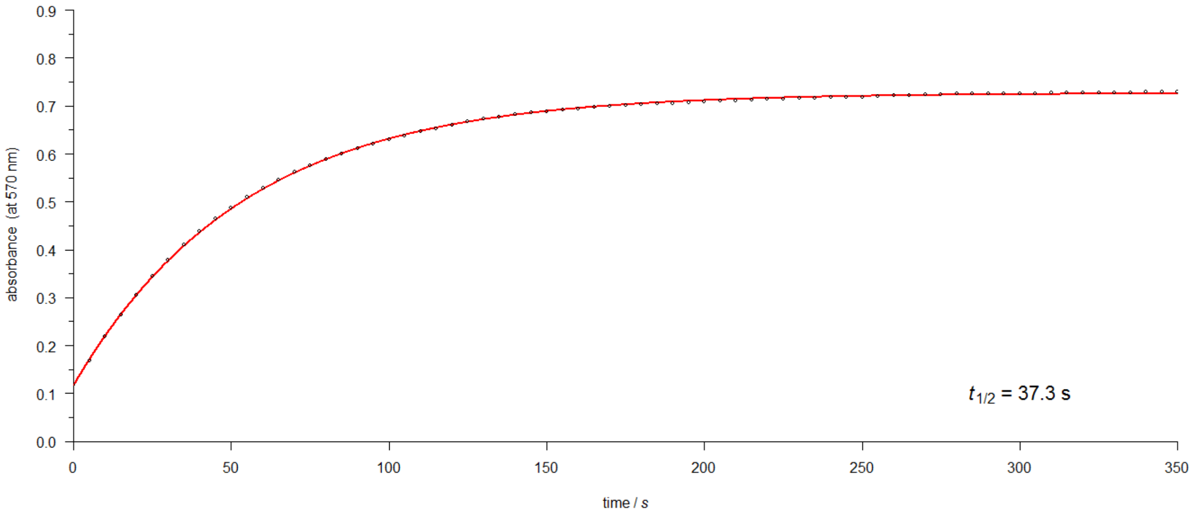

Supplementary Figure 4 | Determination of half-life for compound **1**: Points correspond to measured data (observed at λ_{max} = 570 nm, toluene, ~4 µM); line represents the fitting with single exponential process. The half-life was determined to: $t_{1/2}$ = 37.3 s.

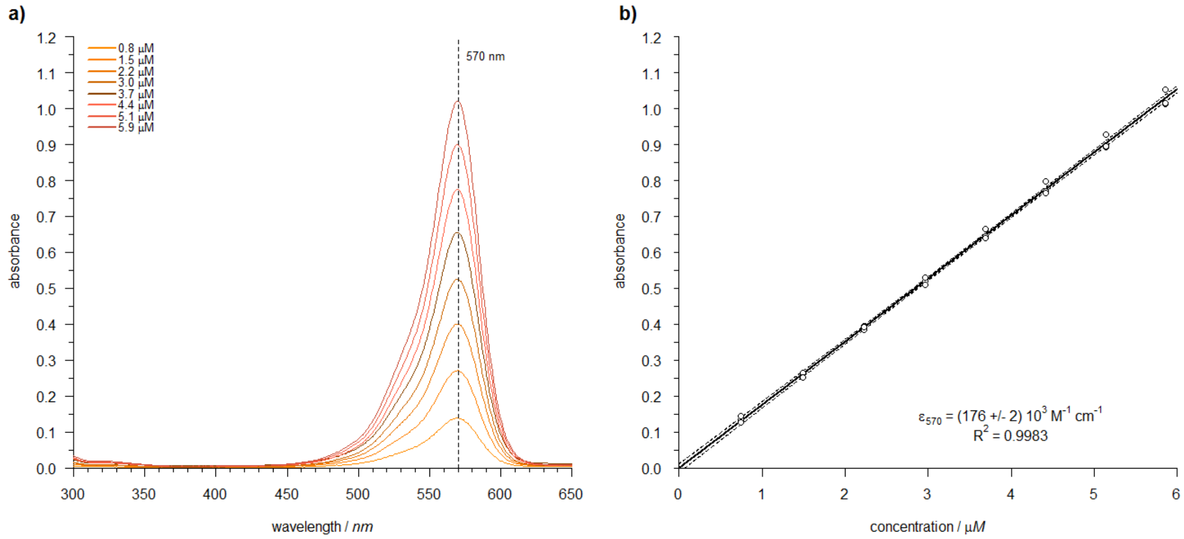

Supplementary Figure 5 | a) No significant shift of the λ_{max} is observed in a series of UV-vis absorption spectra of increasing concentrations. b) Determination of the molar extinction coefficient ε_{570} of compound **1** in triplicates (linear regression, consider each replicate value as single point; 95% confidence intervals are indicated with dashed lines).

Compound 2

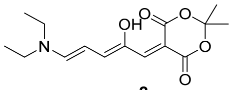

2

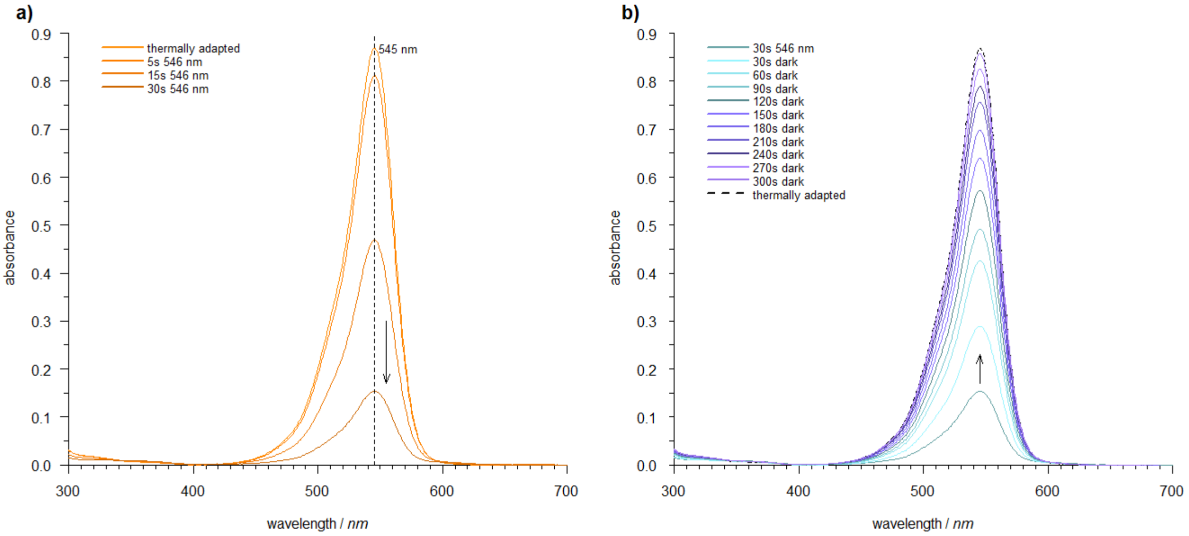

Supplementary Figure 6 | Absorption spectra for the photoisomerization of compound **2** (λ_{max} = 545 nm; ~4 µM in toluene; room temperature): a) cyclization with λ = 546 nm (Supplementary Table **2**, entry **2**; 546FS10-50,) and b) thermal relaxation. Irradiation times are indicated.

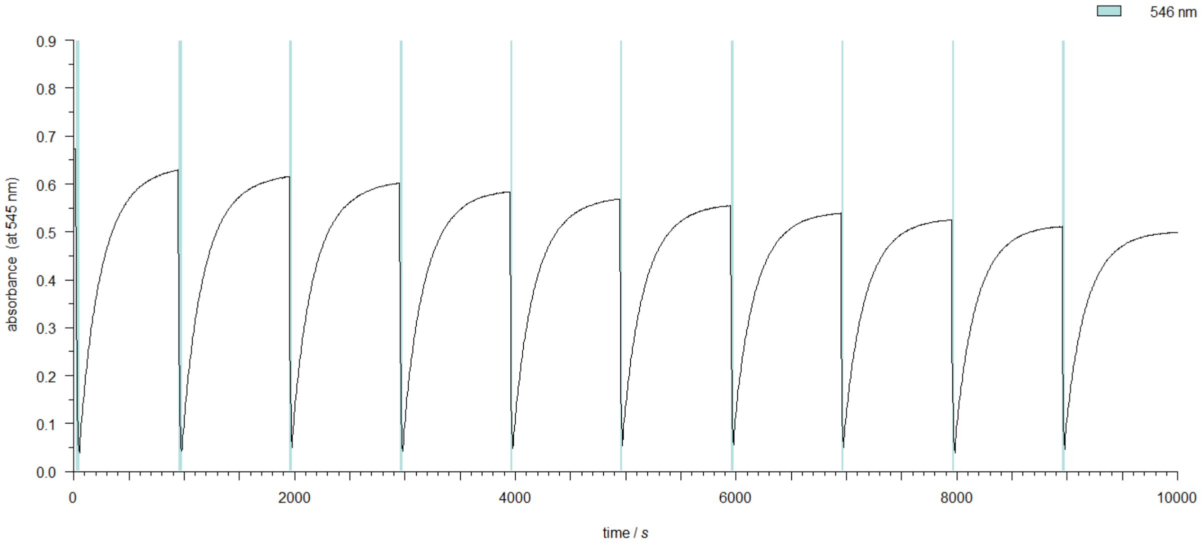

Supplementary Figure 7 | Reversible photochromism for repeated switching cycles of compound **2** (~4 µM in toluene; room temperature) observed at λ_{max} = 545 nm: Switching with λ = 546 nm (Supplementary Table **2**, entry **2**; 546FS10-50,) and thermal relaxation.

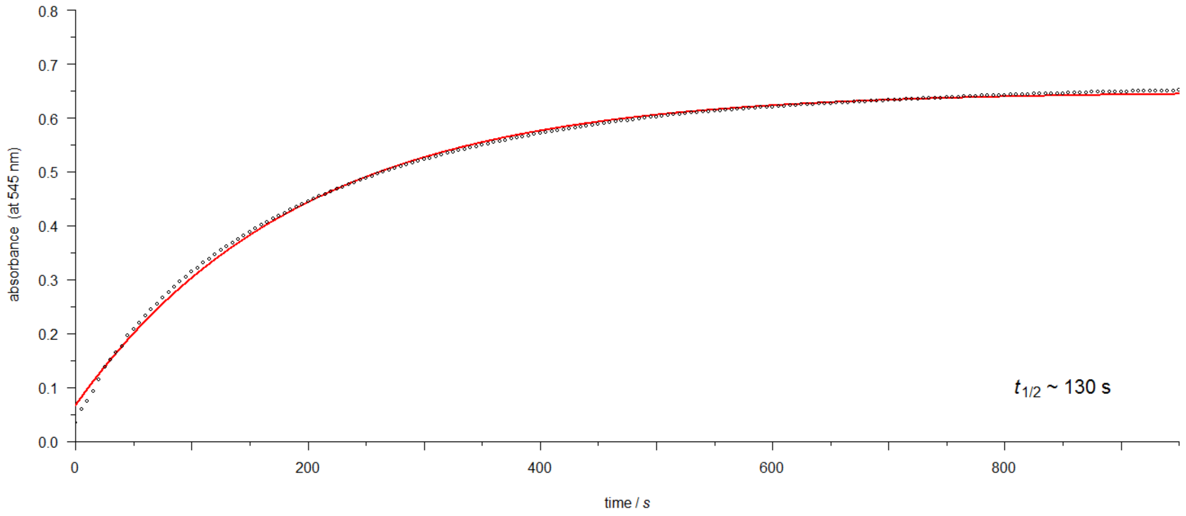

Supplementary Figure 8 | Determination of half-life for compound **2**: Points correspond to measured data (observed at λ_{max} = 545 nm, toluene, ~4 µM); line represents the fitting with single exponential process. The half-life was determined to: $t_{1/2}$ ~ 130 s.

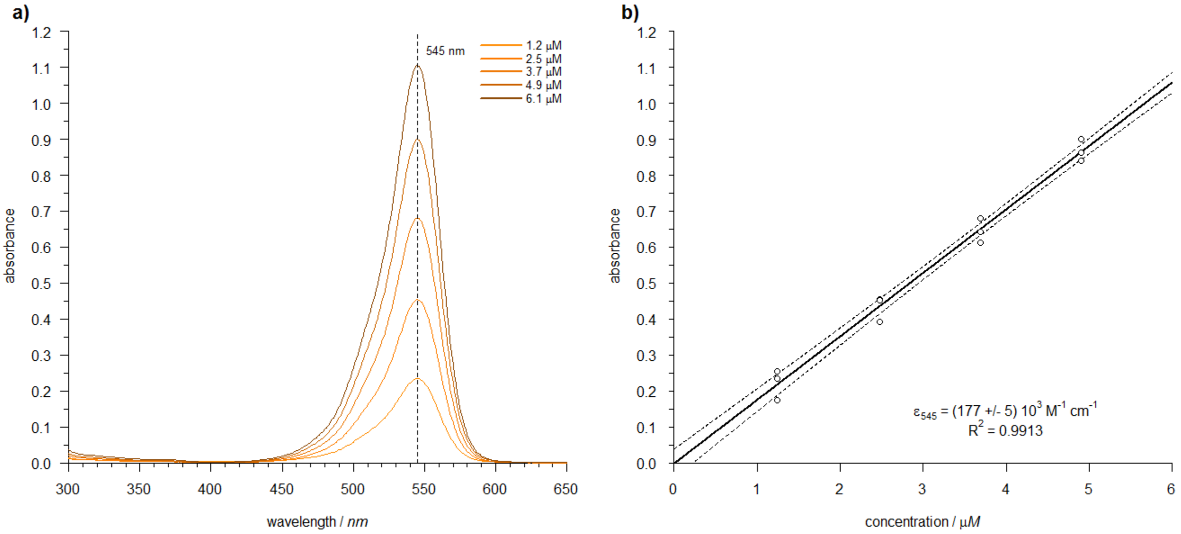

Supplementary Figure 9 | a) No significant shift of the λ_{max} is observed in a series of UV-vis absorption spectra of increasing concentrations. b) Determination of the molar extinction coefficient ε_{545} of compound **2** in triplicates (linear regression, consider each replicate value as single point; 95% confidence intervals are indicated with dashed lines).

Compound 3

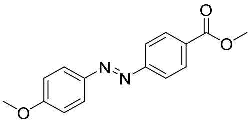

3

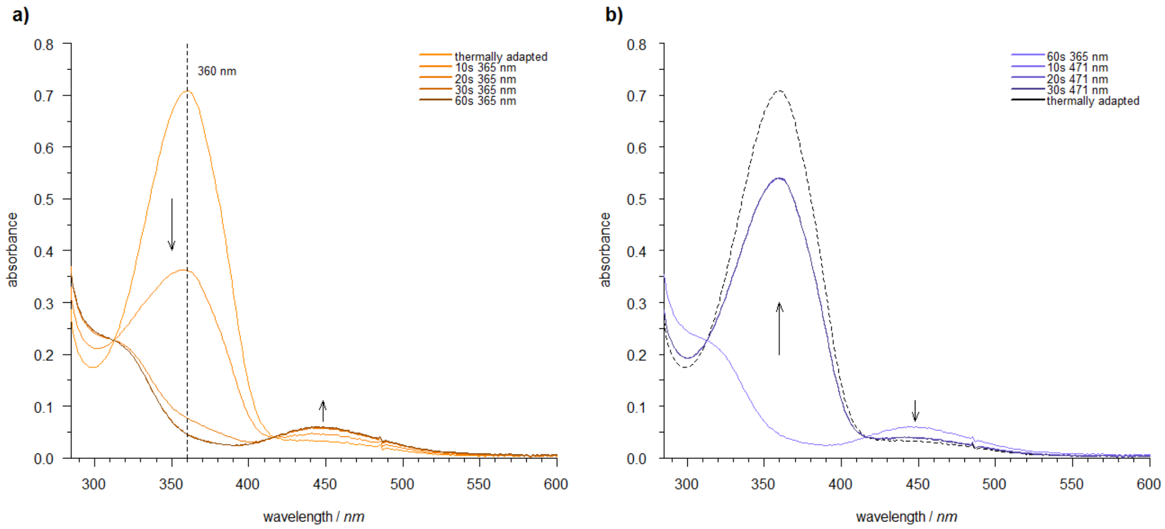

Supplementary Figure 10 | Absorption spectra for the photoisomerization of compound **3** (λ_{max} = 360 nm; ~20 µM in toluene; room temperature): a) switching *trans* to *cis* with λ = 365 nm (br.) (Supplementary Table **1**, entry **2**; ENB-280C/FE,) and b) *cis* to *trans*, with λ = 471 nm (Supplementary Table **1**, entry **8**; 859-LTL2R3TBV3KS,). Irradiation times are indicated.

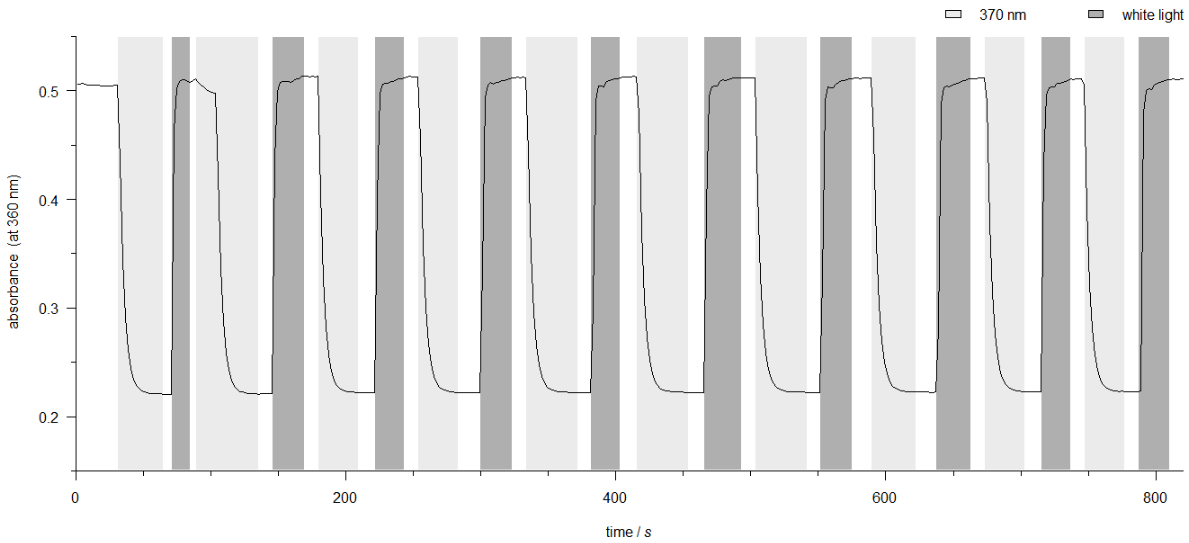

Supplementary Figure 11 | Reversible photochromism for repeated switching cycles of compound **3** (~20 µM in toluene; room temperature) observed at λ_{max} = 360 nm: Switching with λ = 370 nm (*trans-cis*; Supplementary Table **1**, entry **4**; MARL 260019 UV EMITTER, TO-46, 100DEG,) and switching back with white light (*cis-trans*; Supplementary Table **1**, entry **12**; OSL1-EC,).

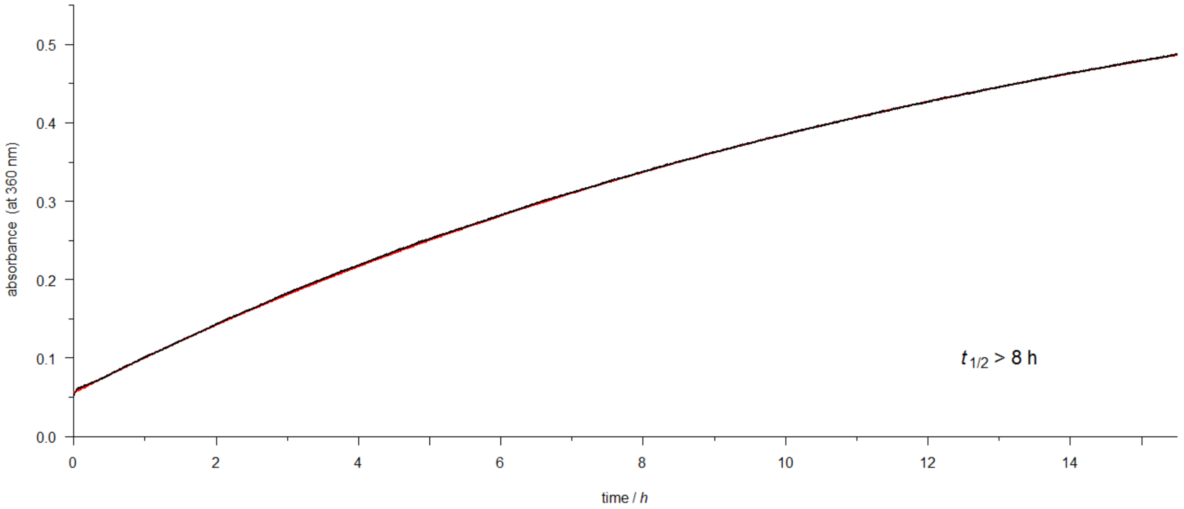

Supplementary Figure 12 | Determination of half-life for compound **3**: Points correspond to measured data (observed at λ_{max} = 360 nm, toluene, ~20 µM); line represents the fitting with single exponential process. The half-life was determined to: $t_{1/2}$ > 8 h.

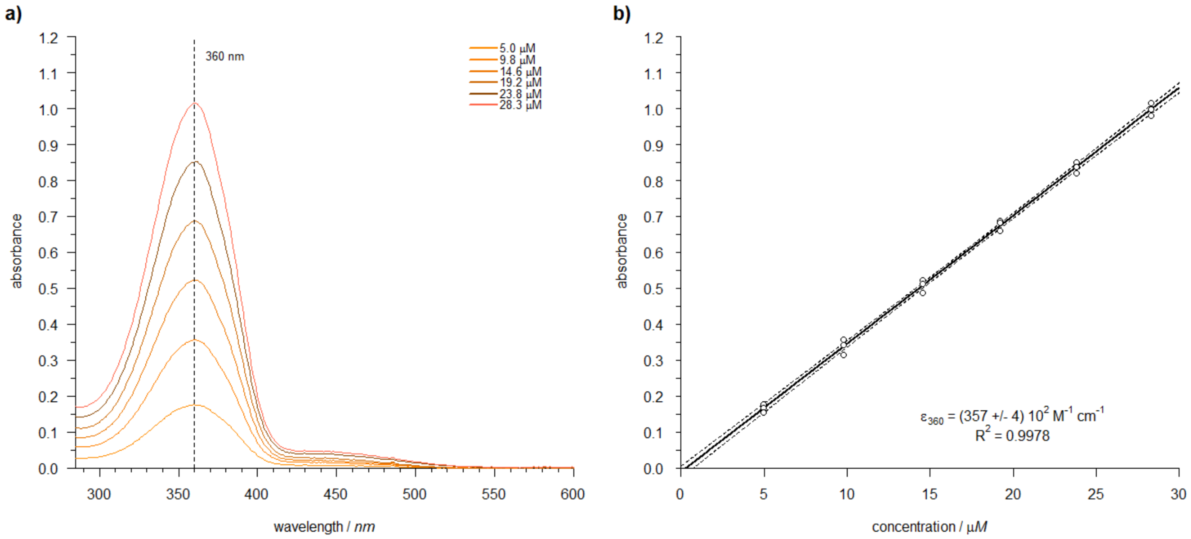

Supplementary Figure 13 | a) No significant shift of the λ_{max} is observed in a series of UV-vis absorption spectra of increasing concentrations. b) Determination of the molar extinction coefficient ε_{360} of compound **3** in triplicates (linear regression, consider each replicate value as single point; 95% confidence intervals are indicated with dashed lines).

Compound 4

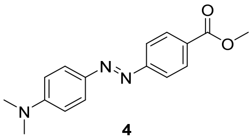

4

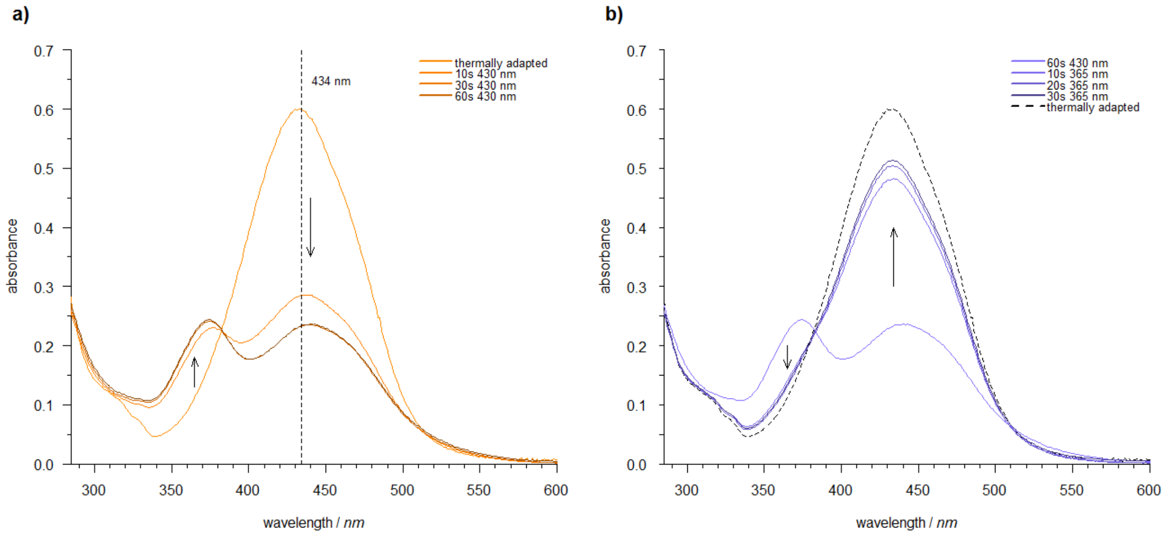

Supplementary Figure 14 | Absorption spectra for the photoisomerization of compound **4** (λ_{max} = 434 nm; ~20 µM in toluene; room temperature): a) switching *trans* to *cis* with λ = 430 nm (Supplementary Table **2**, entry **1**; 430FS10-50,) and b) *cis* to *trans*, with λ = 365 nm (Supplementary Table **1**, entry **3**; M365F1,). Irradiation times are indicated.

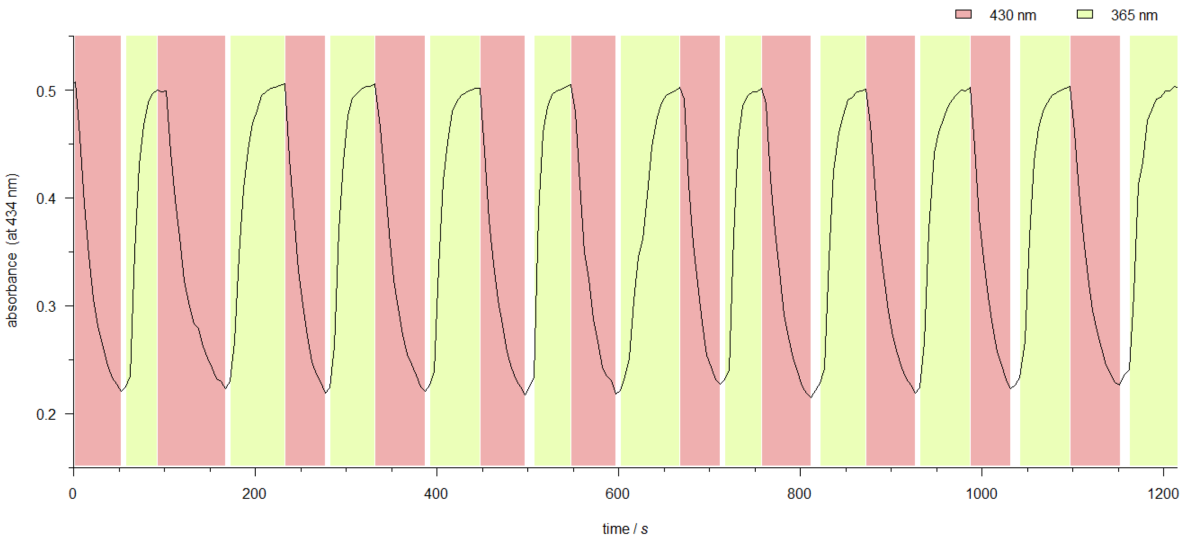

Supplementary Figure 15 | Reversible photochromism for repeated switching cycles of compound **4** (~20 µM in toluene; room temperature) observed at λ_{max} = 434 nm: Switching with λ = 430 nm (*trans*-*cis*; Supplementary Table **2**, entry **1**; 430FS10-50,) and switching back with λ = 365 nm (*cis*-*trans*; Supplementary Table **1**, entry **3**; M365F1,).

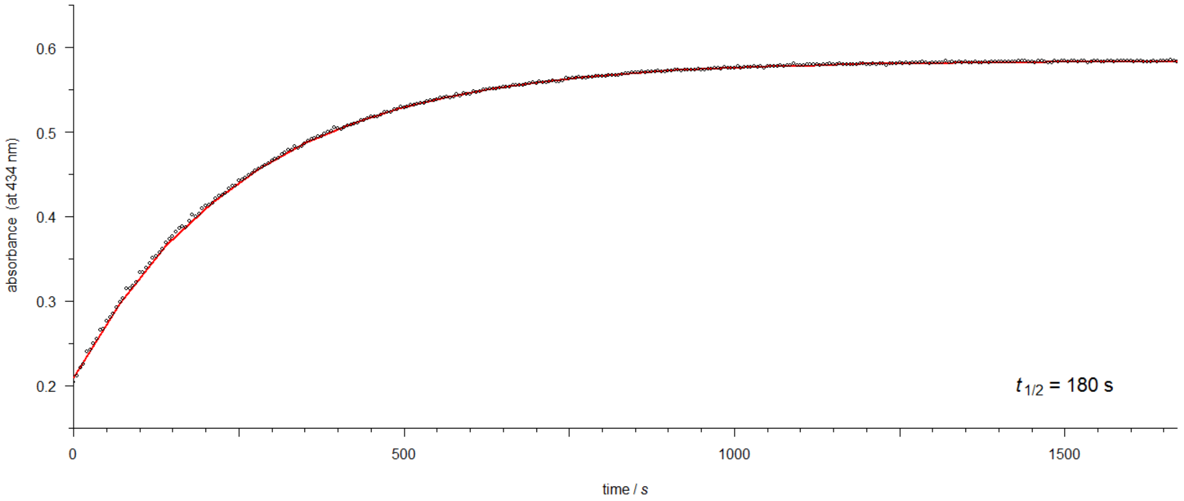

Supplementary Figure 16 | Determination of half-life for compound **4**: Points correspond to measured data (observed at λ_{max} = 434 nm, toluene, ~20 µM); line represents the fitting with single exponential process. The half-life was determined to: $t_{1/2}$ = 180 s.

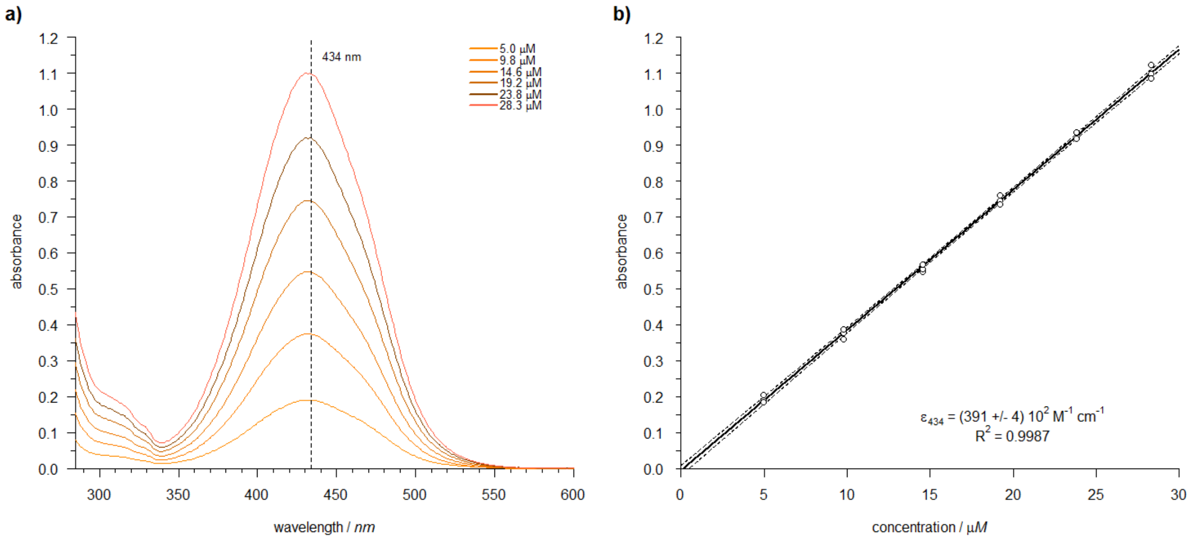

Supplementary Figure 17 | a) No significant shift of the λ_{max} is observed in a series of UV-vis absorption spectra of increasing concentrations. b) Determination of the molar extinction coefficient ε_{434} of compound **4** in triplicates (linear regression, consider each replicate value as single point; 95% confidence intervals are indicated with dashed lines).

Compound 5

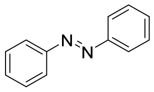

5

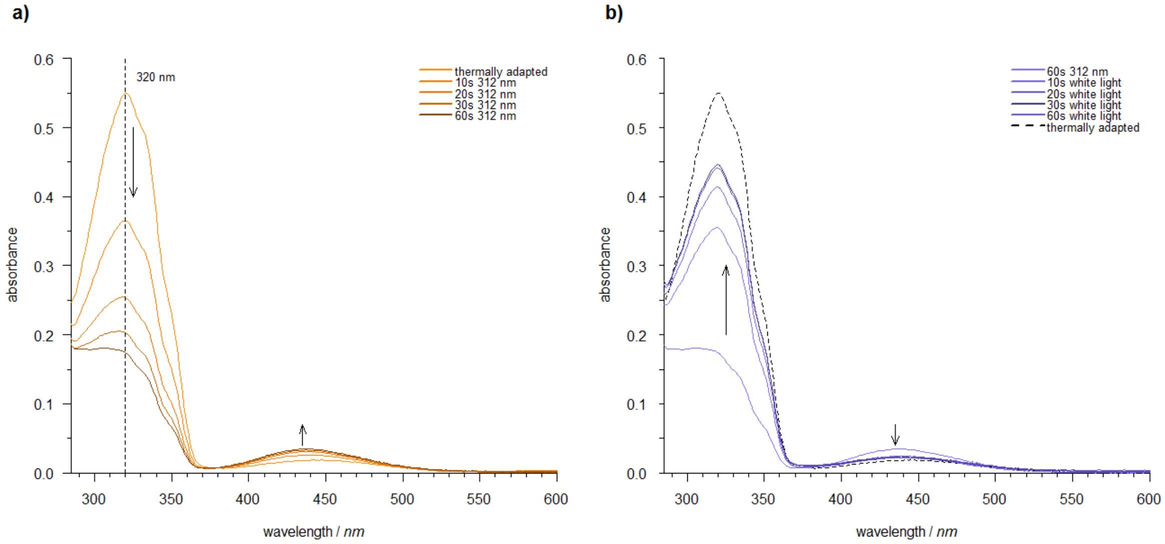

Supplementary Figure 18 | Absorption spectra for the photoisomerization of compound **5** (λ_{max} = 320 nm; ~20 µM in toluene; room temperature): a) switching *trans* to *cis* with λ = 312 nm (Supplementary Table **1**, entry **1**; ENB-280C/FE,) and b) *cis* to *trans*, with white light (*cis-trans*; Supplementary Table **1**, entry **12**; OSL1-EC,). Irradiation times are indicated.

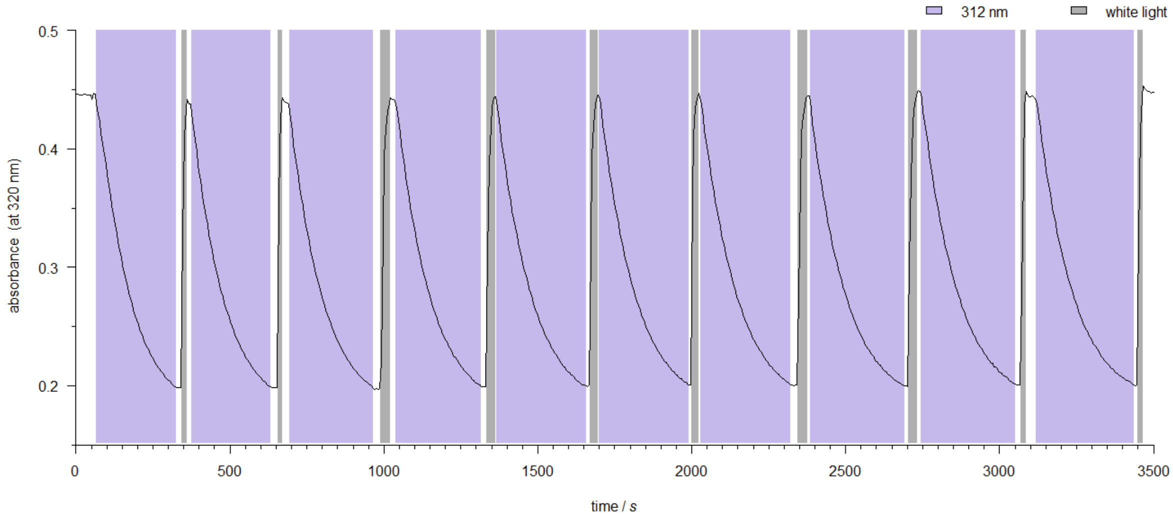

Supplementary Figure 19 | Reversible photochromism for repeated switching cycles of compound **5** (~20 µM in toluene; room temperature) observed at λ_{max} = 320 nm: Switching with λ = 312 nm (*trans-cis*; Supplementary Table **1**, entry **1**; ENB-280C/FE,) and switching back with white light (*cis-trans*; Supplementary Table **1**, entry **12**; OSL1-EC,).

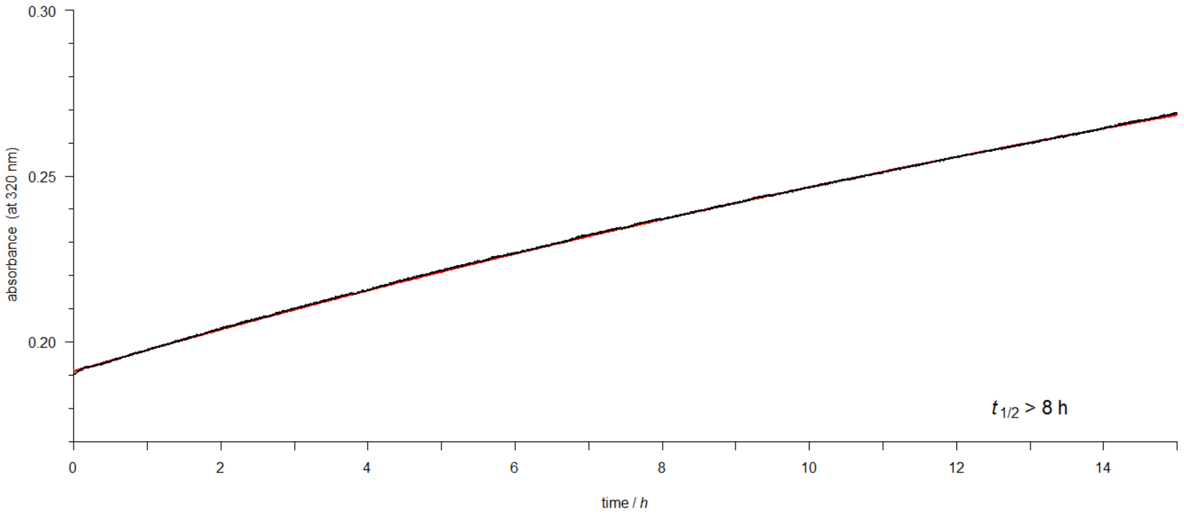

Supplementary Figure 20 | Determination of half-life for compound **5**: Points correspond to measured data (observed at λ_{max} = 320 nm, toluene, ~20 µM); line represents the fitting with single exponential process. The half-life was determined to: $t_{1/2}$ > 8 h.

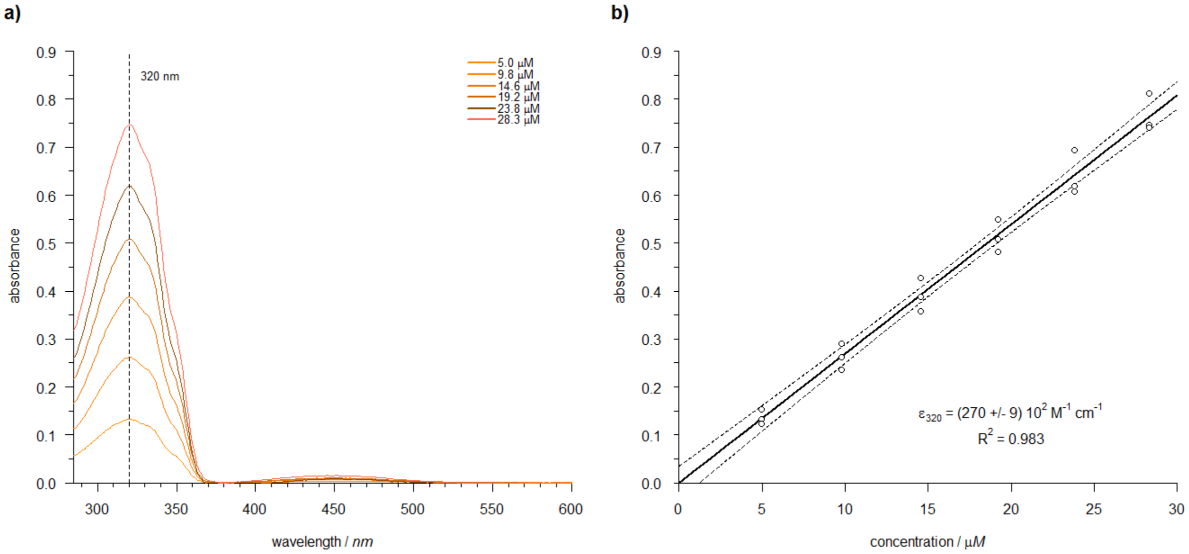

Supplementary Figure 21 | a) No significant shift of the λ_{max} is observed in a series of UV-vis absorption spectra of increasing concentrations. b) Determination of the molar extinction coefficient ε_{320} of compound **5** in triplicates (linear regression, consider each replicate value as single point; 95% confidence intervals are indicated with dashed lines).

Compound 6

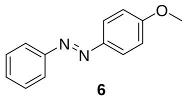

6

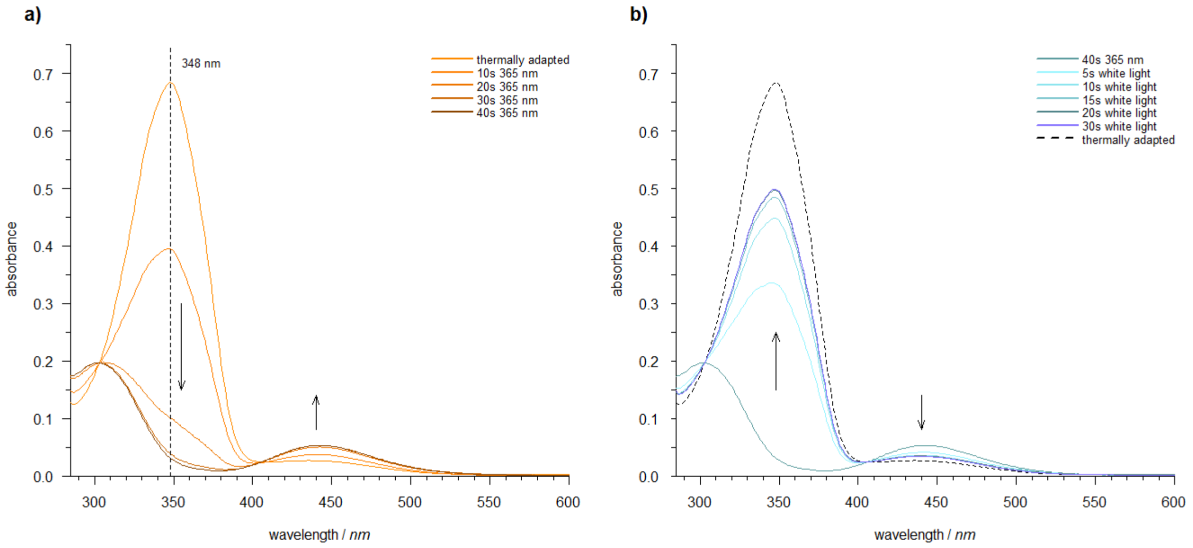

Supplementary Figure 22 | Absorption spectra for the photoisomerization of compound **6** (λ_{max} = 348 nm; ~20 µM in toluene; room temperature): a) switching *trans* to *cis* with λ = 365 nm (br.) (Supplementary Table **1**, entry **2**; ENB-280C/FE,) and b) *cis* to *trans*, with white light (Supplementary Table **1**, entry **12**; OSL1-EC,). Irradiation times are indicated.

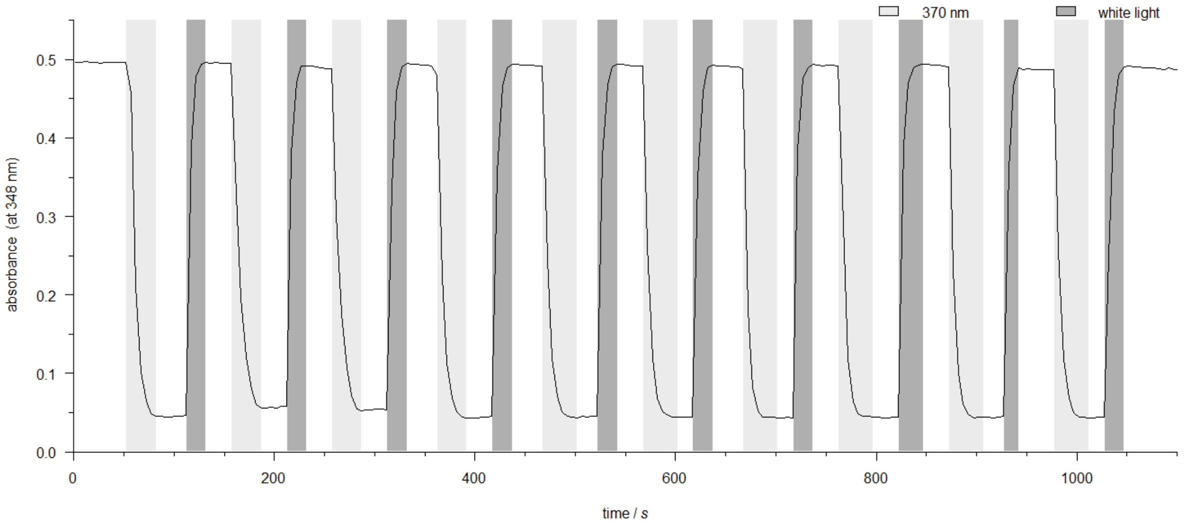

Supplementary Figure 23 | Reversible photochromism for repeated switching cycles of compound **6** (~20 µM in toluene; room temperature) observed at λ_{max} = 348 nm: Switching with λ = 370 nm (*trans-cis*; Supplementary Table **1**, entry **4**; MARL 260019 UV EMITTER, TO-46, 100DEG,) and switching back with white light (*cis-trans*; Supplementary Table **1**, entry **12**; OSL1-EC,).

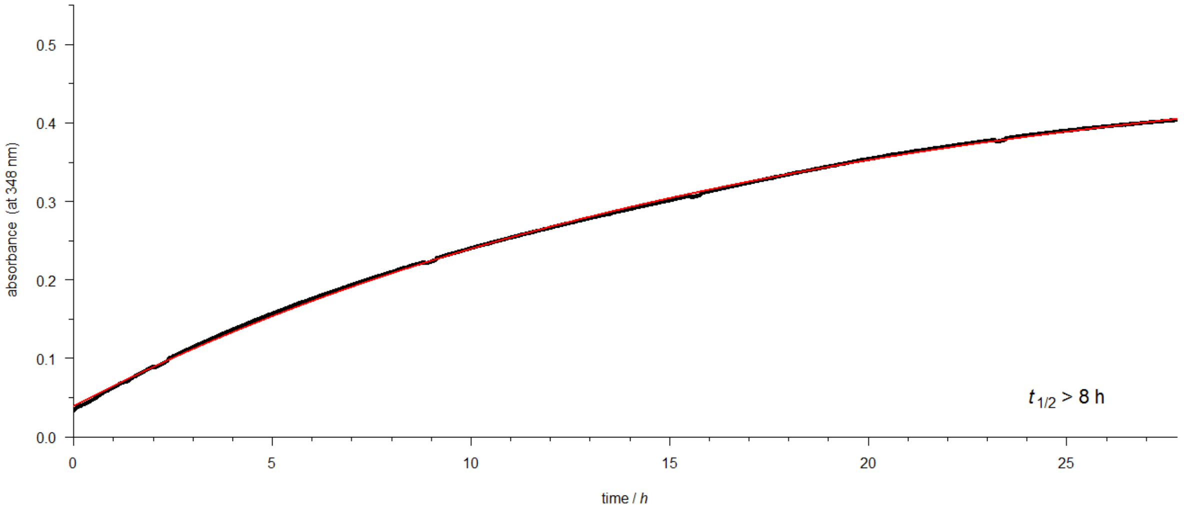

Supplementary Figure 24 | Determination of half-life for compound **6**: Points correspond to measured data (observed at λ_{max} = 348 nm, toluene, ~20 µM); line represents the fitting with single exponential process. The half-life was determined to: $t_{1/2}$ > 8 h.

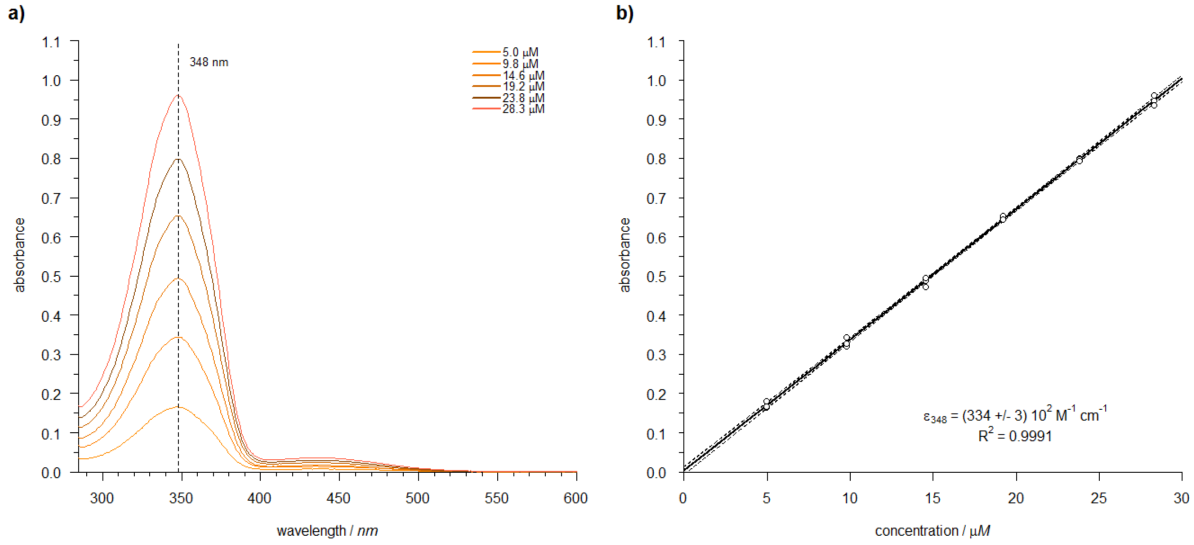

Supplementary Figure 25 | a) No significant shift of the λ_{max} is observed in a series of UV-vis absorption spectra of increasing concentrations. b) Determination of the molar extinction coefficient ε_{348} of compound **6** in triplicates (linear regression, consider each replicate value as single point; 95% confidence intervals are indicated with dashed lines).

Compound S6

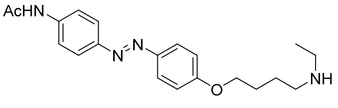

S6

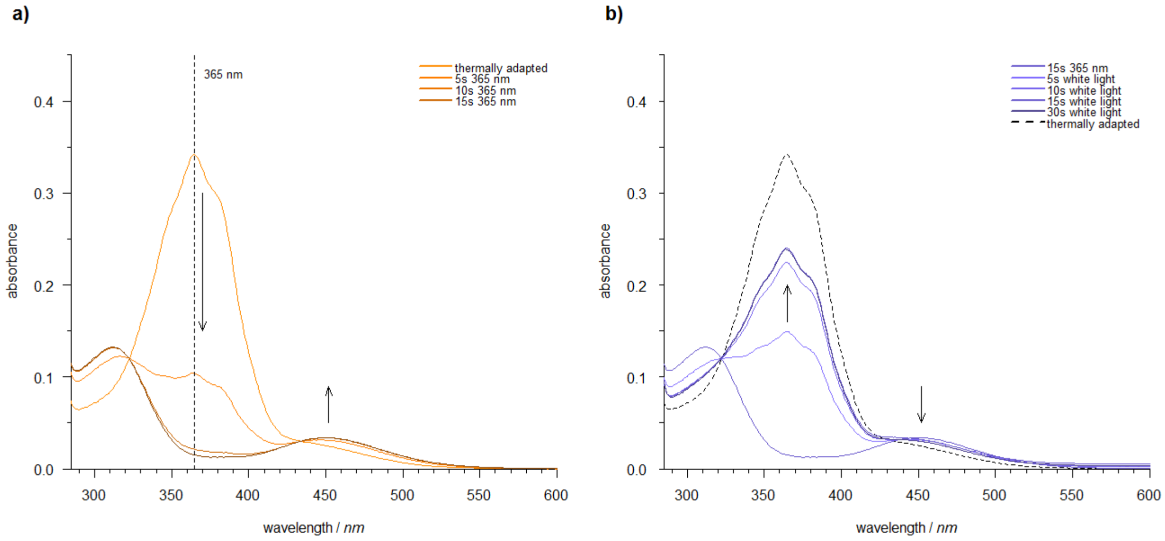

Supplementary Figure 26 | Absorption spectra for the photoisomerization of compound **S6** (λ_{max} = 365 nm; ~10 µM in toluene; room temperature): a) switching *trans* to *cis* with λ = 365 nm (br.) (Supplementary Table **1**, entry **2**; ENB-280C/FE,) and b) *cis* to *trans*, with white light (*cis-trans*; Supplementary Table **1**, entry **12**; OSL1-EC,). Irradiation times are indicated.

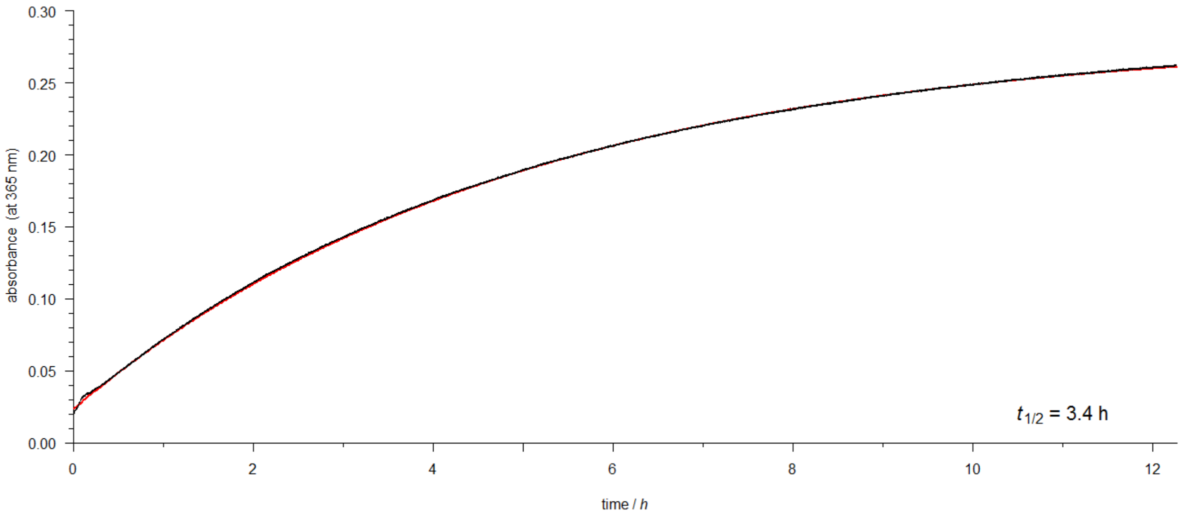

Supplementary Figure 27 | Determination of half-life for compound **S6**: Points correspond to measured data (observed at λ_{max} = 365 nm, toluene, ~10 µM); line represents the fitting with single exponential process. The half-life was determined to: $t_{1/2}$ = 3.4 h.

Compound S8

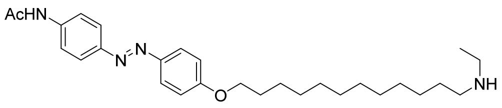

8

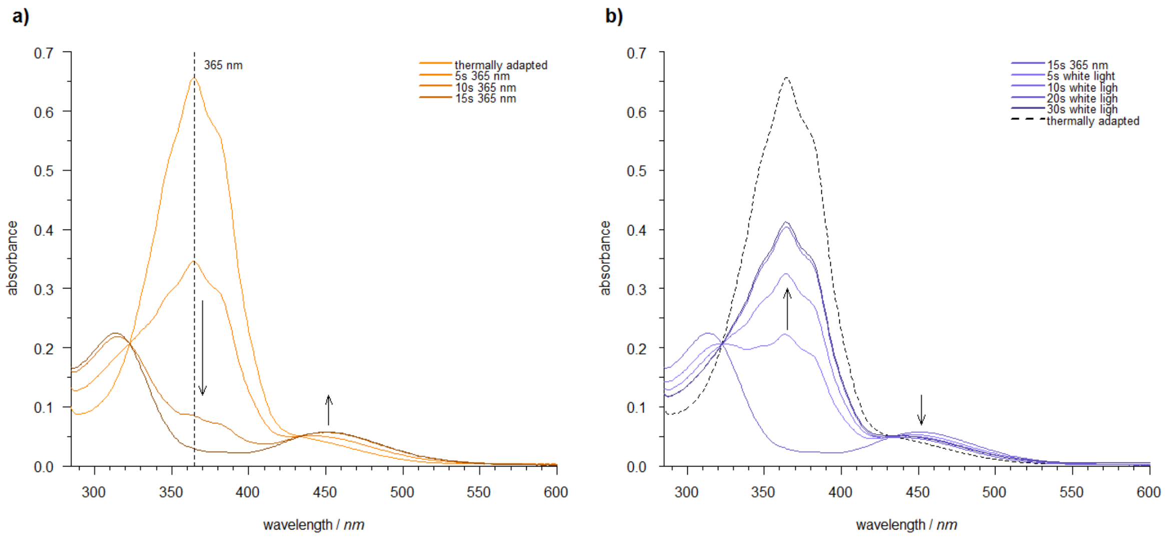

Supplementary Figure 28 | Absorption spectra for the photoisomerization of compound **S8** (λ_{max} = 365 nm; ~20 µM in toluene; room temperature): a) switching *trans* to *cis* with λ = 365 nm (br.) (Supplementary Table **1**, entry **2**; ENB-280C/FE,) and b) *cis* to *trans*, with white light (*cis-trans*; Supplementary Table **1**, entry **12**; OSL1-EC,). Irradiation times are indicated.

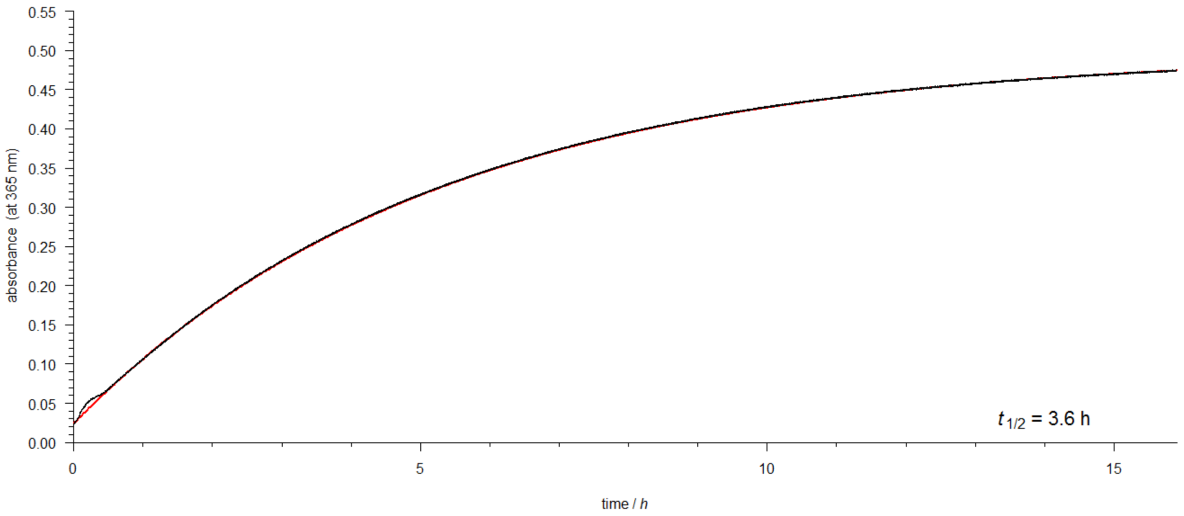

Supplementary Figure 29 | Determination of half-life for compound **S8**: Points correspond to measured data (observed at λ_{max} = 365 nm, toluene, ~20 µM); line represents the fitting with single exponential process. The half-life was determined to: $t_{1/2}$ = 3.6 h.

Compound 9

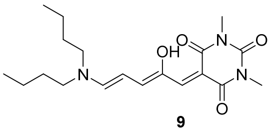

9

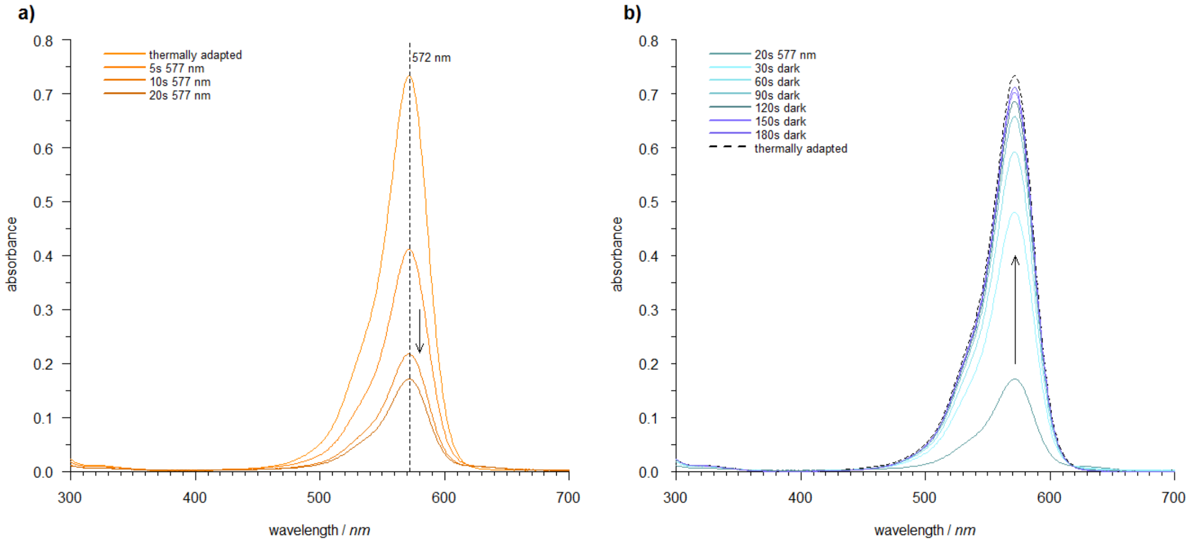

Supplementary Figure 30 | Absorption spectra for the photoisomerization of compound **9** (λ_{max} = 572 nm; ~4 µM in toluene; room temperature): a) cyclization with λ = 577 nm (Supplementary Table **2**, entry **3**; 577FS10-50,) and b) thermal relaxation. Irradiation times are indicated.

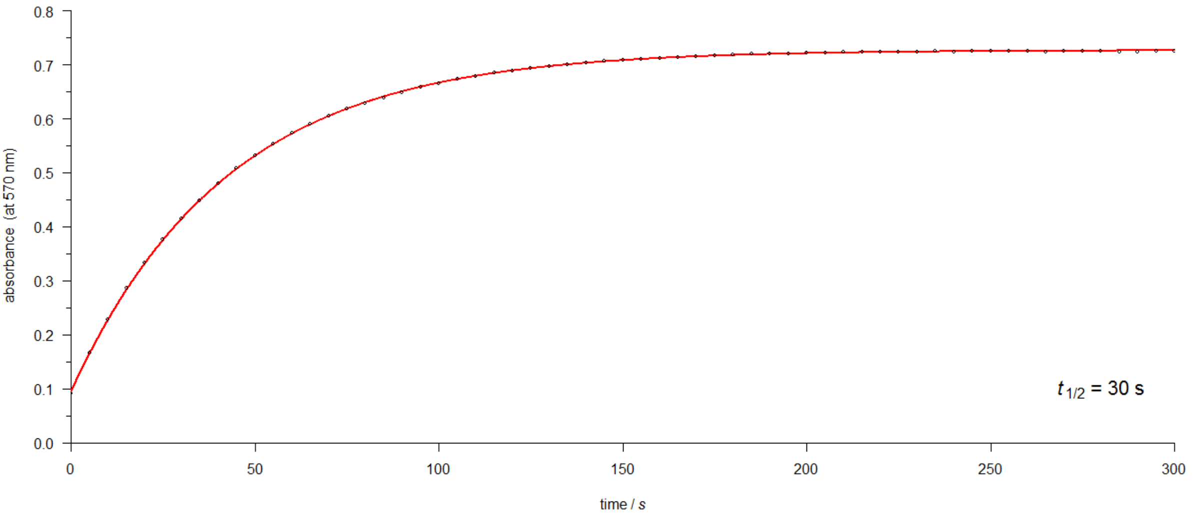

Supplementary Figure 31 | Determination of half-life for compound **9**: Points correspond to measured data (observed at λ_{max} = 570 nm, toluene, ~4 µM); line represents the fitting with single exponential process. The half-life was determined to: $t_{1/2}$ = 30 s.

Determination of Photostationary States for Compounds 3 to 6

Compound 3

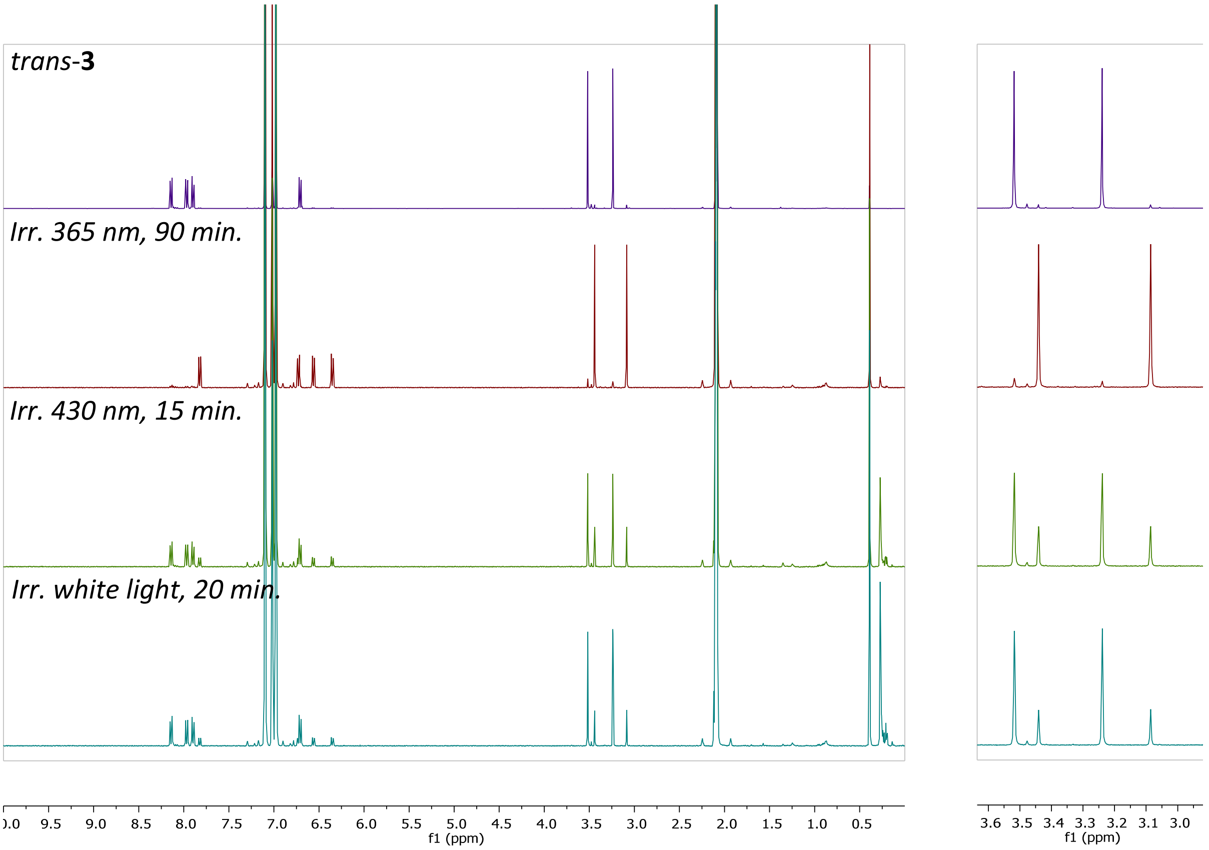

Supplementary Figure 32 | Determination of photostationary states (PSS) for compound **3** by ^{1}H-NMR in toluene-d_8; 2 mM: Irradiation of samples with indicated light sources and irradiation times. After each irradiation, NMR samples were diluted and checked by UV-vis spectroscopy whether the PSS was reached: λ = 365 nm (br.) for 90 min (Supplementary Table **1**, entry **2**; ENB-280C/FE,): 4:96 (*trans/cis*); λ = 430 nm for 15 min (Supplementary Table **1**, entry **6**; 607-4304H6,): 70:30 (*trans/cis*); white light for 20 min (Supplementary Table **1**, entry **12**; OSL1-EC,): 77:23 (*trans/cis*).

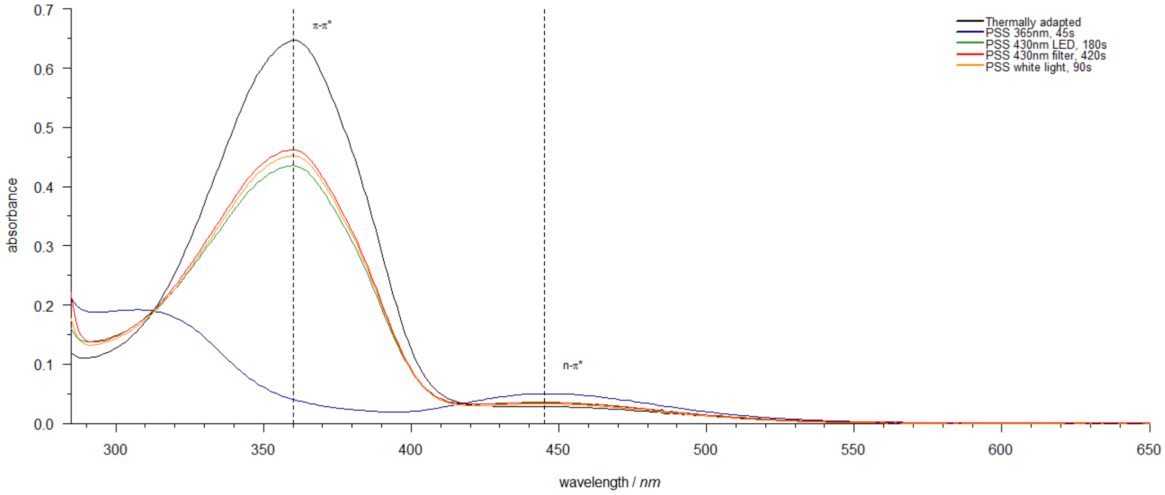

Supplementary Figure 33 | UV-vis absorption spectra illustrating the photostationary states (PSS) for compound **3** (~20 µM in toluene; room temperature). Irradiation times are indicated. Light sources used: λ = 365 nm (br.) (Supplementary Table **1**, entry **2**; ENB-280C/FE,); λ = 430 nm (*Filter,* Supplementary Table **2**, entry **1**; 430FS10-50,); λ = 430 nm (*LED,* Supplementary Table **1**, entry **6**; 607-4304H6,) and white light (Supplementary Table **1**, entry **12**; OSL1-EC,).

Compound 4

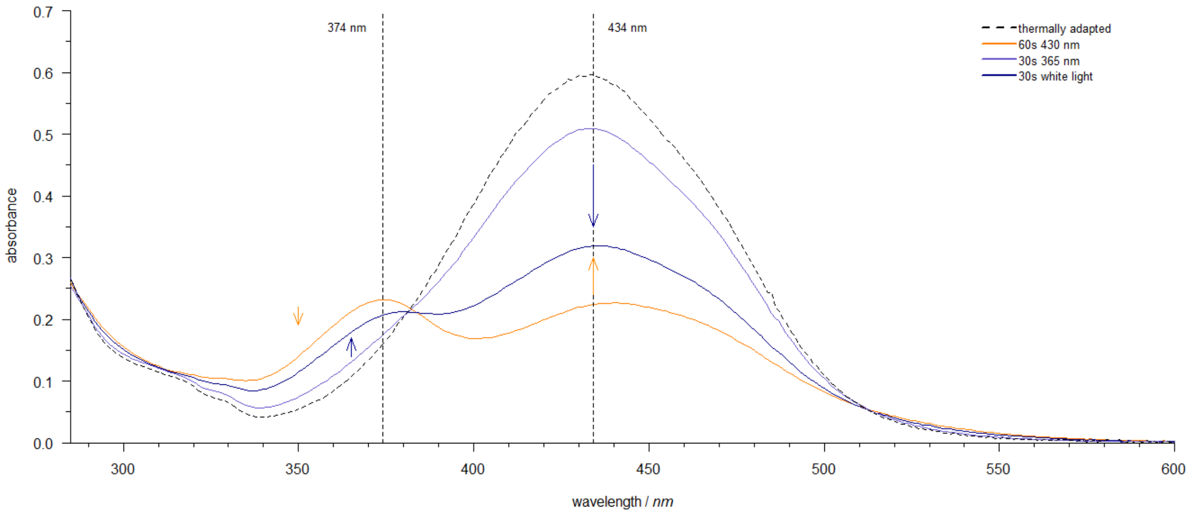

Supplementary Figure 34 | UV-vis absorption spectra illustrating the photostationary states (PSS) for compound **4** (~20 µM in toluene; room temperature). Irradiation times are indicated. Light sources used: λ = 365 nm (Supplementary Table **1**, entry **3**; M365F1,); λ = 430 nm (*Filter,* Supplementary Table **2**, entry **1**; 430FS10-50,) and white light (Supplementary Table **1**, entry **12**; OSL1-EC,).

Compound 5

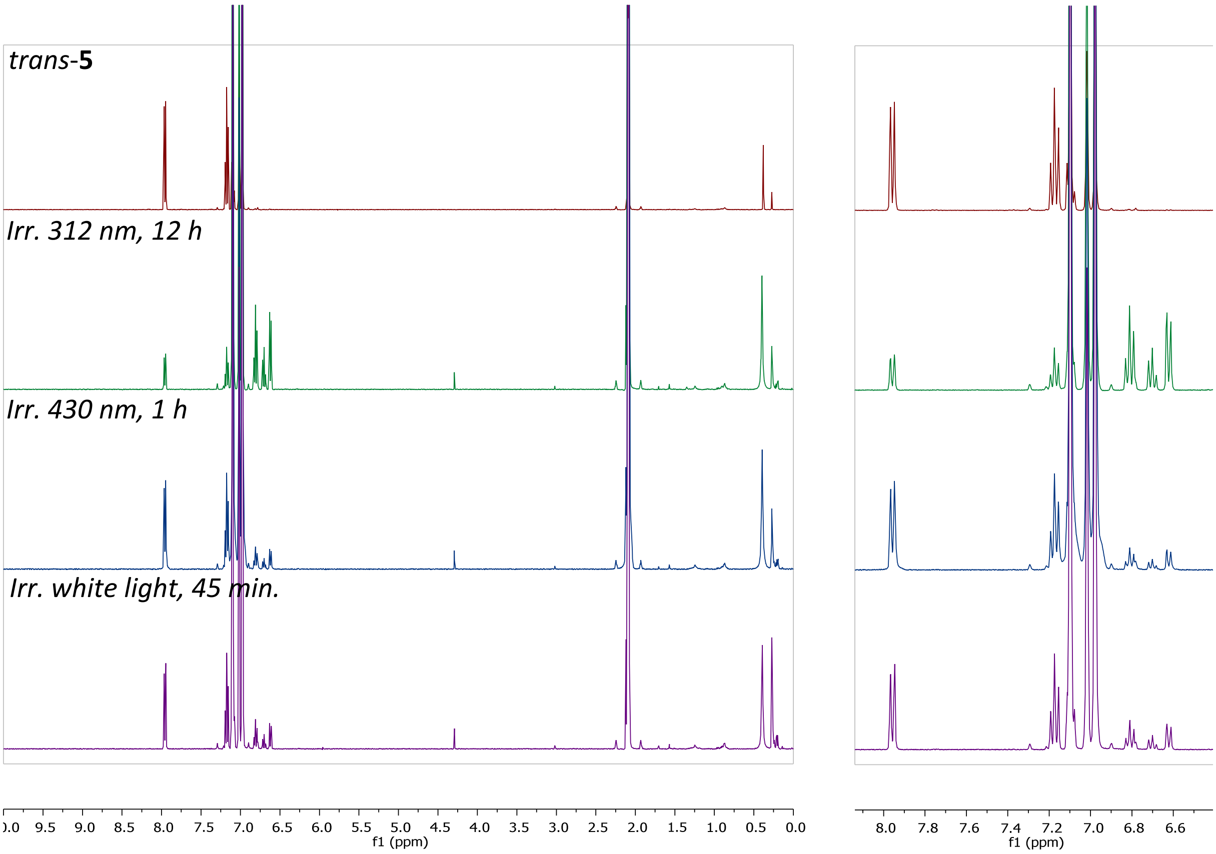

Supplementary Figure 35 | Determination of photostationary states (PSS) by ^{1}H-NMR for compound **5** in toluene-d_8; 2 mM: Irradiation of samples with indicated light sources and irradiation times. After each irradiation, NMR samples were diluted and checked by UV-vis spectroscopy whether the PSS was reached: λ = 312 nm for 12 h (Supplementary Table **1**, entry **1**; ENB-280C/FE,): 32:68 (*trans/cis*); λ = 430 nm for 1 h (Supplementary Table **1**, entry **6**; 607-4304H6,): 82:18 (*trans/cis*); white light for 45 min (Supplementary Table **1**, entry **12**; OSL1-EC,): 77:23 (*trans/cis*). ***Remark:*** The photostationary states for irradiation with λ = 312 nm and λ = 365 nm are comparable.

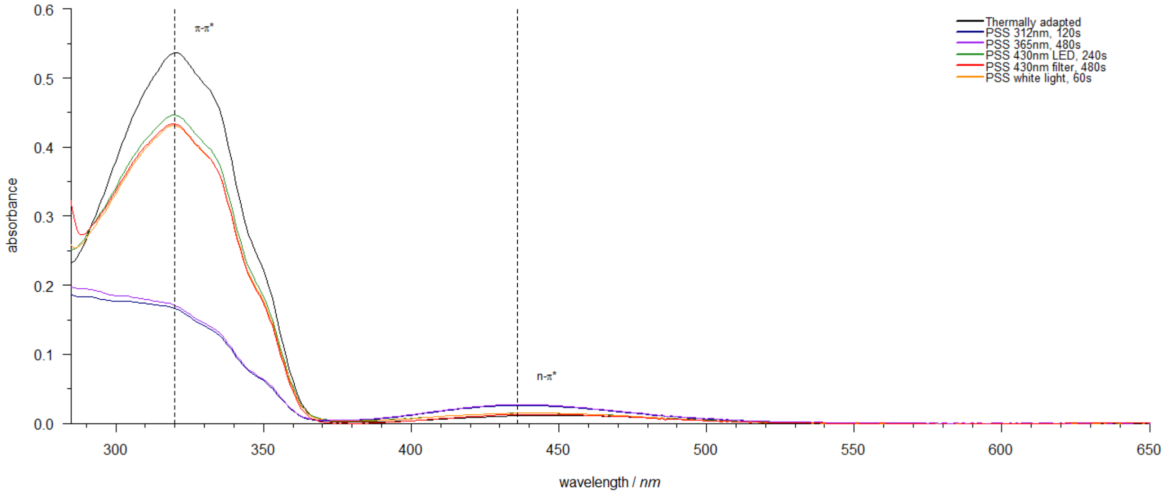

Supplementary Figure 36 | UV-vis absorption spectra illustrating the photostationary states (PSS) for compound **5** (~20 µM in toluene; room temperature). Irradiation times are indicated. Light sources used: λ = 312 nm (Supplementary Table **1**, entry **1**; ENB-280C/FE,); λ = 365 nm (br.) (Supplementary Table **1**, entry **2**; ENB-280C/FE,); λ = 430 nm (*Filter,* Supplementary Table **2**, entry **1**; 430FS10-50,); λ = 430 nm (*LED,* Supplementary Table **1**, entry **6**; 607-4304H6,) and white light (Supplementary Table **1**, entry **12**; OSL1-EC,).

Compound 6

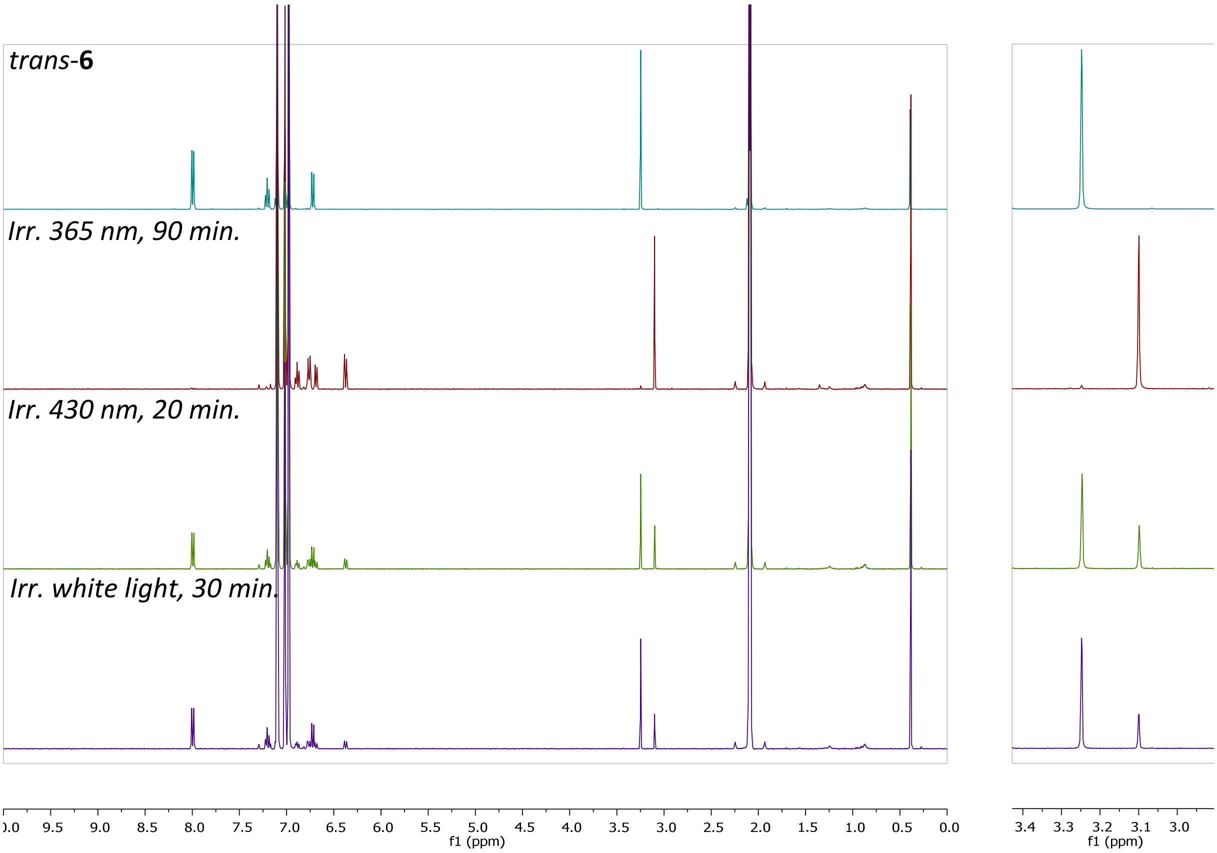

Supplementary Figure 37 | Determination of photostationary states (PSS) by ^{1}H-NMR for compound **6** in toluene-d_8; 2 mM: Irradiation of samples with indicated light sources and irradiation times. After each irradiation, NMR samples were diluted and checked by UV-vis spectroscopy whether the PSS was reached: λ = 365 nm (br.) for 90 min (Supplementary Table **1**, entry **2**; ENB-280C/FE,): < 3:97 (*trans/cis*); λ = 430 nm for 20 min (Supplementary Table **1**, entry **6**; 607-4304H6,): 69:31 (*trans/cis*); white light for 30 min (Supplementary Table **1**, entry **12**; OSL1-EC,): 76:24 (*trans/cis*).

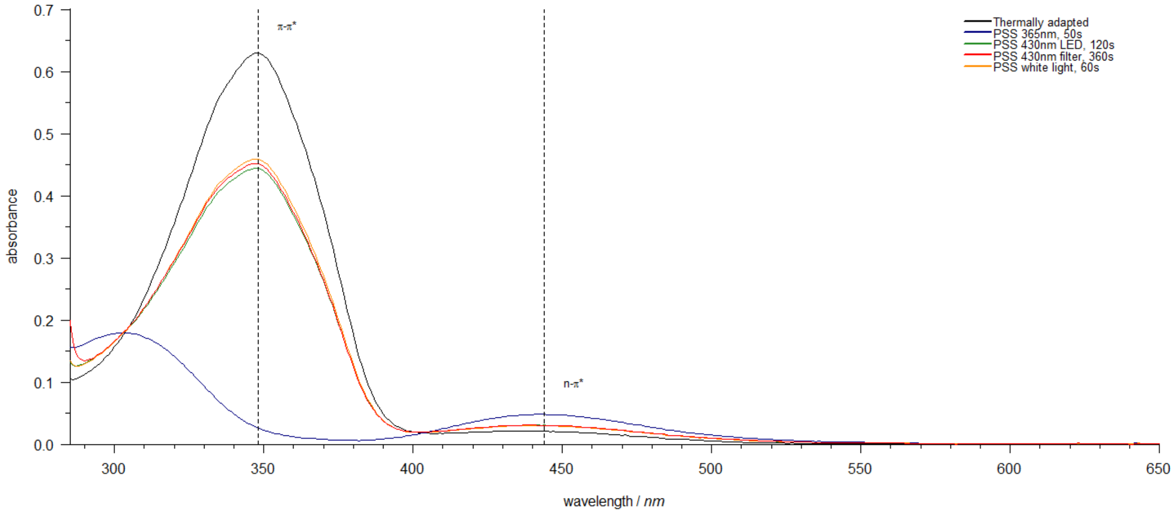

Supplementary Figure 38 | UV-vis absorption spectra illustrating the photostationary states (PSS) for compound **6** (~20 µM in toluene; room temperature). Irradiation times are indicated. Light sources used: λ = 365 nm (br.) (Supplementary Table **1**, entry **2**; ENB-280C/FE,); λ = 430 nm (*Filter,* Supplementary Table **2**, entry **1**; 430FS10-50,); λ = 430 nm (*LED,* Supplementary Table **1**, entry **6**; 607-4304H6,) and white light (Supplementary Table **1**, entry **12**; OSL1-EC,).

Photochemical Characterization of Two-Photoswitch Mixtures

Mixture of compound 1 + 3

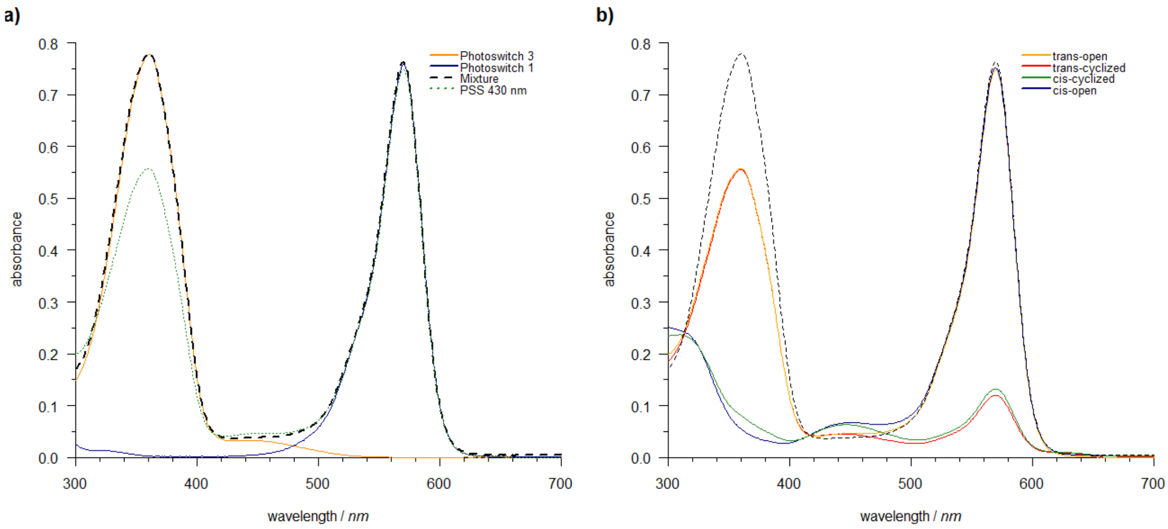

Supplementary Figure 39 | Reversible photochromism of the two-photoswitch system (compound **1** and **3; 1:** ~4 µM; **3:** ~20 µM; toluene; room temperature): (a) Absorption spectra of the individual photoswitches (*trans*-**3** and *open*-**1**) and their combination in a solution. (b) Absorption spectra of the four different functional states that can be achieved by irradiation in the mixture of **1** and **3** (*trans–open*; *trans–cyclized*; *cis–open* and *cis–cyclized*).

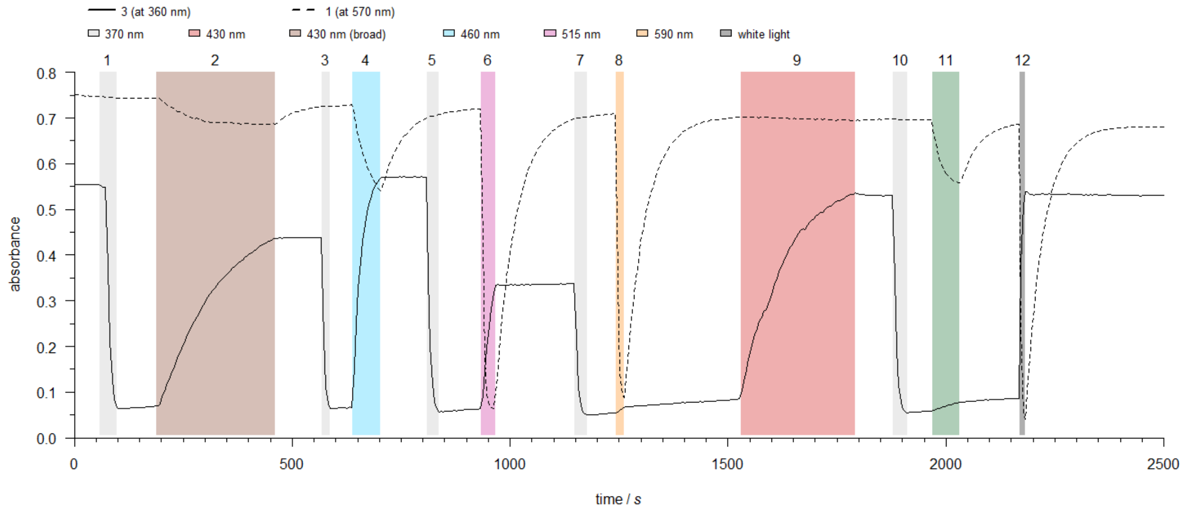

Supplementary Figure 40 | Effects of irradiation with different wavelengths on the selectivity of photoswitching of a mixture of compound **1** and **3** (**1:** ~4 µM; **3:** ~20 µM; toluene; room temperature) monitored at characteristic wavelengths for each photoswitch (λ = 360 nm for **3** and λ = 570 nm for **1**). Wavelengths of irradiations are indicated.

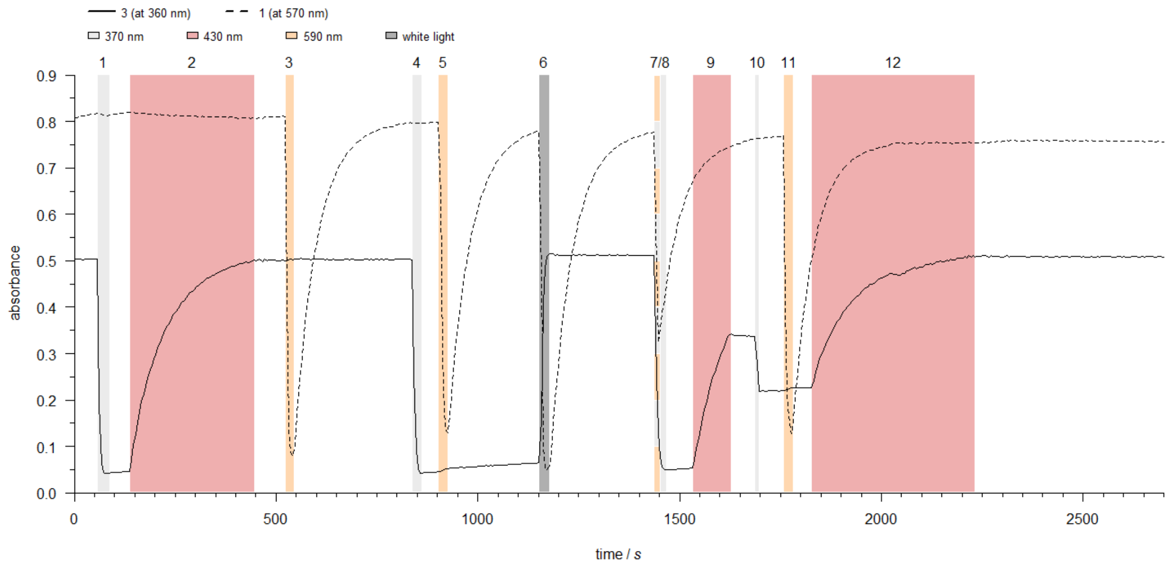

Supplementary Figure 41 | Orthogonal photoswitching of a mixture of compound **1** and **3** (**1:** ~4 µM; **3:** ~20 µM; toluene; room temperature) monitored at characteristic wavelengths for each photoswitch (λ = 360 nm for **3** and λ = 570 nm for **1**). Wavelengths of irradiations are indicated.

Proof for Photoswitching of 3 in a Mixture of 1 and 3 by ^{1}H-NMR

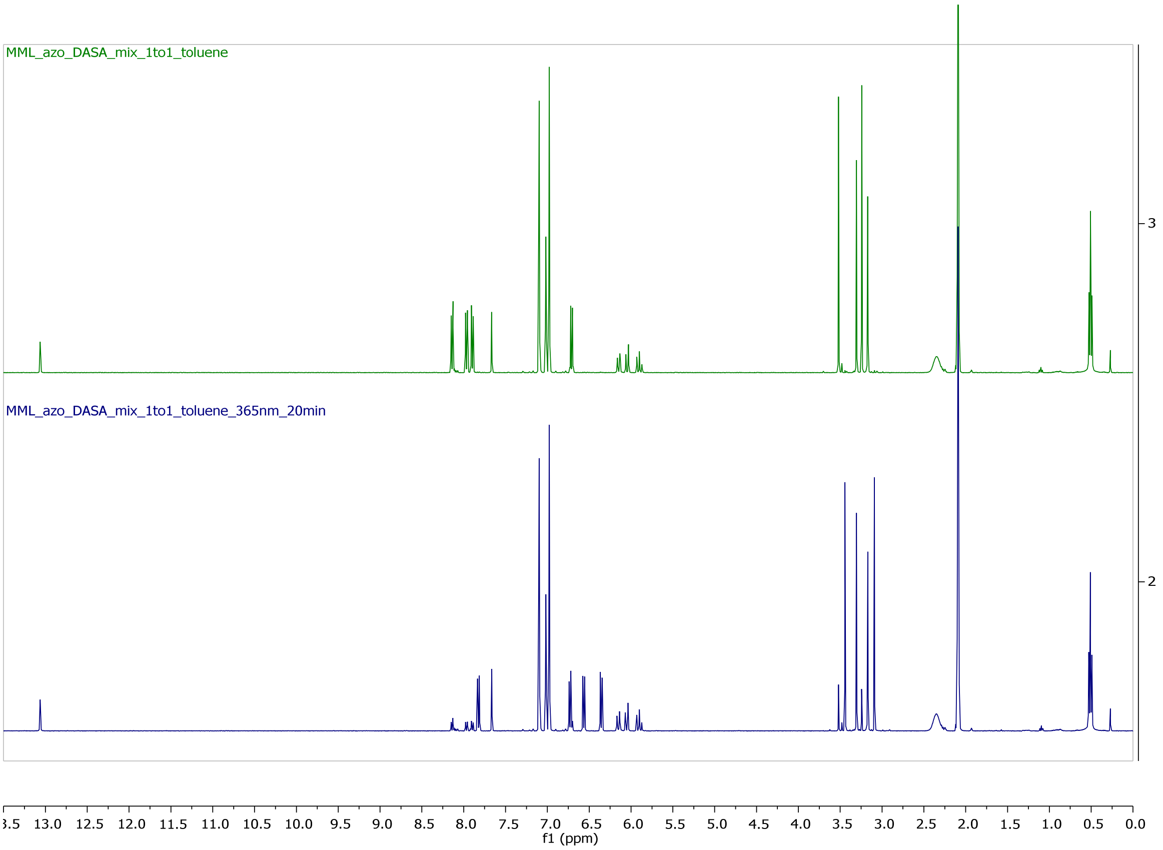

Supplementary Figure 42 | Photoswitching of the azobenzene in a 1:1 mixture of azobenzene **3** and DASA **1** (~4 mM; toluene-d_8, room temperature). (a) Non-irradiated mixture; (b) irradiation with λ = 365 nm (br.) (Supplementary Table **1**, entry **2**; ENB-280C/FE,) for 20 min..

Mixture of compound 1 + 4

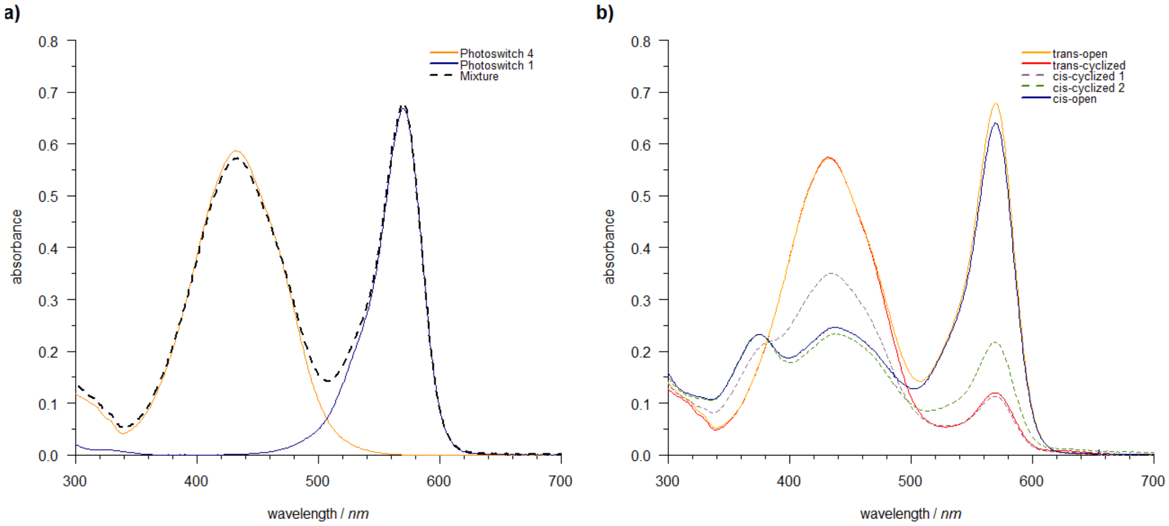

Supplementary Figure 43 | Reversible photochromism of the two-photoswitch system (compound **1** and **4; 1:** ~4 µM; **4:** ~20 µM; toluene; room temperature): (a) Absorption spectra of the individual photoswitches (*trans*-**4** and *open*-**1**) and their combination in a solution. (b) Absorption spectra of the four different functional states that can be achieved by irradiation in the mixture of **1** and **4** (*trans–open*; *trans–cyclized*; *cis–open* and *cis–cyclized*). ***Remark:*** In Fig. **43b**, there are two spectra depicted for the case *cis–cyclized*. The reason for this is exemplified in Fig. **44** with irradiations **9/10**: irradiation with λ = 590 nm also partially affects the azobenezene (**4**).

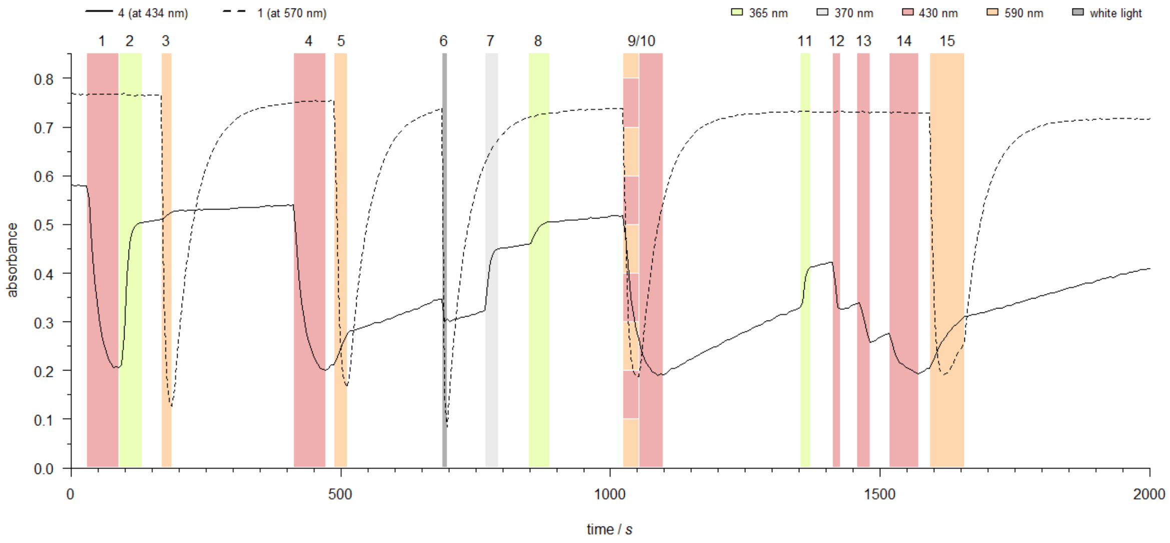

Supplementary Figure 44 | Orthogonal photoswitching of a mixture of compound **1** and **4** (**1:** ~4 µM; **4:** ~20 µM; toluene; room temperature) monitored at characteristic wavelengths for each photoswitch (λ = 434 nm for **4** and λ = 570 nm for **1**). Wavelengths of irradiations are indicated.

Mixture of compound 1 + 5

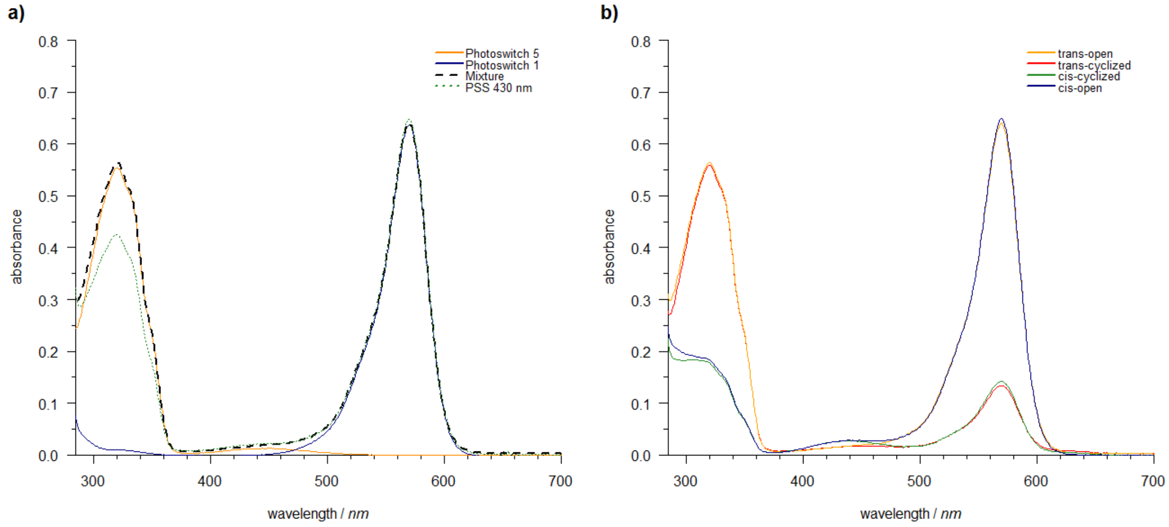

Supplementary Figure 45 | Reversible photochromism of the two-photoswitch system (compound **1** and **5; 1:** ~4 µM; **5:** ~20 µM; toluene; room temperature): (a) Absorption spectra of the individual photoswitches (*trans*-**5** and *open*-**1**) and their combination in a solution. (b) Absorption spectra of the four different functional states that can be achieved by irradiation in the mixture of **1** and **5** (*trans–open*; *trans–cyclized*; *cis–open* and *cis–cyclized*).

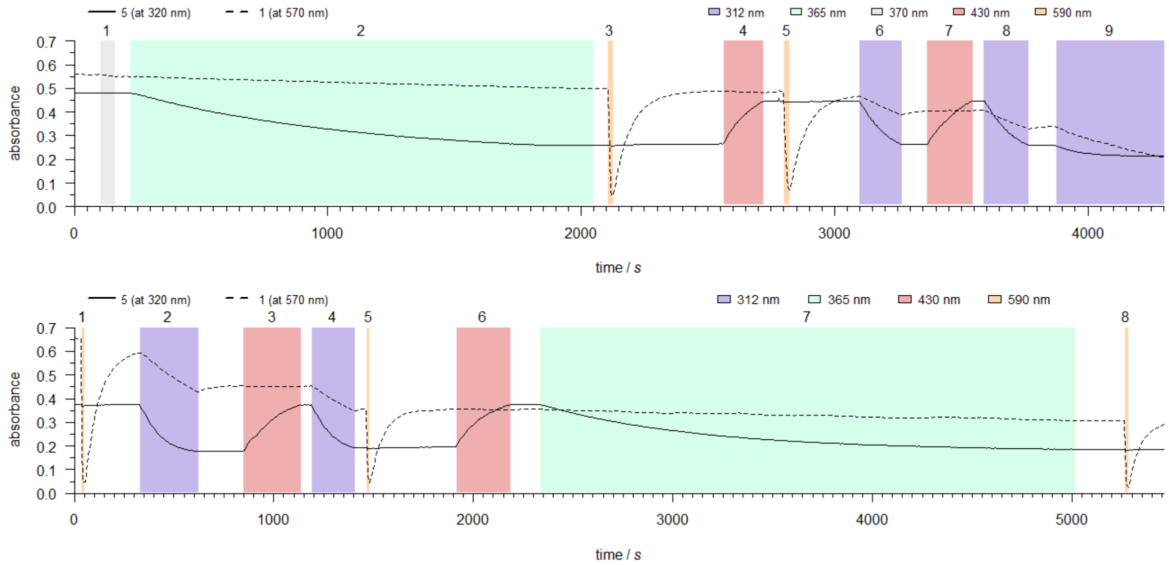

Supplementary Figure 46 | Orthogonal photoswitching of a mixture of compound **1** and **5** (**1:** ~4 µM; **5:** ~20 µM; toluene; room temperature) monitored at characteristic wavelengths for each photoswitch (λ = 320 nm for **5** and λ = 570 nm for **1**). Two plots (a) and (b) with different order of irradiation. Wavelengths of irradiations are indicated. ***Remark:*** Irradiation with λ = 312 nm switches compound **5** (λ_{max} = 320 nm), but also results in slow degradation of photoswitch **1** (***irradiations 6, 8 and 9***). This is in accordance to earlier reports.[4]

Mixture of compound 1 + 6

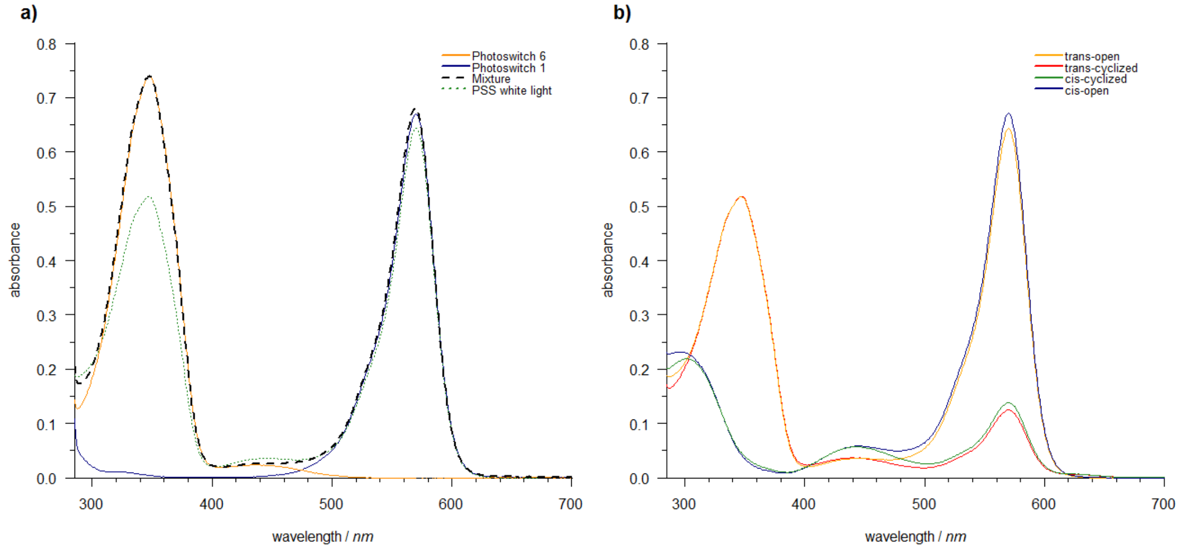

Supplementary Figure 47 | Reversible photochromism of the two-photoswitch system (compound **1** and **6; 1:** ~4 µM; **6:** ~15 µM; toluene; room temperature): (a) Absorption spectra of the individual photoswitches (*trans*-**6** and *open*-**1**) and their combination in a solution. (b) Absorption spectra of the four different functional states that can be achieved by irradiation in the mixture of **1** and **6** (*trans–open*; *trans–cyclized*; *cis–open* and *cis–cyclized*).

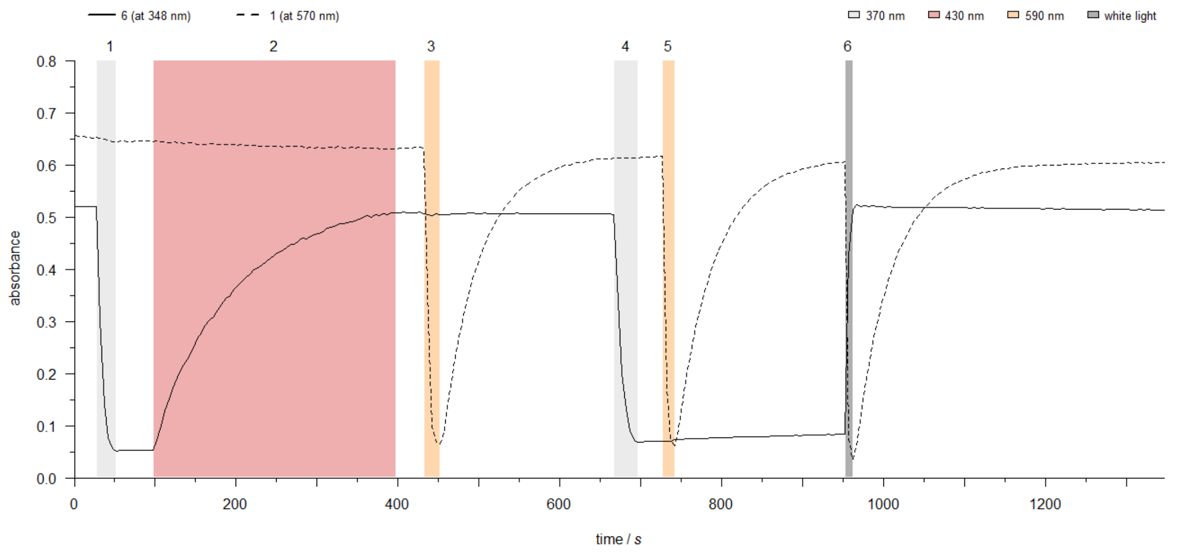

Supplementary Figure 48 | Orthogonal photoswitching of a mixture of compound **1** and **6** (**1:** ~4 µM; **6:** ~15 µM; toluene; room temperature) monitored at characteristic wavelengths for each photoswitch (λ = 348 nm for **6** and λ = 570 nm for **1**). Wavelengths of irradiations are indicated.

Mixture of compound 2 + 6

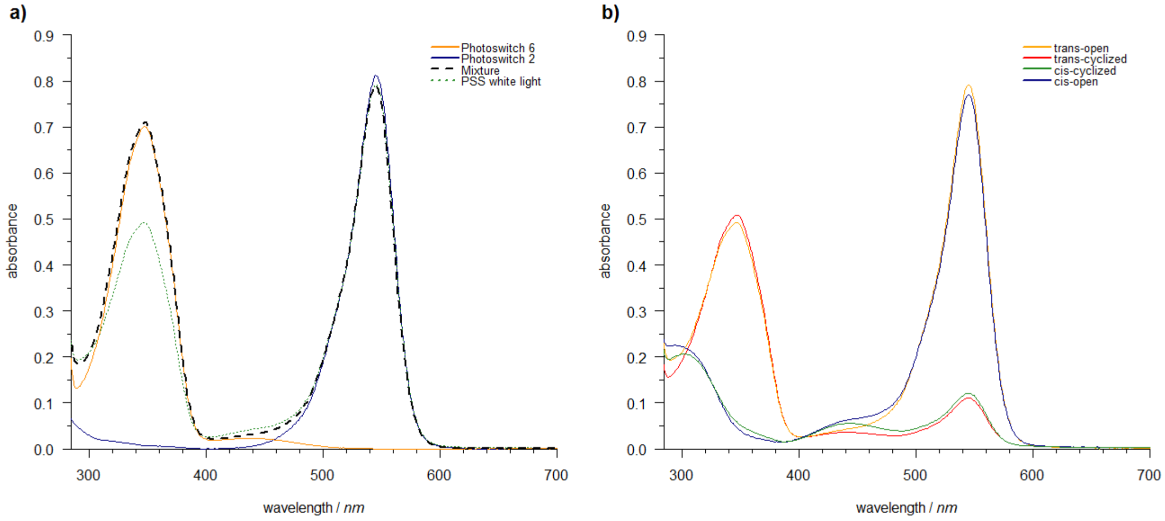

Supplementary Figure 49 | Reversible photochromism of the two-photoswitch system (compound **2** and **6; 2:** ~4 µM; **6:** ~15 µM; toluene; room temperature): (a) Absorption spectra of the individual photoswitches (*trans*-**6** and *open*-**2**) and their combination in a solution. (b) Absorption spectra of the four different functional states that can be achieved by irradiation in the mixture of **2** and **6** (*trans–open*; *trans–cyclized*; *cis–open* and *cis–cyclized*).

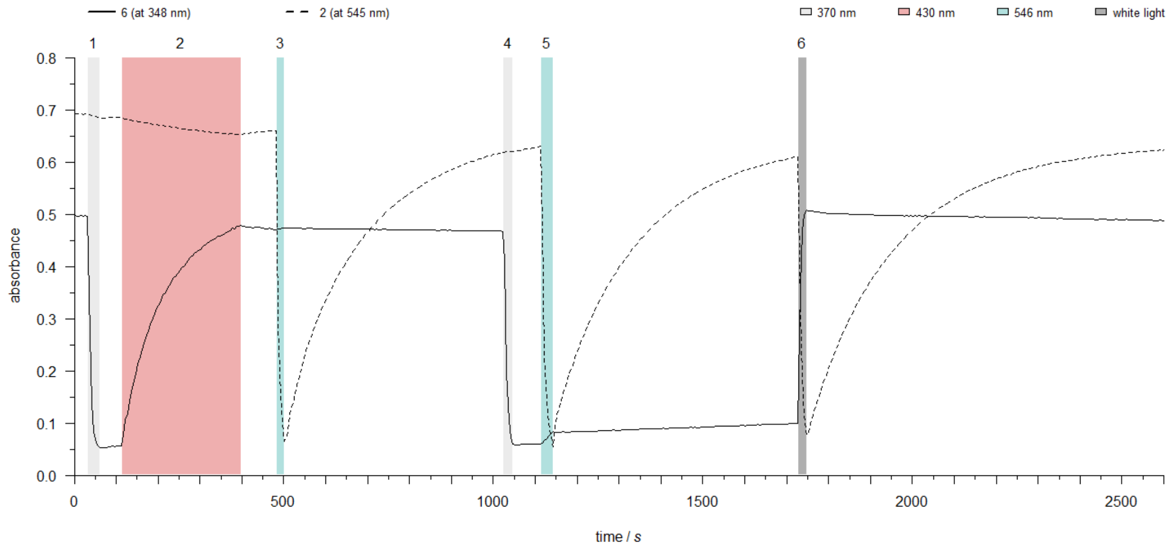

Supplementary Figure 50 | Orthogonal photoswitching of a mixture of compound **2** and **6** (**2:** ~4 µM; **6:** ~15 µM; toluene; room temperature) monitored at characteristic wavelengths for each photoswitch (λ = 348 nm for **6** and λ = 545 nm for **2**). Wavelengths of irradiations are indicated.

Photoswitching at Higher Concentrations

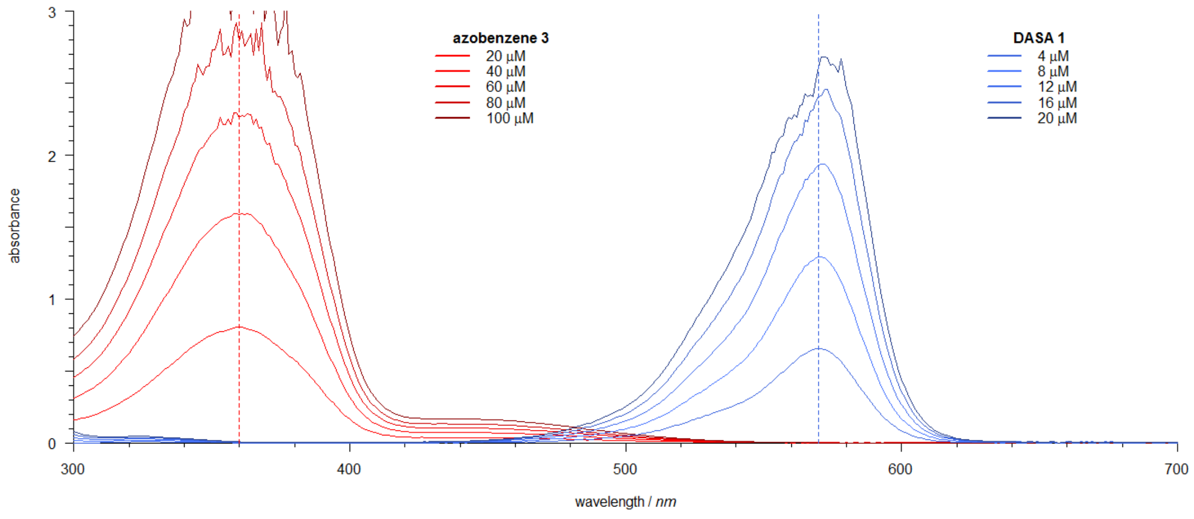

Supplementary Figure 51 | Overlay and comparison of different concentrations of the single photoswitches **1** or **3** and their absorption spectra.

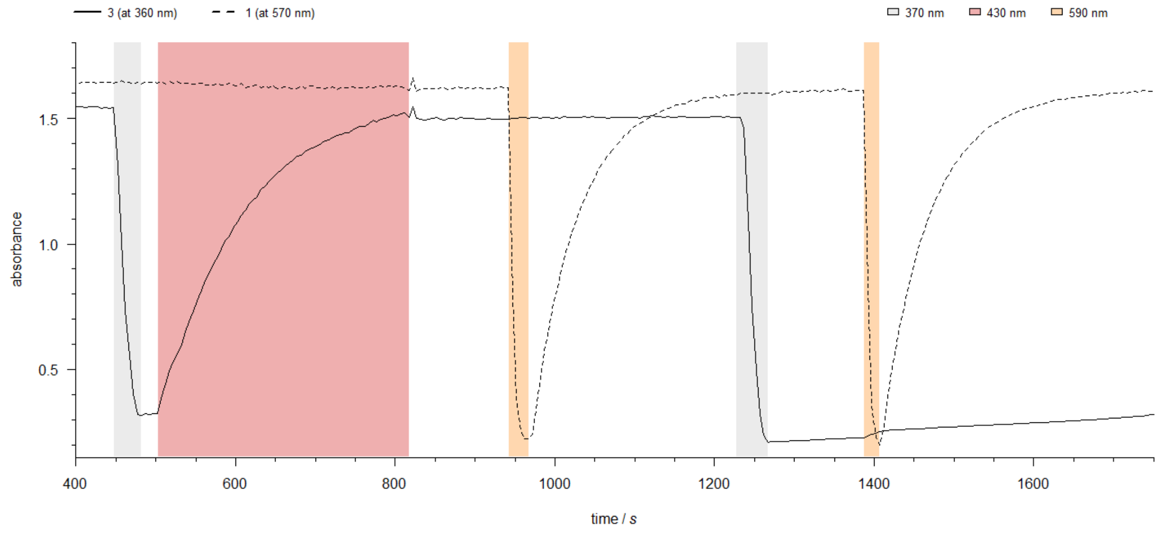

Supplementary Figure 52 | Orthogonal photoswitching of a concentrated mixture of compound **1** and **3** (**1:** ~12 µM; **3:** ~60 µM; toluene; room temperature; 10 mm quartz cuvette) monitored at characteristic wavelengths for each photoswitch (λ = 360 nm for **3** and λ = 570 nm for **1**), in order to assess whether the orthogonality of the intermolecular photoswitching is dependent on the concentration used.

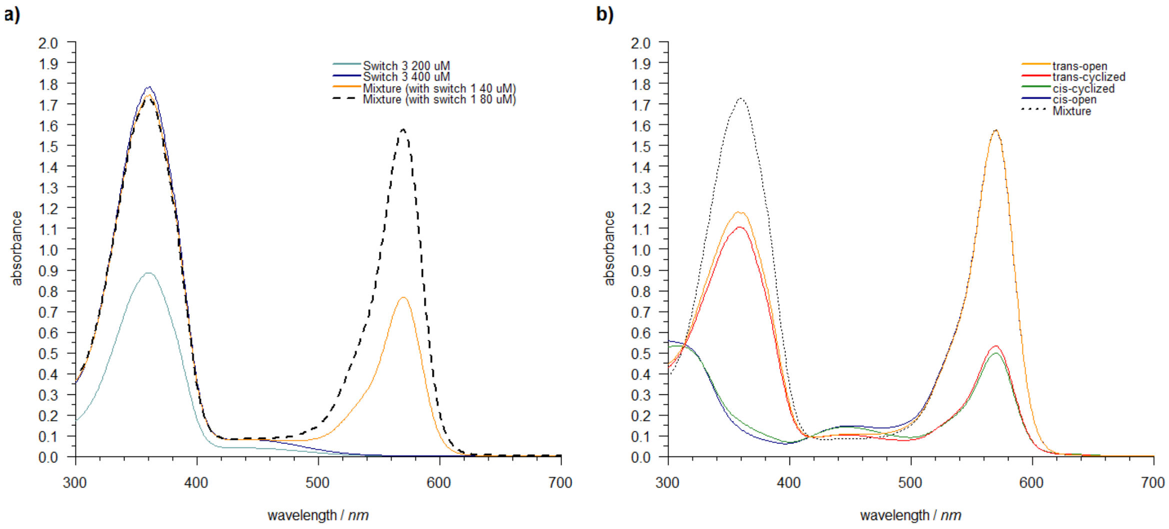

Supplementary Figure 53 | a) Absorption spectra of different concentrations of single photoswitches and mixtures thereof. b) Absorption spectra of the four different functional states that can be achieved by irradiation in the mixture of **1** and **3** (**1:** ~80 µM; **3:** ~400 µM; toluene; room temperature; 1.0 mm quartz cuvette).

Characterization of Photoswitching in an Intramolecular Combination of Photoswitches

Photoswitches 1 and S6 and their intramolecular analogue 7

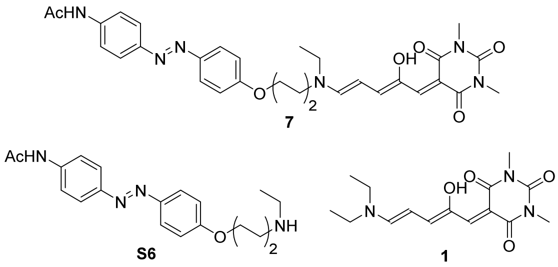

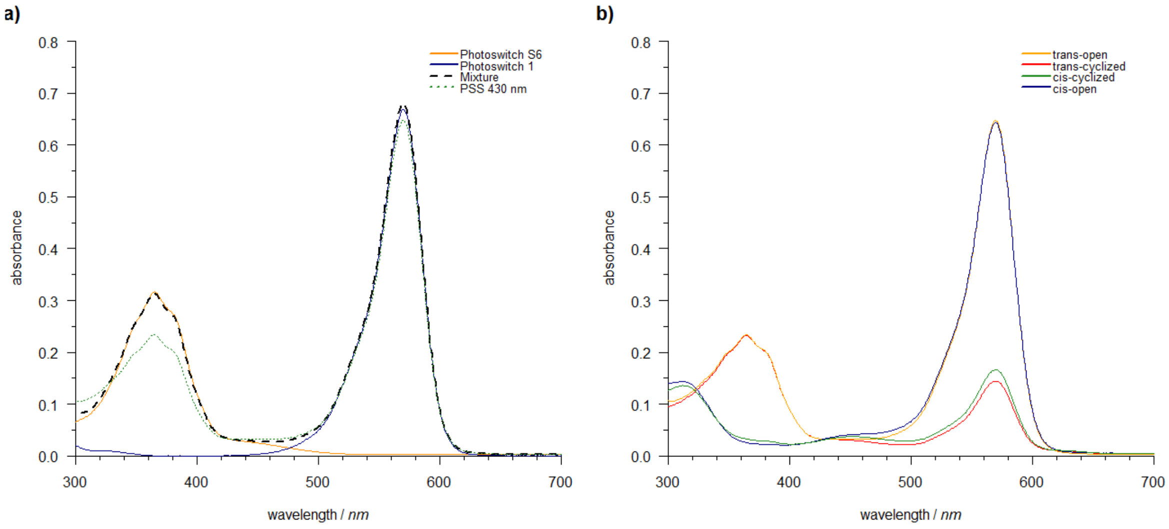

Supplementary Figure 54 | Reversible photochromism of the two-photoswitch system (compound **1** and **S6; 1:** ~4 µM; **S6:** ~10 µM; toluene; room temperature): (a) Absorption spectra of the individual photoswitches (*trans*-**S6** and *open*-**1**) and their combination in a solution. (b) Absorption spectra of the four different functional states that can be achieved by irradiation in the mixture of compounds **1** and **S6** (*trans–open*; *trans–cyclized*; *cis–open* and *cis–cyclized*).

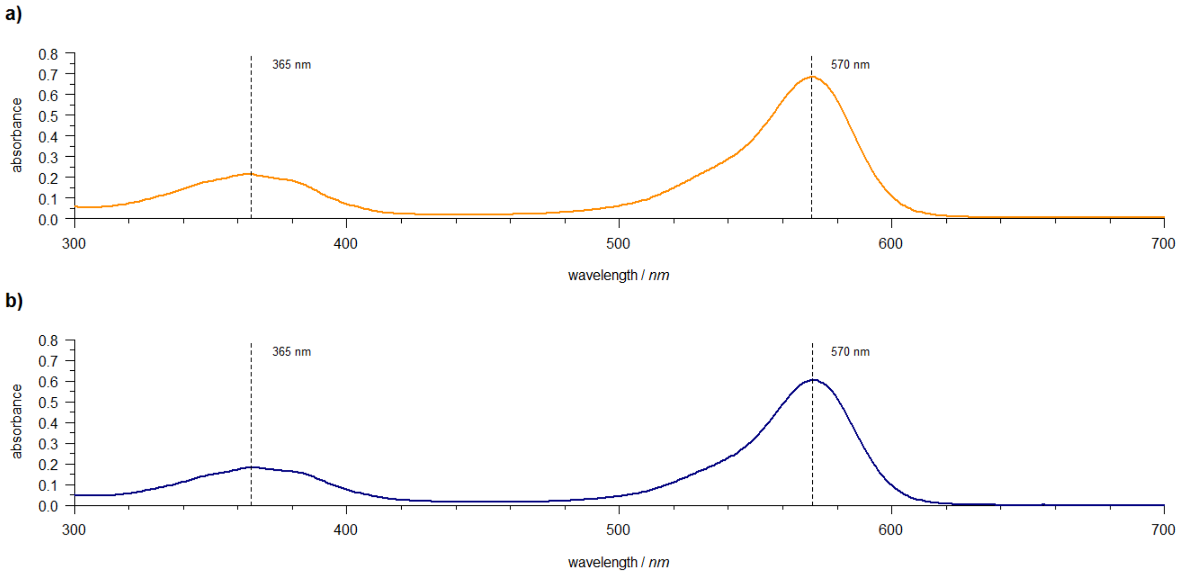

Supplementary Figure 55 | Absorption spectra of (a) compound **7** and (b) compound **8** (~4 µM; toluene; room temperature).

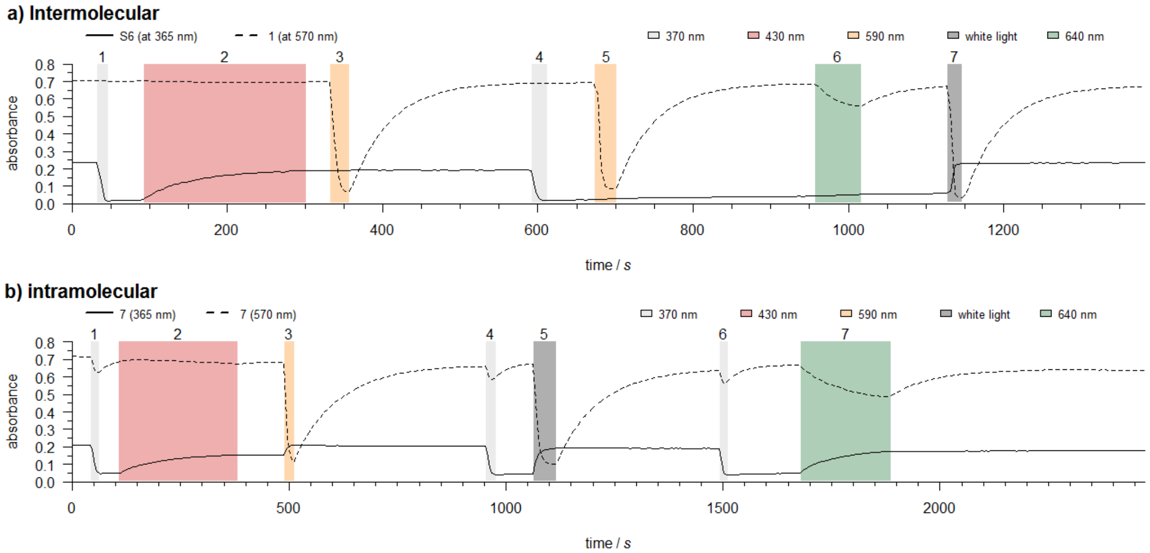

Supplementary Figure 56 | Photoswitching in the inter- (a) and intramolecular (b) case (toluene; room temperature): a) Orthogonal photoswitching of a mixture of compound **1** and **S6** (**1:** ~4 µM; **S6:** ~10 µM). b) Photoswitching of compound **7** (~4 µM). Monitored characteristic wavelengths for each photoswitch and wavelengths of irradiations are indicated.

Photoswitches 1 and S8 and their intramolecular analogue 8

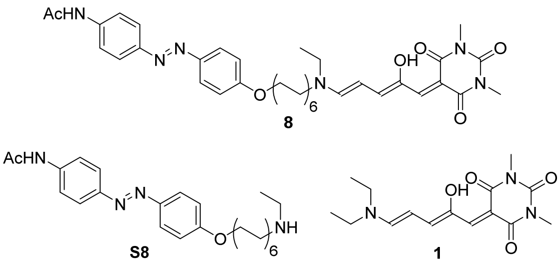

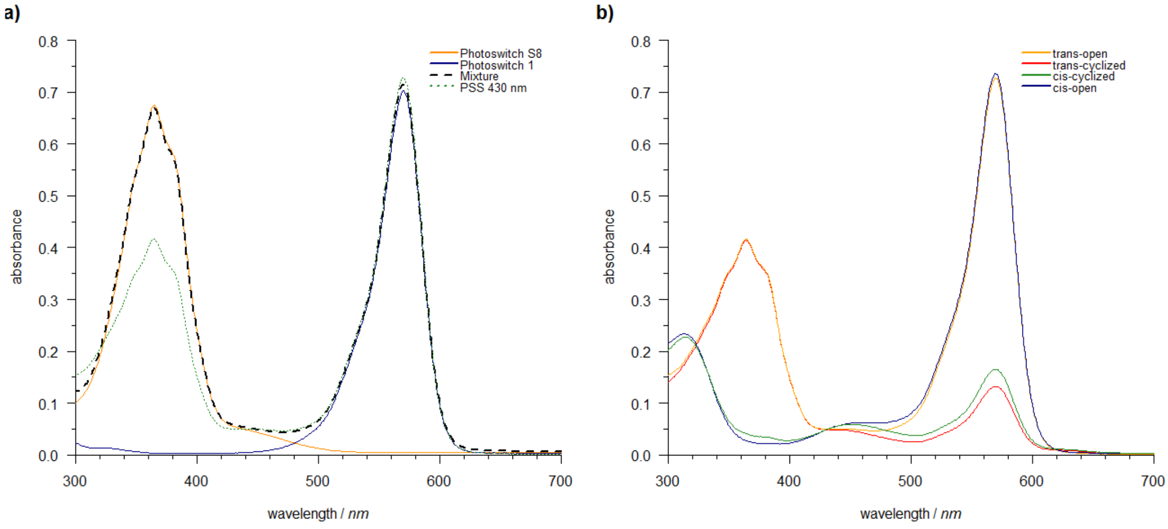

Supplementary Figure 57 | Reversible photochromism of the two-photoswitch system (compound **1** and **S8; 1:** ~4 µM; **S8:** ~20 µM; toluene; room temperature): (a) Absorption spectra of the individual photoswitches (*trans*-**S8** and *open*-**1**) and their combination in a solution. (b) Absorption spectra of the four different functional states that can be achieved by irradiation in the mixture of **1** and **S8** (*trans–open*; *trans–cyclized*; *cis–open* and *cis–cyclized*).

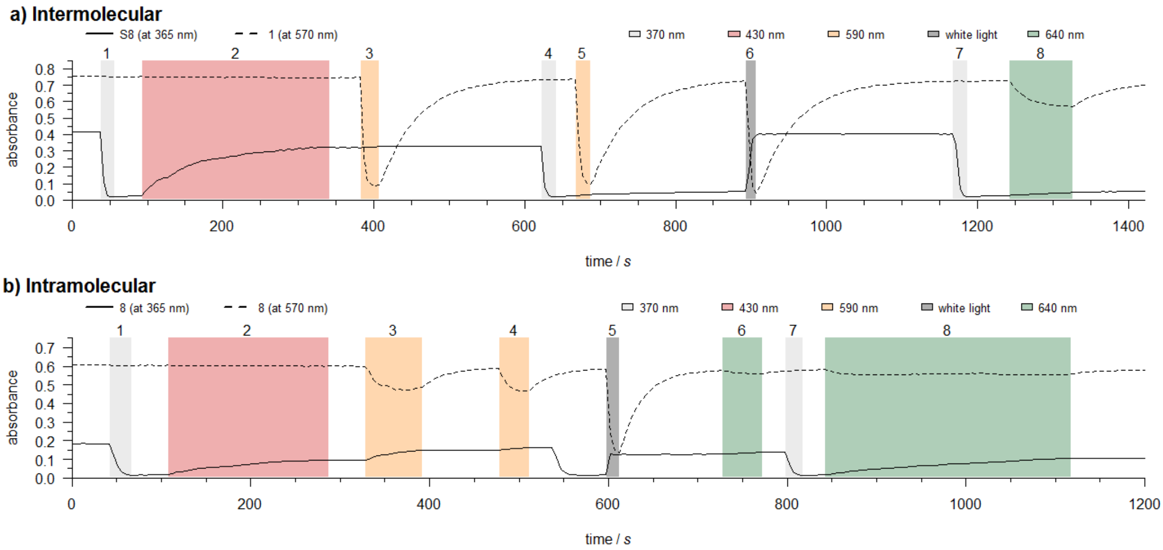

Supplementary Figure 58 | Photoswitching in the inter- (a) and intramolecular (b) case (toluene; room temperature): a) Orthogonal photoswitching of a mixture of compound **1** and **S8** (**1:** ~4 µM; **S8:** ~20 µM). b) Photoswitching of compound **8** (~4 µM). Monitored characteristic wavelengths for each photoswitch and wavelengths of irradiations are indicated.

Extraction Experiments

Study of a mixture of photoswitch 3 and 9

a)

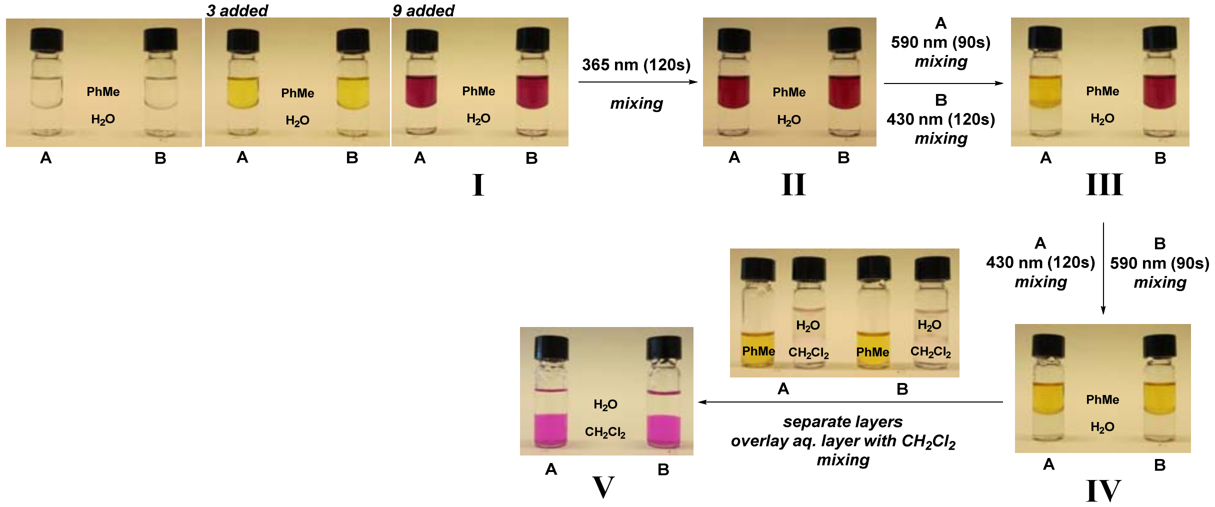

b)

A) *365 nm -> 590 nm -> 430 nm -> back-extraction*

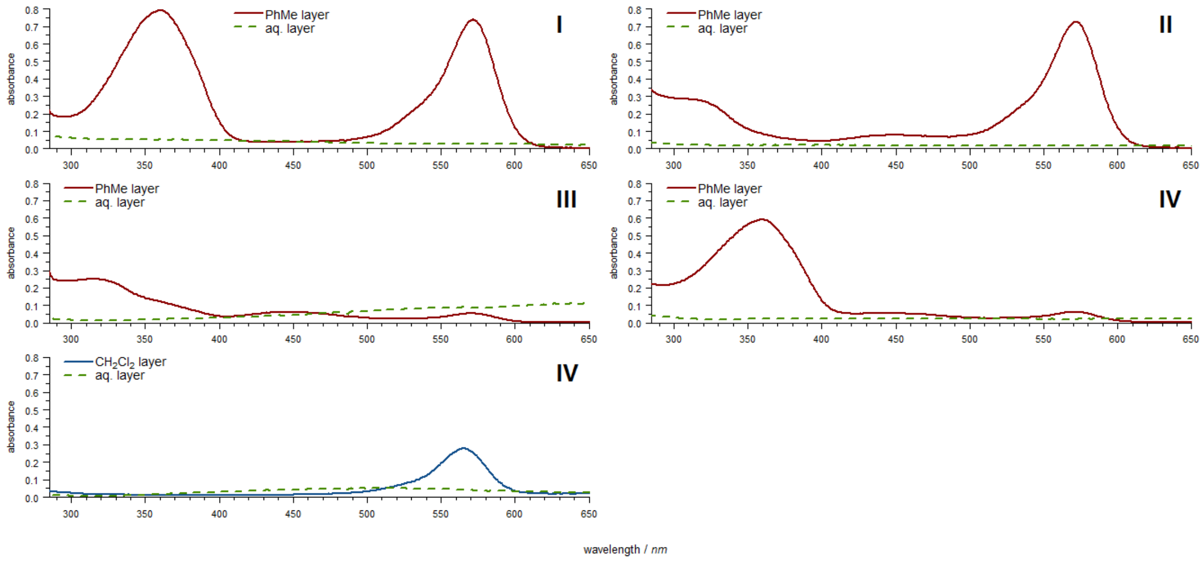

B) *365 nm -> 430 nm -> 590 nm -> back-extraction*

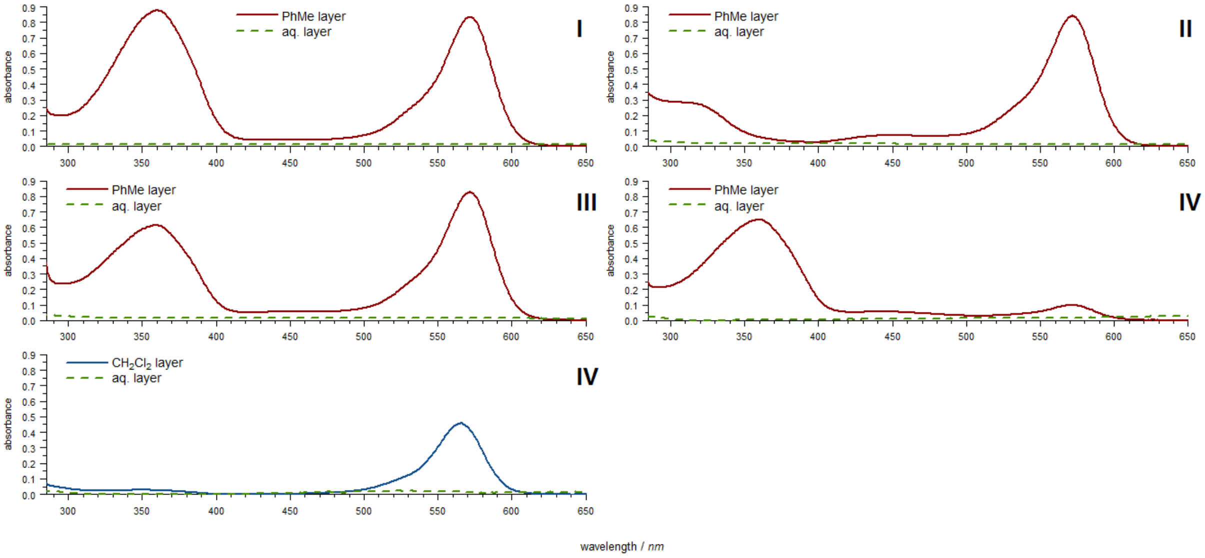

c)

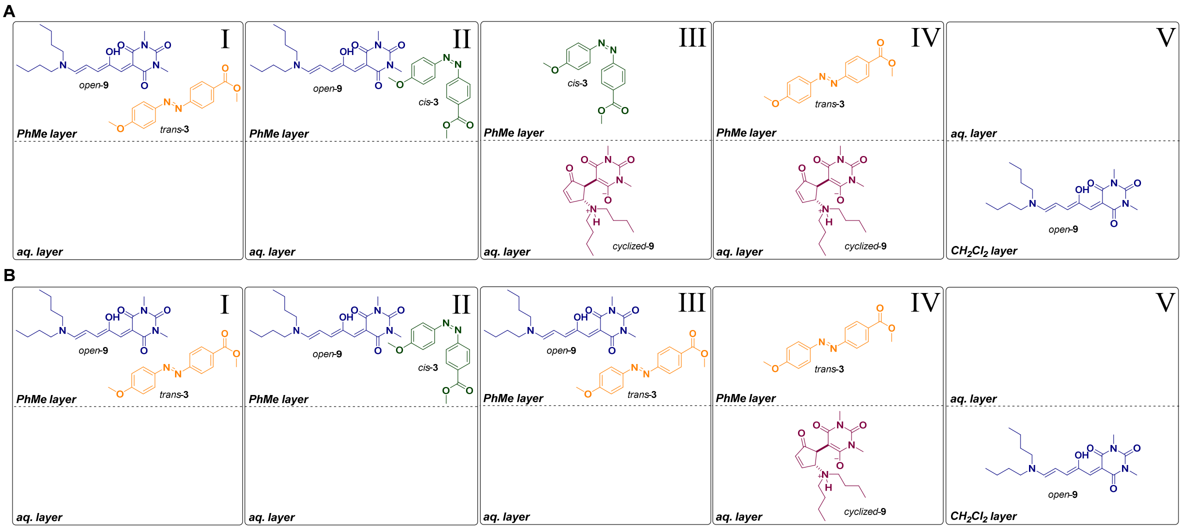

Supplementary Figure 59 | Dynamic phase transfer of a mixture of azobenzene **3** and DASA **9** in toluene/water. Two samples **A** and **B** were subjected to different irradiation sequences (**A**: 365 nm -> 590 nm -> 430 nm -> back-extraction; **B**: 365 nm -> 430 nm -> 590 nm -> back-extraction) and the process was monitored by color (a) and UV-vis spectroscopy (b) and is conceptualized in (c). a) The switching process can be monitored by phase-color (**3**: 500 µM; **9**: 100 µM; toluene/water; room temperature). b) UV-vis absorption spectra of both the organic and aqueous layer (at stages *i* to *v*) (**3**: 20 µM; **9**: 4 µM; toluene/water; room temperature). c) The presence of compounds **3** and **9** in the different phases is schematically depicted.

Study of compound 8

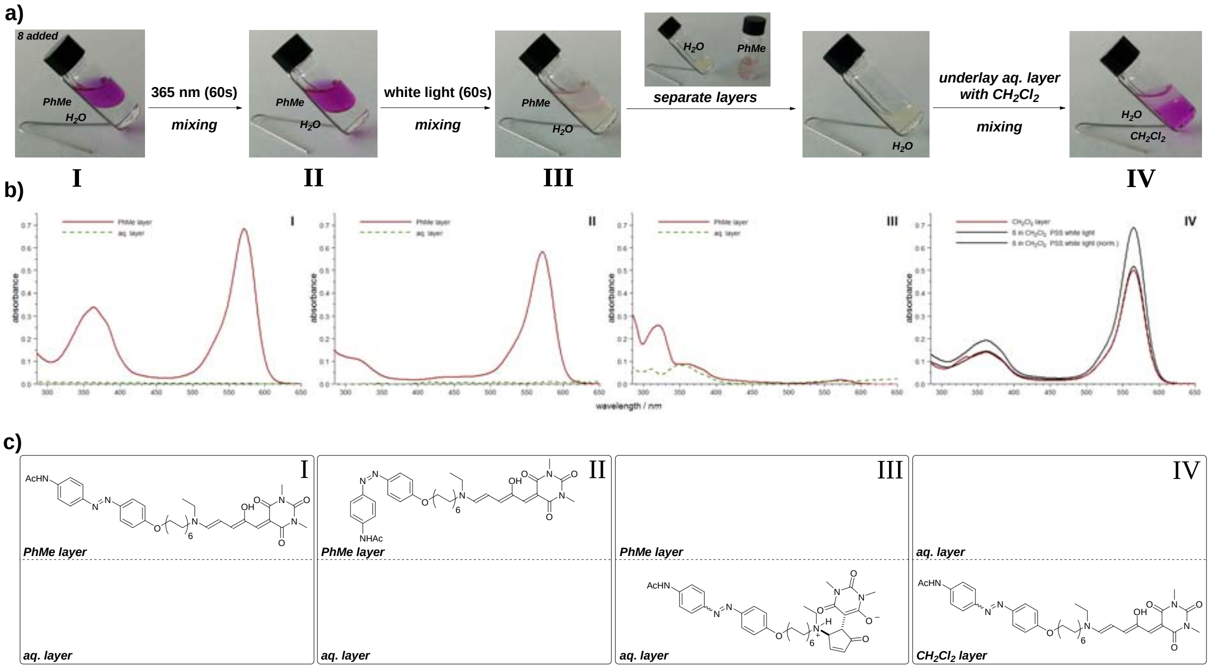

Supplementary Figure 60 | Dynamic phase transfer of compound **8** in a toluene/sat. aq. $NaHCO_3$ (pH = 9) mixture. The independent switching and phase-transfer events can be monitored by phase-color (a) and UV-vis spectroscopy (b). *State I*: Initial conditions, compound **8** resides in the organic phase (purple color). *State II*: The azobenzene part of compound **8** was switched by irradiation at λ = 365 nm. The DASA part remains untouched. *State III*: Irradiation with white light results in the cyclization of the DASA moiety, decoloration and phase-transfer of compound **8**. *State IV*: Separating the layers and back-extraction of the aqueous layer with dichloromethane results in back-transfer of compound **8** to the organic phase, as can be observed by the recoloration of this phase (purple color). The presence of compounds in the different phases is schematically depicted (c).

Binding Studies

Photoswitching of the azobenzene moiety

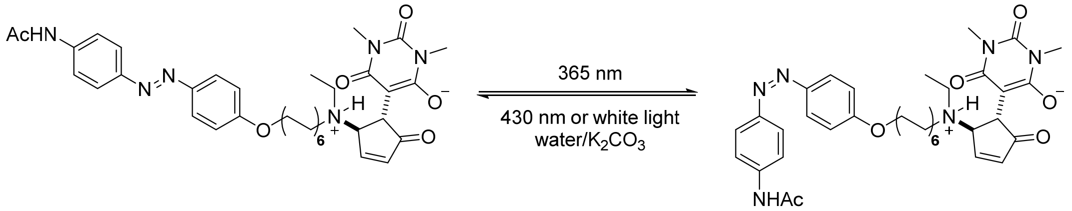

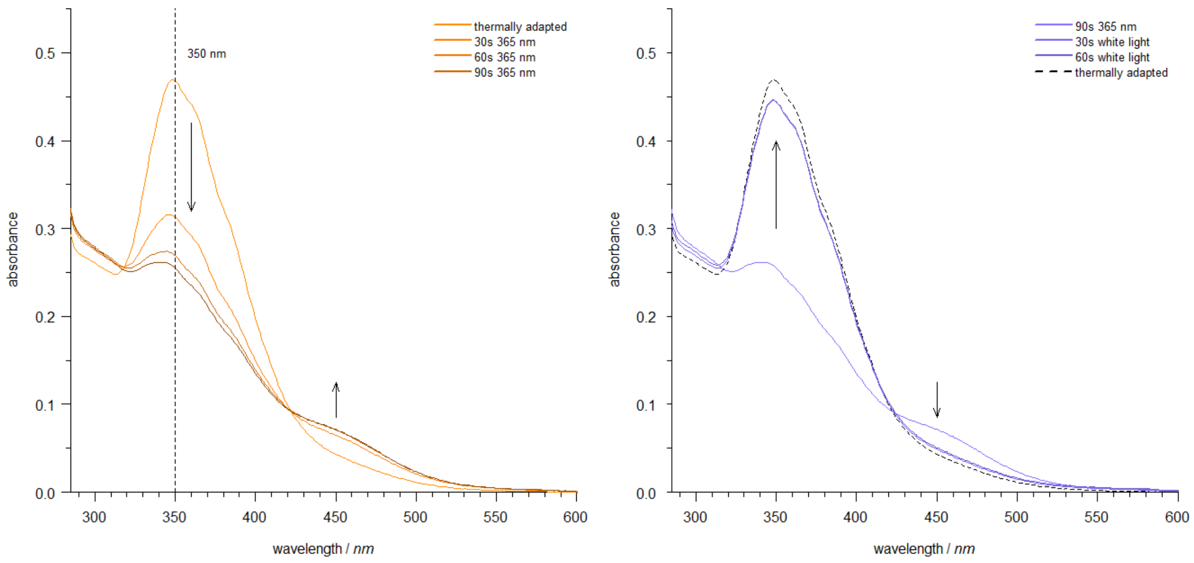

Supplementary Figure 61 | Absorption spectra for the photoisomerization of compound ***cyclized*-8** (λ_{max} = 350 nm; ~11 µM in water pH ≥ 9; room temperature): a) switching *trans* to *cis* with λ = 365 nm (br.) (Supplementary Table **1**, entry **2**; ENB-280C/FE,) and b) *cis* to *trans*, with white light (Supplementary Table **1**, entry **12**; OSL1-EC,). Irradiation times are indicated.

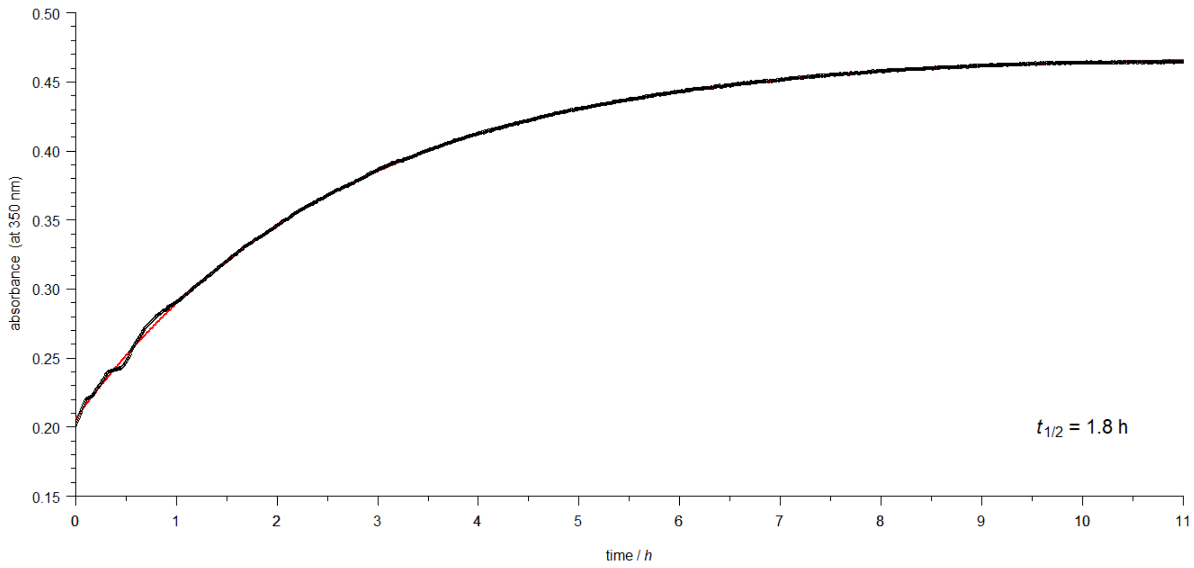

Supplementary Figure 62 | Determination of thermal stability of the *cis*-azobenzene moiety of compound ***cyclized*-8** (~11 µM): Points correspond to measured data (observed at λ_{max} = 350 nm,

water pH ≥ 9; room temperature); line represents the fitting with single exponential process. The half-life was determined to: $t_{1/2}$ = 1.8 h.

UV-vis Studies on Host-Guest Binding[1,2]

Titrations

Thermally adapted ***cyclized*-8**

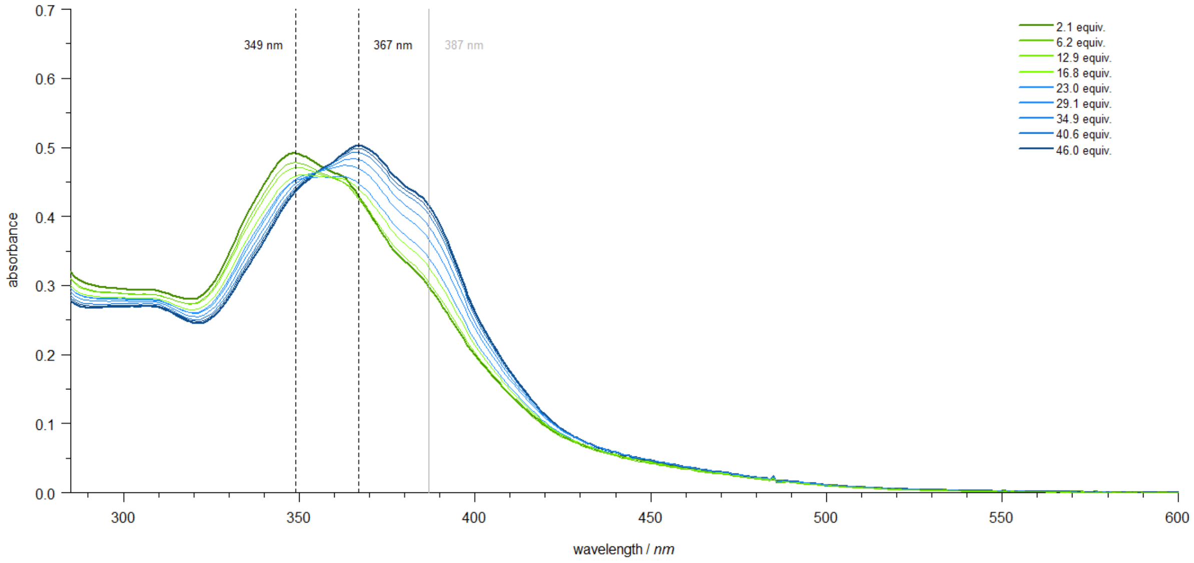

Supplementary Figure 63 | Absorption spectra for the binding of compound ***cyclized*-8** (λ_{max} = 350 nm; 11 µM in water pH ≥ 9; room temperature) with α-CD: a) before and b) after addition of solution **B**.

PSS 365 nm ***cyclized*-8**

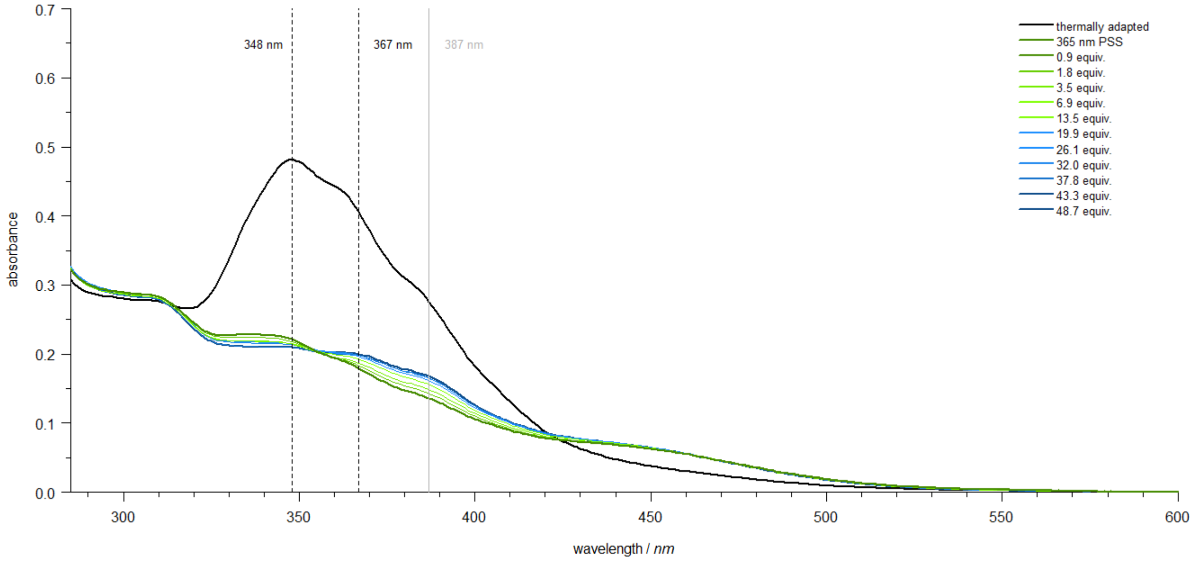

Supplementary Figure 64 | Absorption spectra for the photoisomerization and binding of compound ***cyclized*-8** (λ_{max} = 350 nm; 11 µM in water pH ≥ 9; room temperature) with α-CD: a) before and b) after addition of solution **B**. Switching *trans* to *cis* with λ = 365 nm (br.) (Supplementary Table **1**, entry **2**; ENB-280C/FE,). The spectra for the photostationary states are given.

Control experiment 1

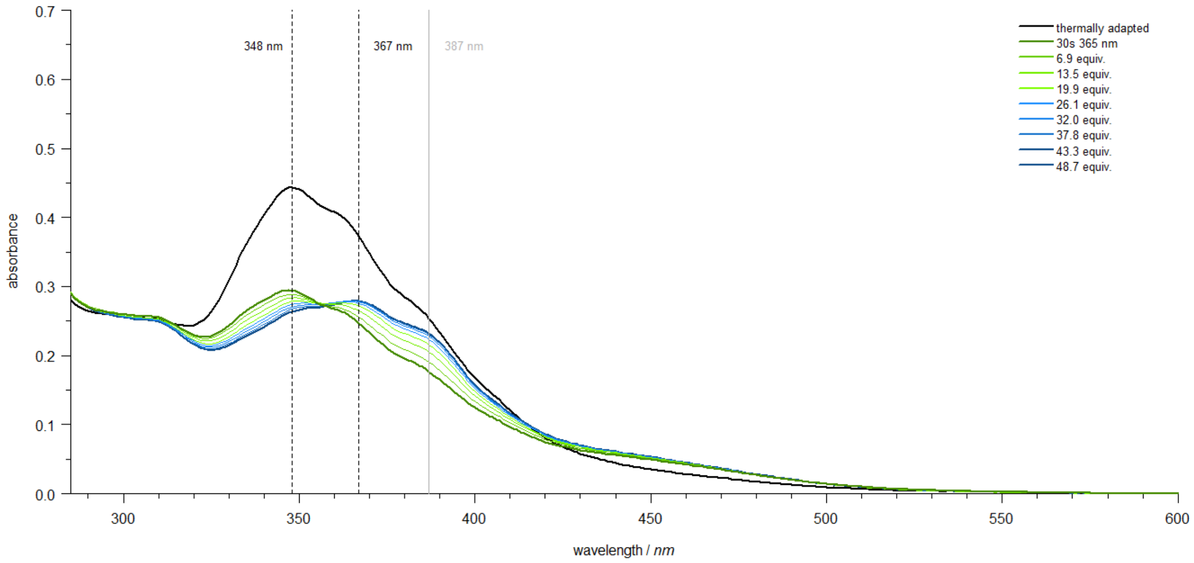

Supplementary Figure 65 | Absorption spectra for the photoisomerization and binding of compound ***cyclized*-8** (λ_{max} = 350 nm; 11 µM in water pH ≥ 9; room temperature) with α-CD: a) before and b) after addition of solution **B**. Switching *trans* to *cis* with λ = 365 nm (br.) (Supplementary Table **1**, entry **2**; ENB-280C/FE,).

Control experiment 2

20s 365 nm ***cyclized*-8**

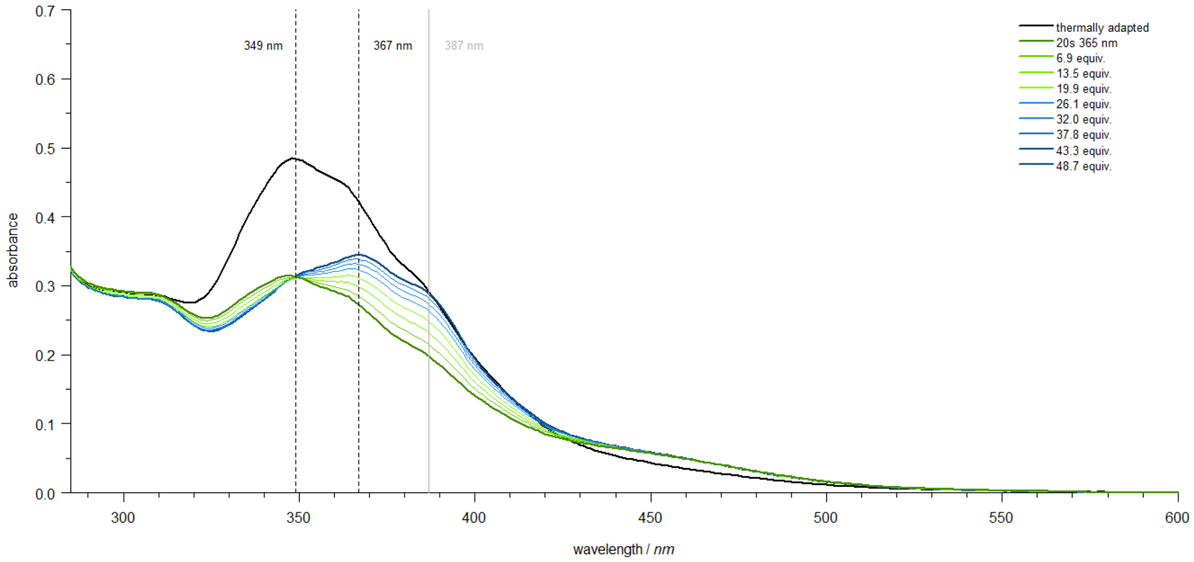

Supplementary Figure 66 | Absorption spectra for the photoisomerization and binding of compound ***cyclized*-8** (λ_{max} = 350 nm; 11 µM in water pH ≥ 9; room temperature) with α-CD: a) before and b) after addition of non-irradiated solution **B**. Switching *trans* to *cis* with λ = 365 nm (br.) (Supplementary Table **1**, entry **2**; ENB-280C/FE,).

PSS 365 nm ***cyclized*-8**

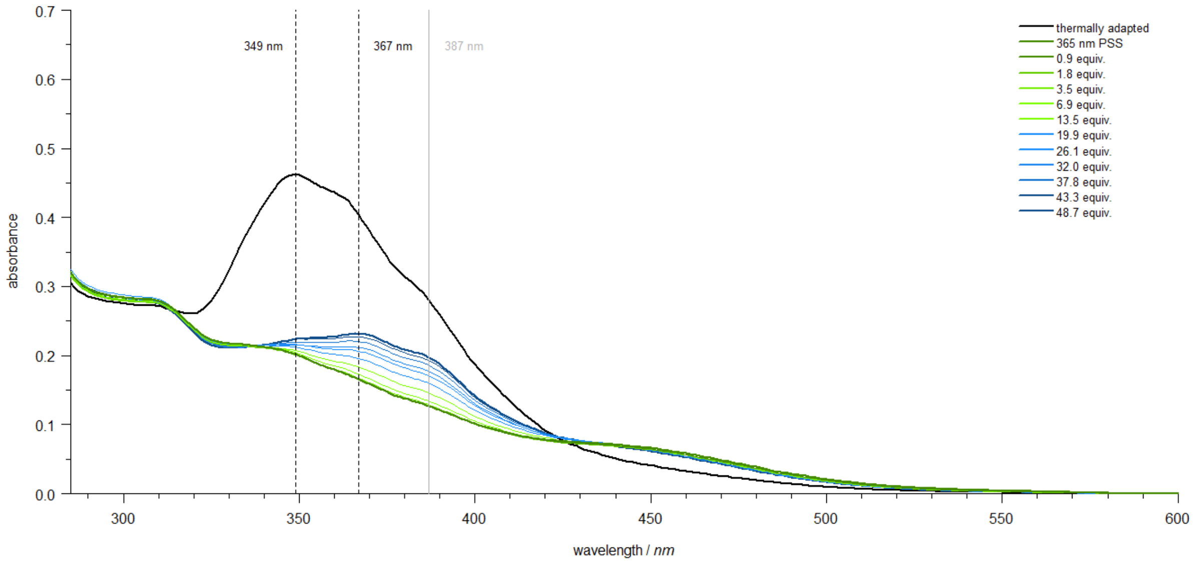

Supplementary Figure 67 | Absorption spectra for the photoisomerization and binding of compound ***cyclized*-8** (λ_{max} = 350 nm; 11 µM in water pH ≥ 9; room temperature) with α-CD: a) before and b) after addition of non-irradiated solution **B**. Switching *trans* to *cis* with λ = 365 nm (br.) (Supplementary Table **1**, entry **2**; ENB-280C/FE,). The spectra for the photostationary states are given.

Reversible Photoswitching of Bound *cyclized*-8

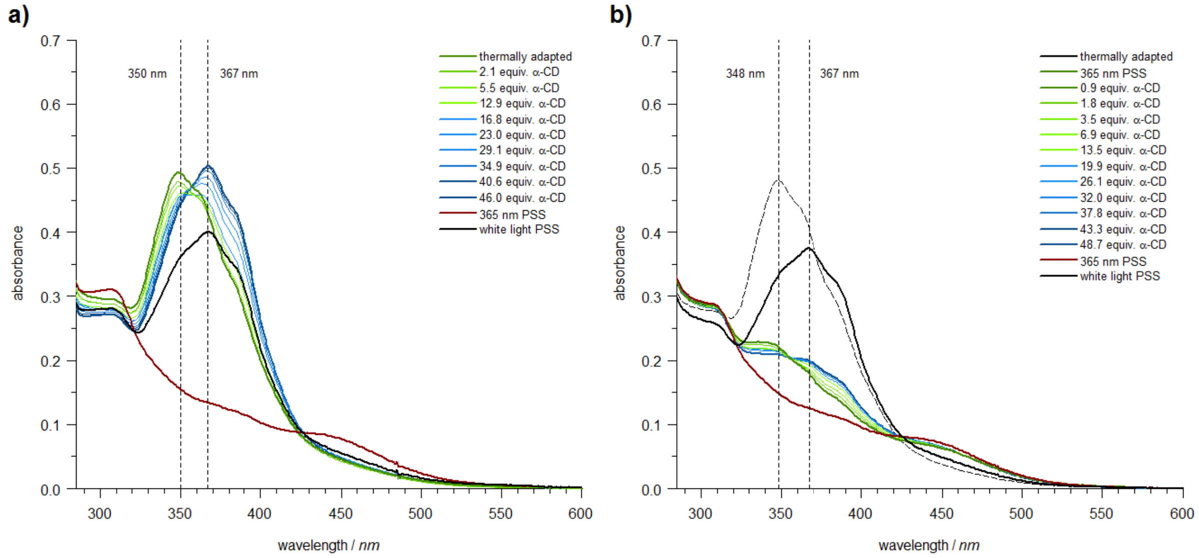

Supplementary Figure 68 | Absorption spectra for the photoisomerization and binding of compound ***cyclized*-8** (λ_{max} = 350 nm; 11 µM in water pH ≥ 9; room temperature) to α-CD: a) titration of the thermally adapted state and b) titration of the PSS reached for λ = 365 nm (br.) (Supplementary Table **1**, entry **2**; ENB-280C/FE,). At the end-point of the titration, the sample was irradiated with λ = 365 nm (br.) and subsequently with white light (Supplementary Table **1**, entry **12**; OSL1-EC,). The spectra for the photostationary states are given.

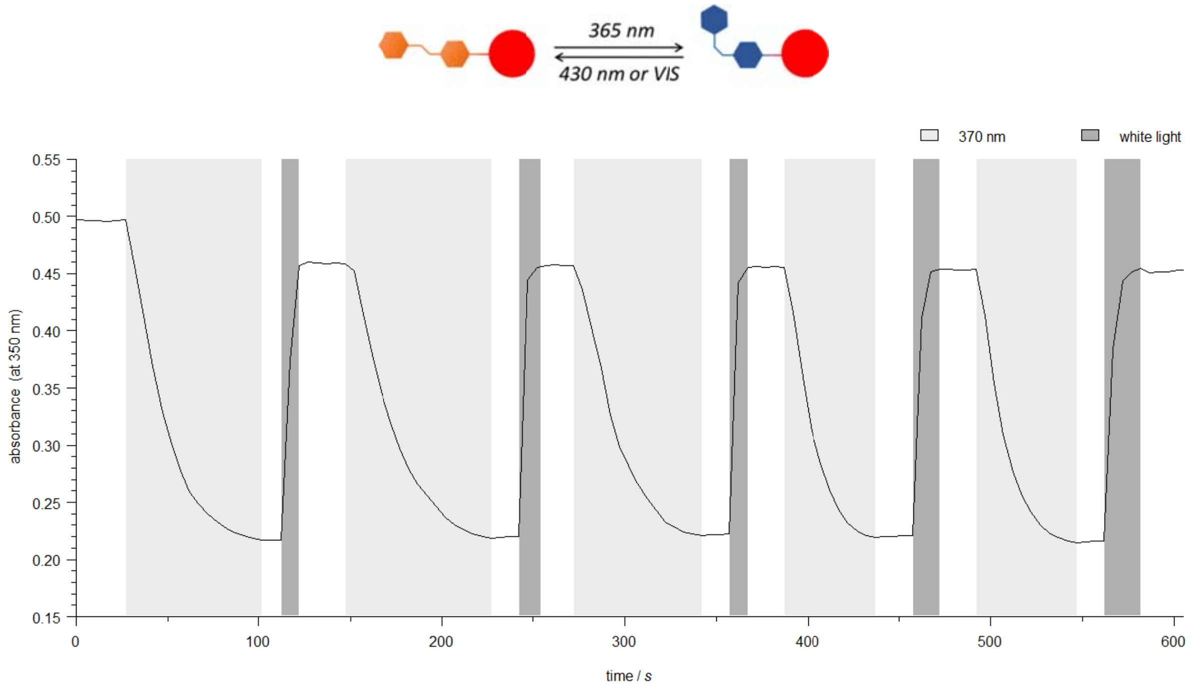

Supplementary Figure 69 | Reversible photochromism for repeated switching cycles of compound ***cyclized*-8** (11 µM in water pH ≥ 9; room temperature) observed at λ_{max} = 350 nm: Switching with λ = 370 nm (*trans-cis*; Supplementary Table **1**, entry **4**; MARL 260019 UV EMITTER, TO-46, 100DEG,) and switching back with white light (*cis-trans*; Supplementary Table **1**, entry **12**; OSL1-EC,).

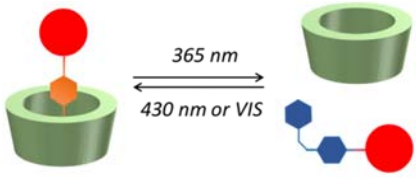

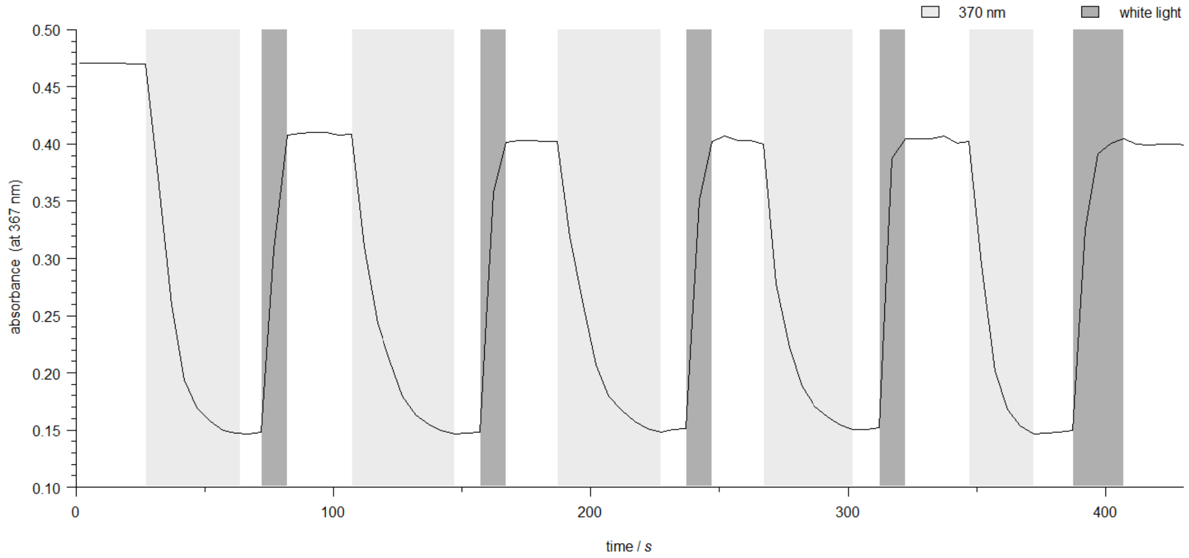

Supplementary Figure 70 | Reversible photochromism for repeated switching cycles of compound ***cyclized*-8** (11 µM in water pH ≥ 9; room temperature) and α-CD (140 µM, 12.7 equiv.) observed at λ_{max} = 367 nm: Switching with λ = 370 nm (*trans-cis*; Supplementary Table **1**, entry **4**; MARL 260019 UV EMITTER, TO-46, 100DEG,) and switching back with white light (*cis-trans*; Supplementary Table **1**, entry **12**; OSL1-EC,).

¹H- and ¹³C-NMR Spectra

Compound 1

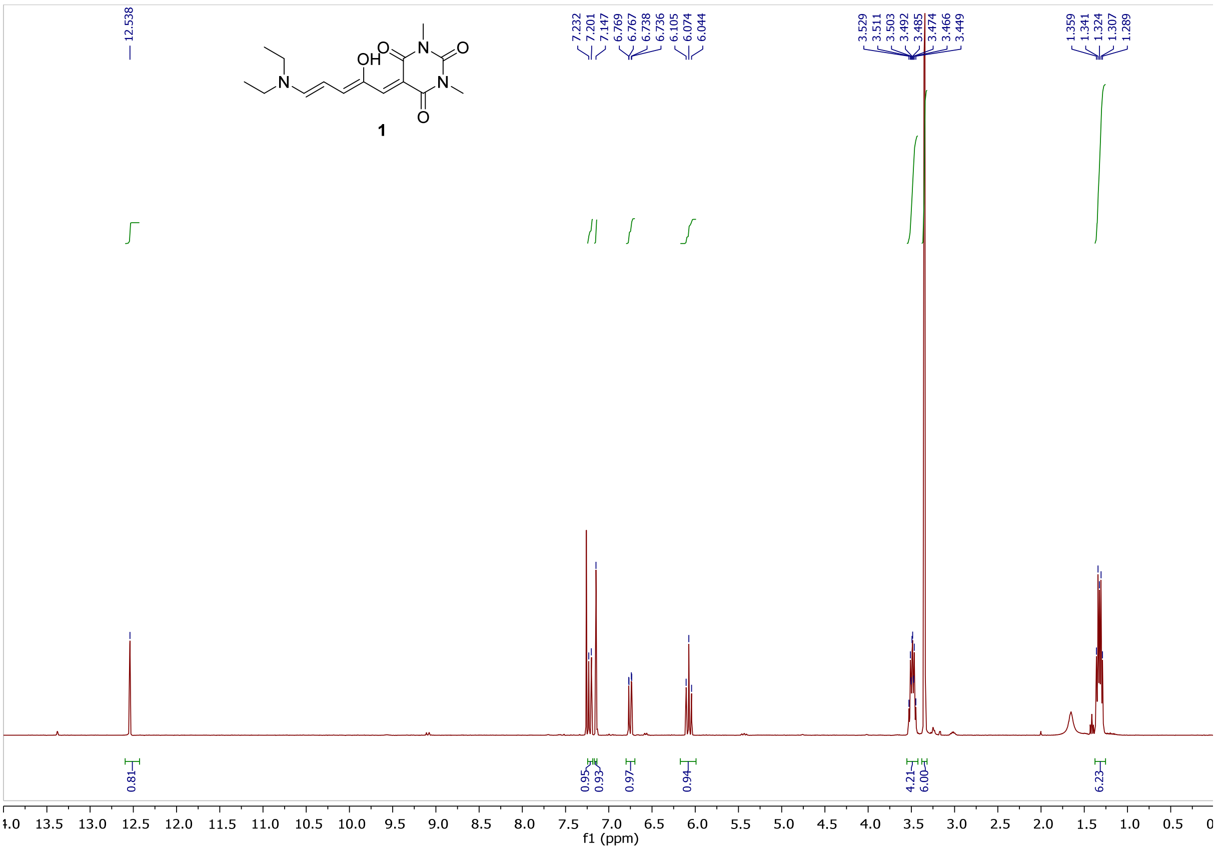

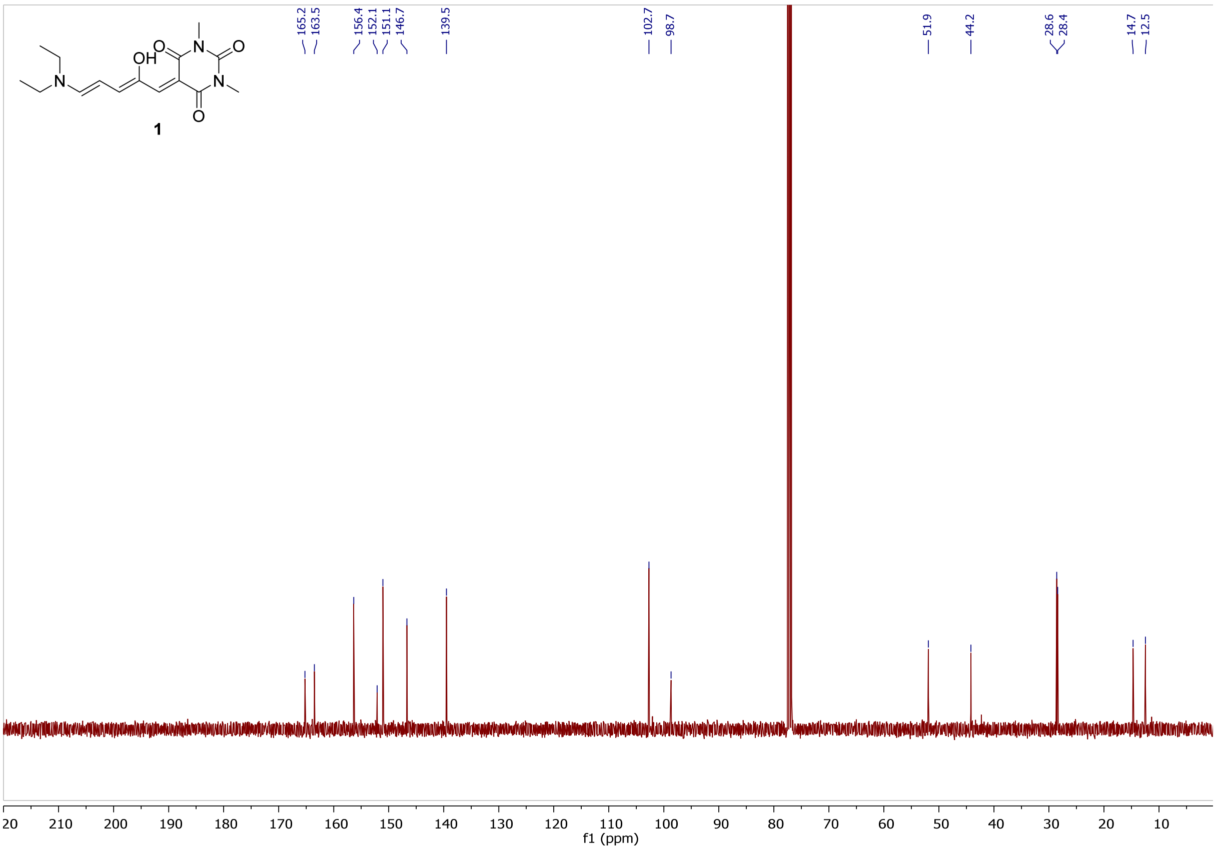

Supplementary Figure 71 | ¹H- and ¹³C{¹H}-NMR spectra of compound **1**.

Compound 2

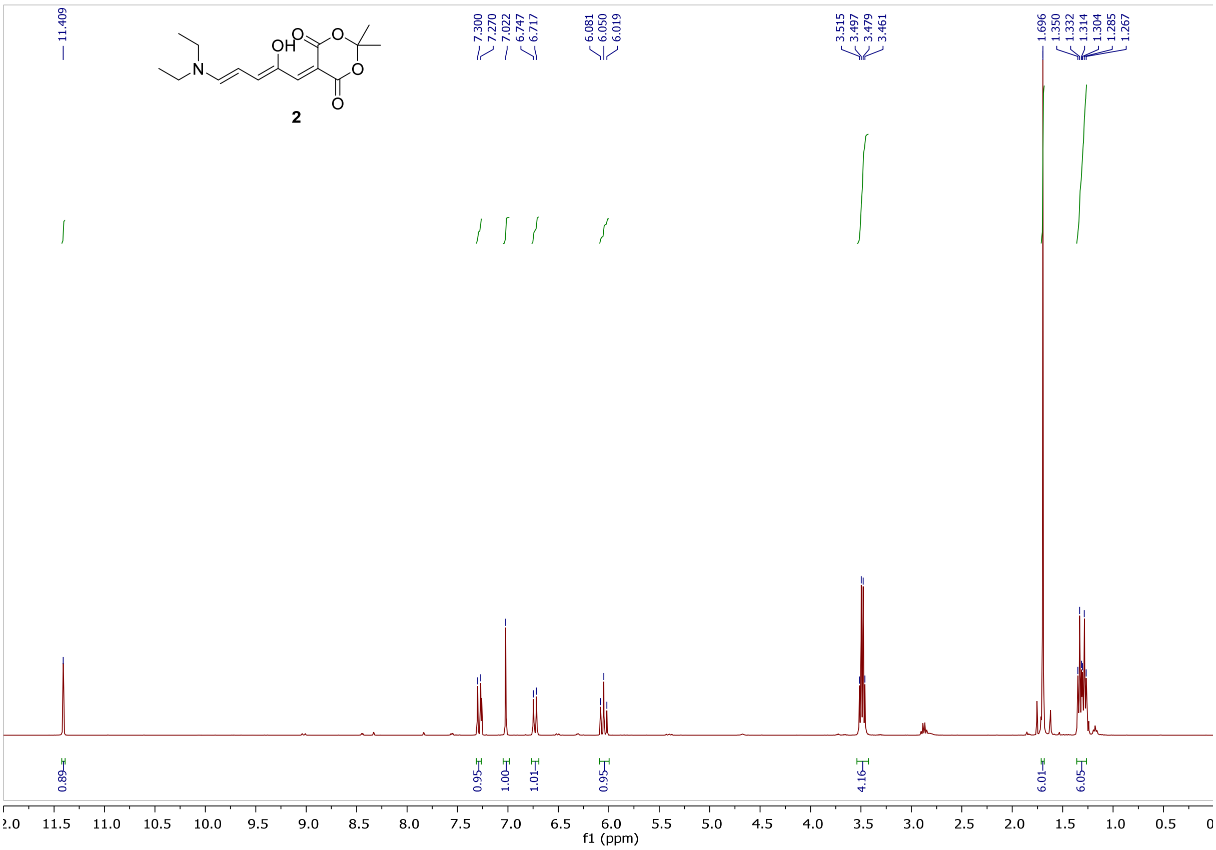

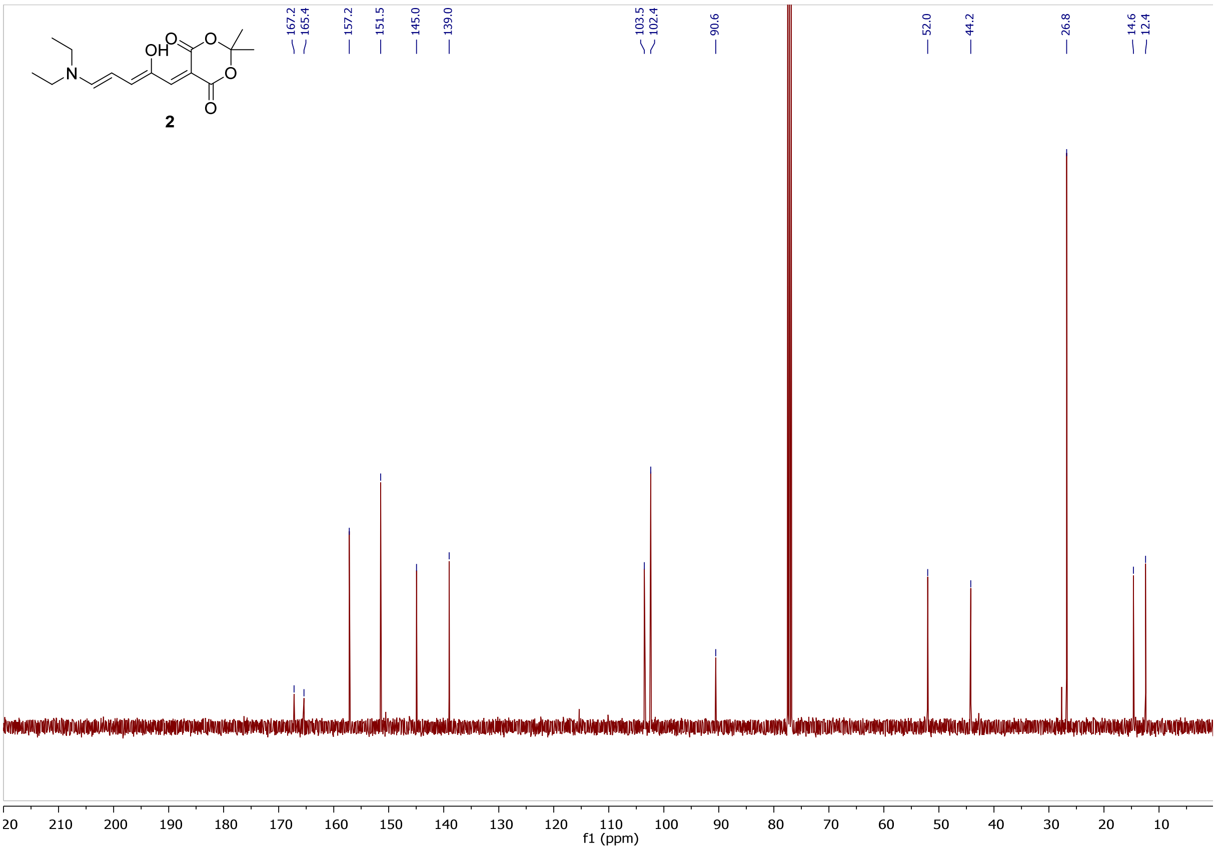

Supplementary Figure 72 | ^{1}H- and ^{13}C{^{1}H}-NMR spectra of compound **2**.

Compound 3

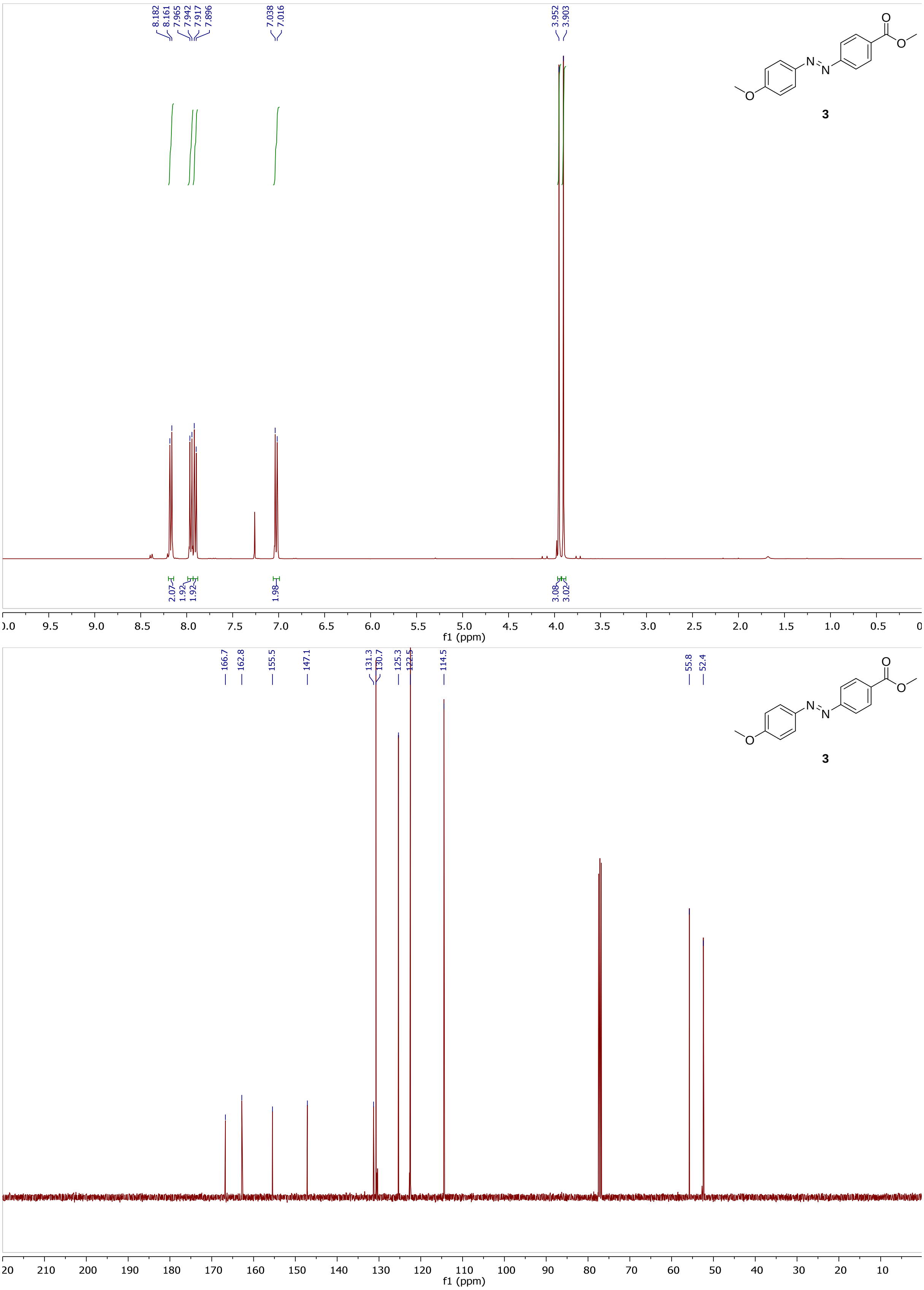

Supplementary Figure 73 | ^{1}H- and ^{13}C{^{1}H}-NMR spectra of compound **3.**

Compound 4

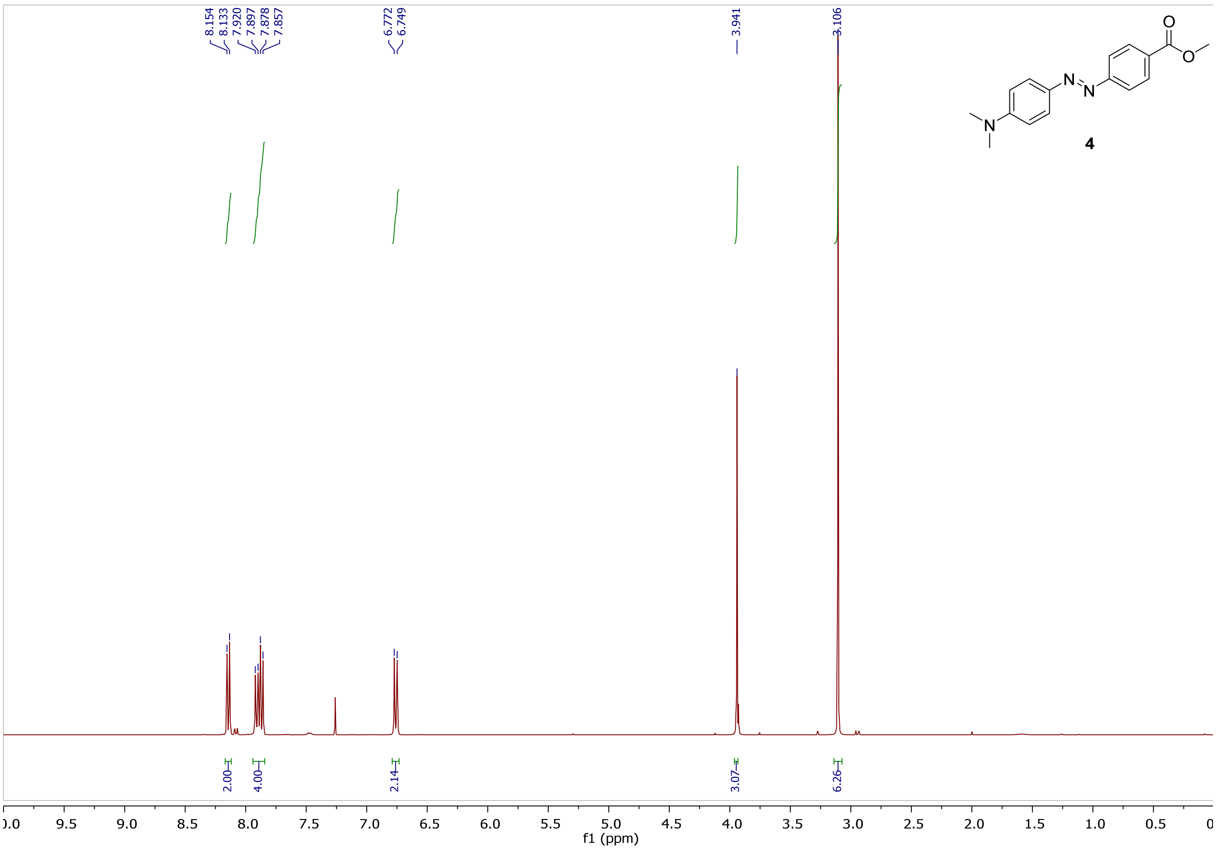

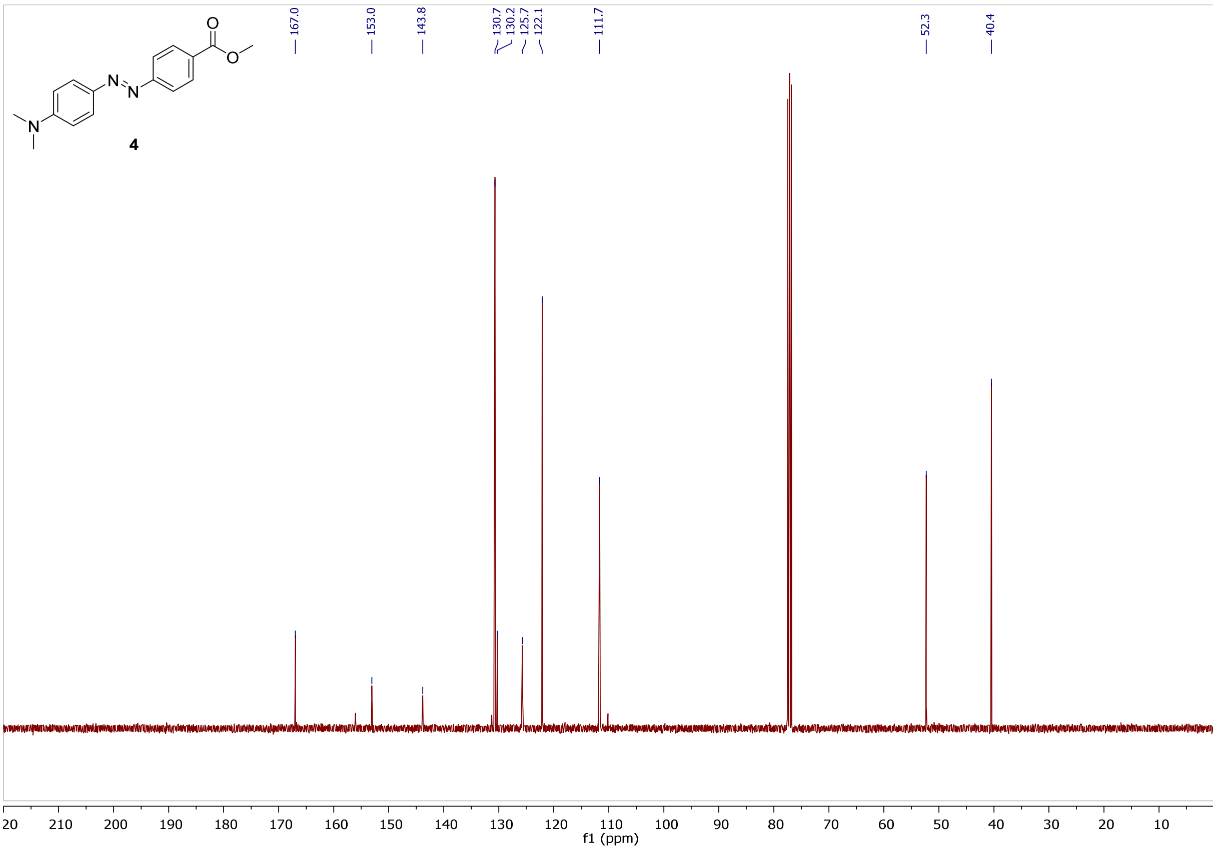

Supplementary Figure 74 | ^{1}H- and $^{13}C\{^{1}H\}$-NMR spectra of compound **4.**

Compound S5

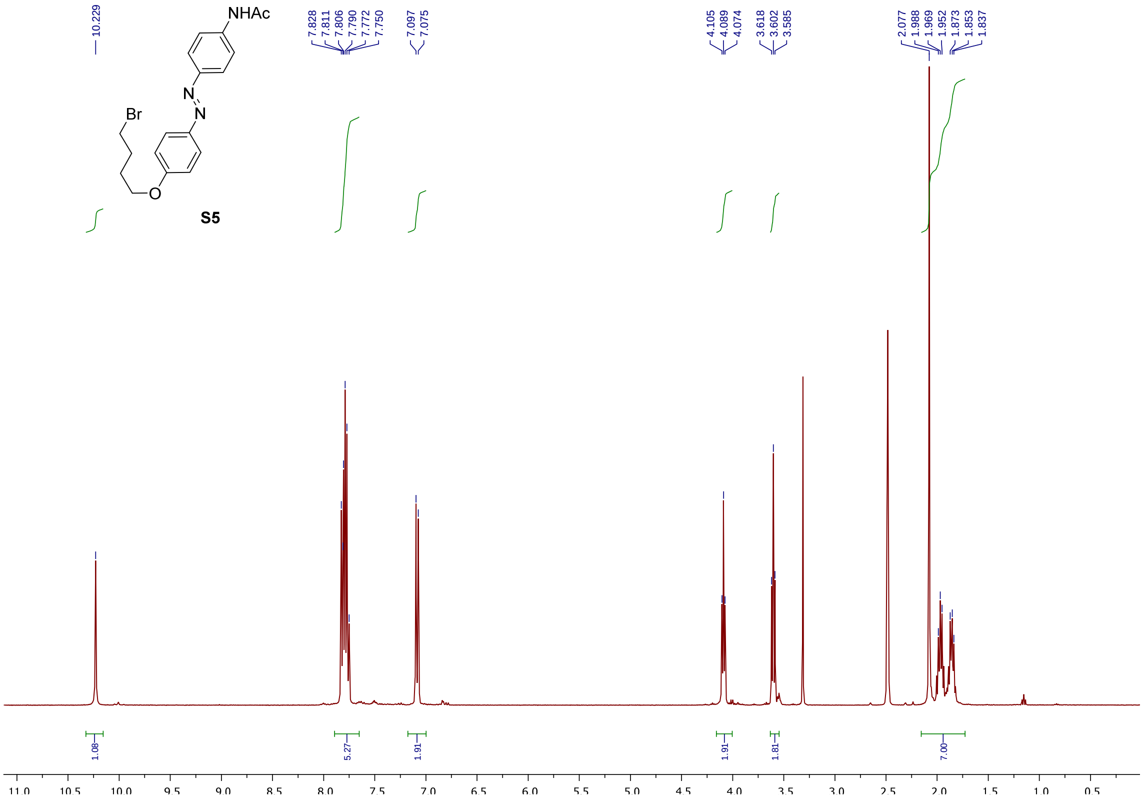

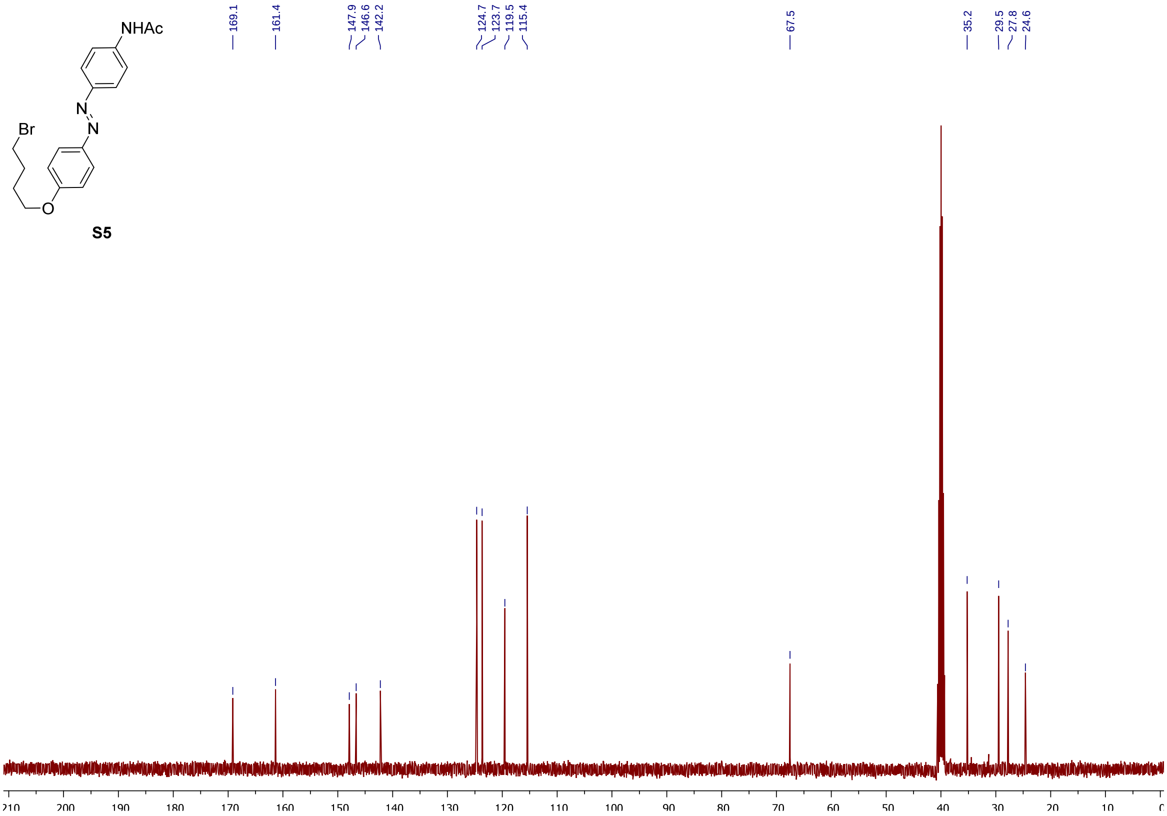

Supplementary Figure 75 | ^{1}H- and ^{13}C{^{1}H}-NMR spectra of compound **5.**

Compound S6

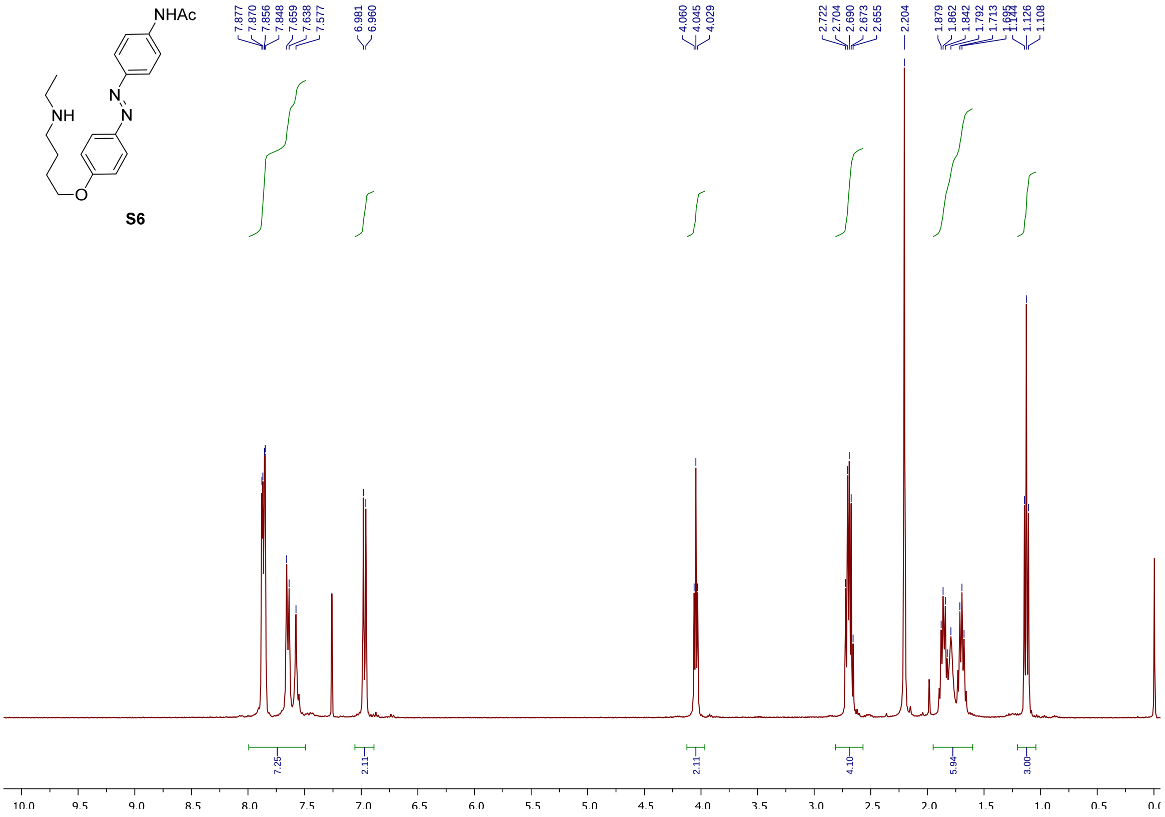

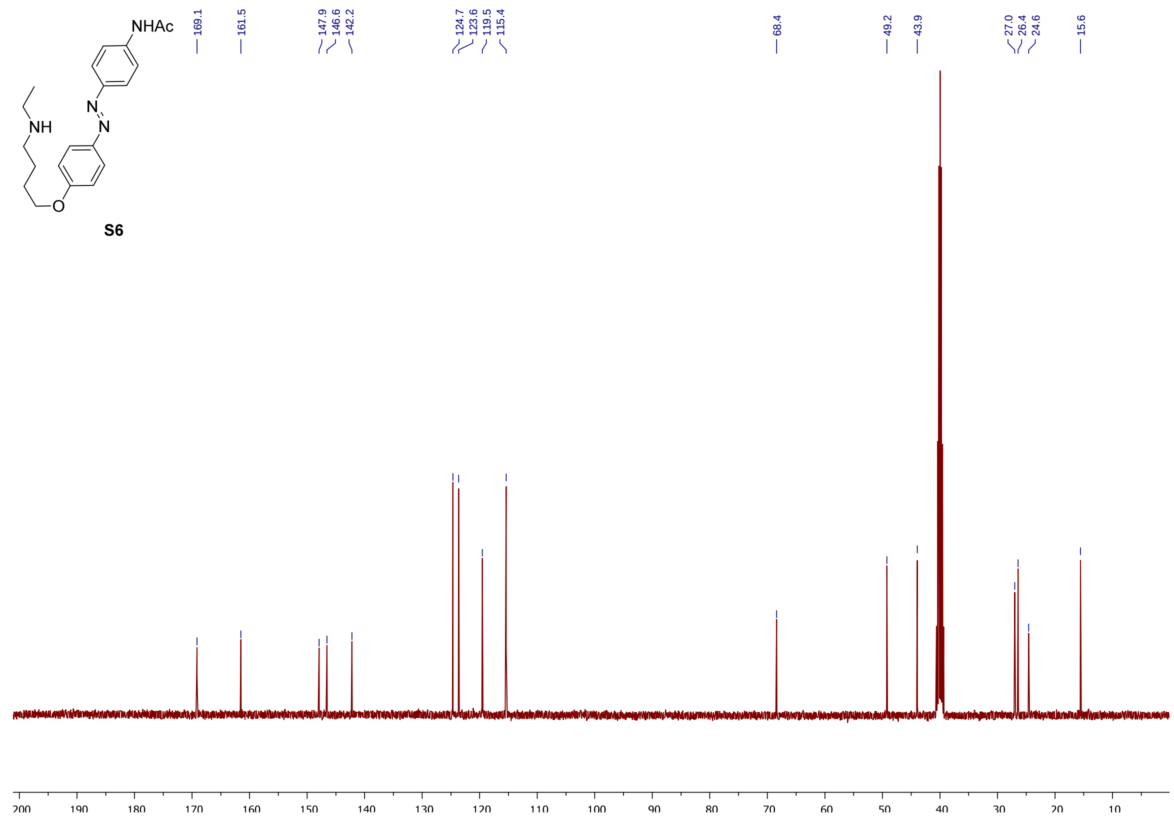

Supplementary Figure 76 | ^{1}H- and ^{13}C{^{1}H}-NMR spectra of compound **6.**

Compound 7

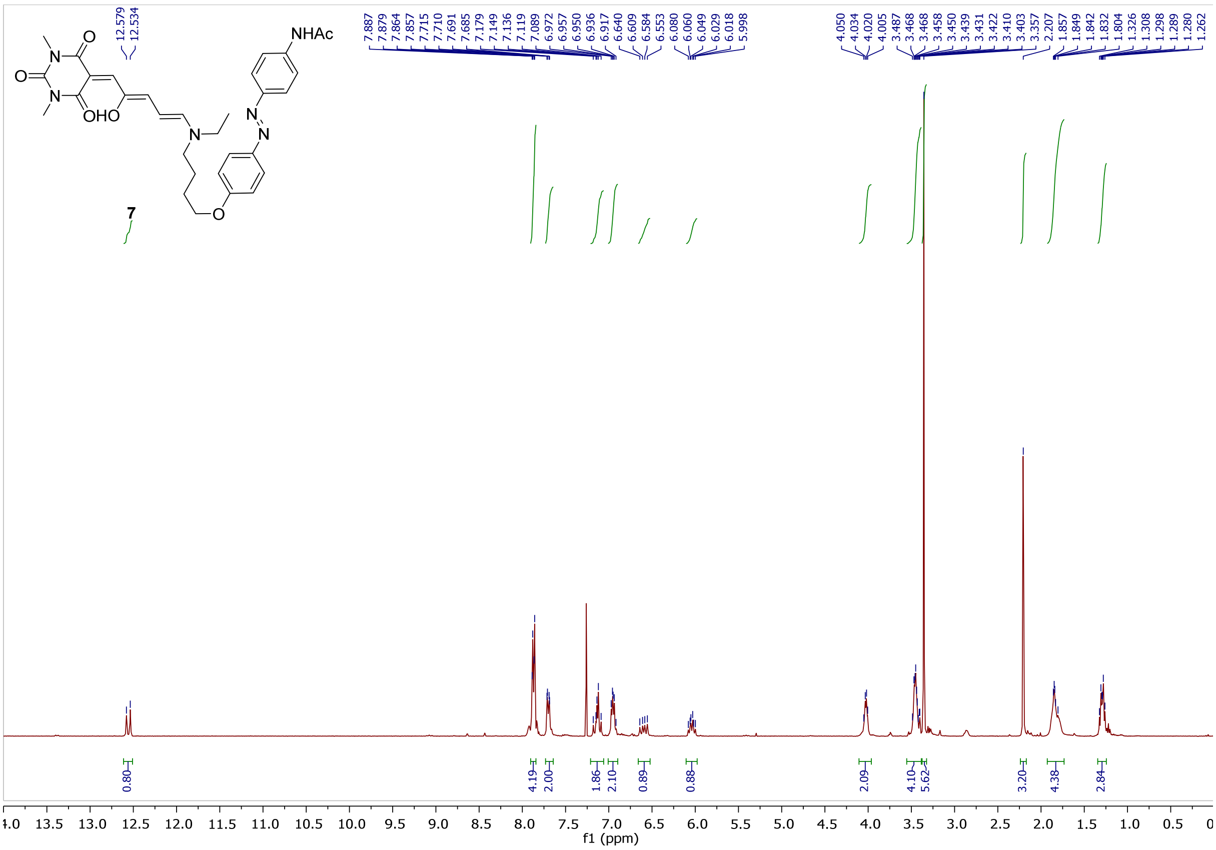

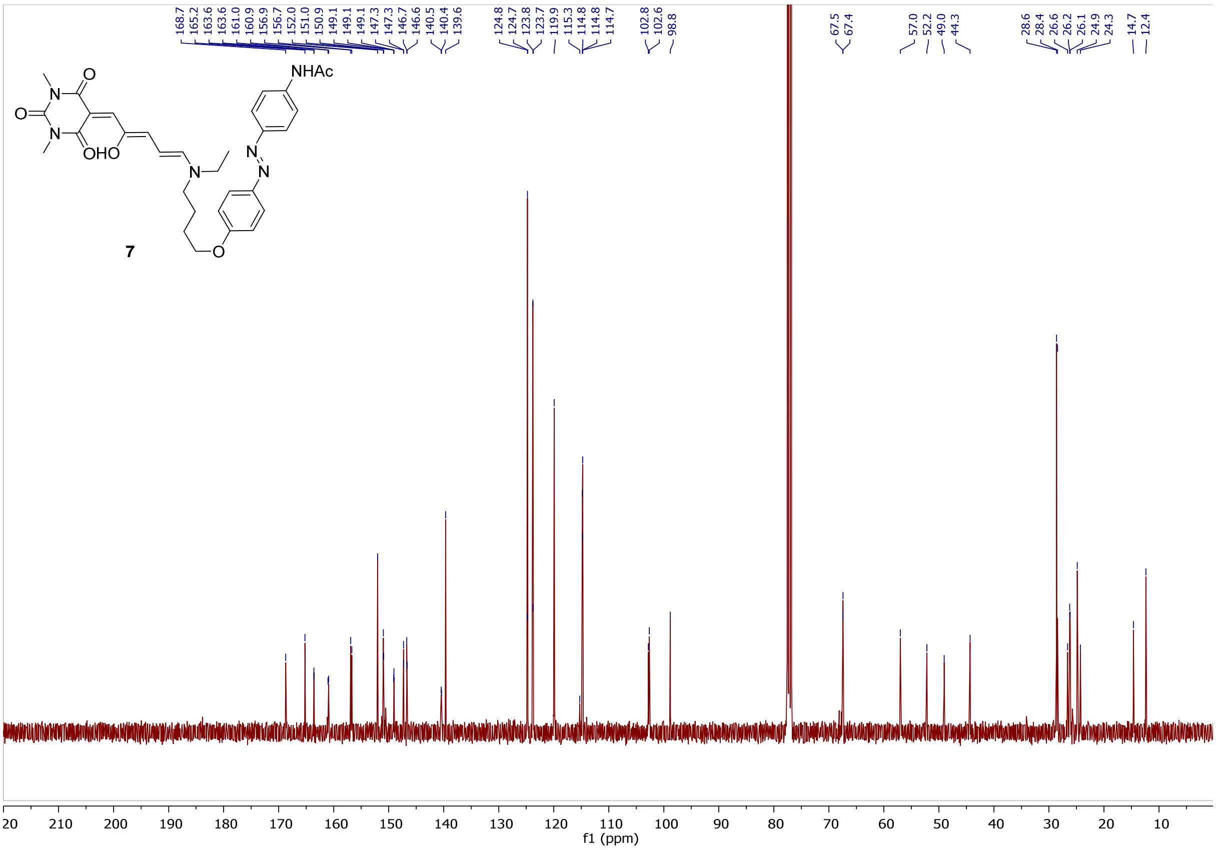

Supplementary Figure 77 | ^{1}H- and $^{13}C\{^{1}H\}$-NMR spectra of compound **7.**

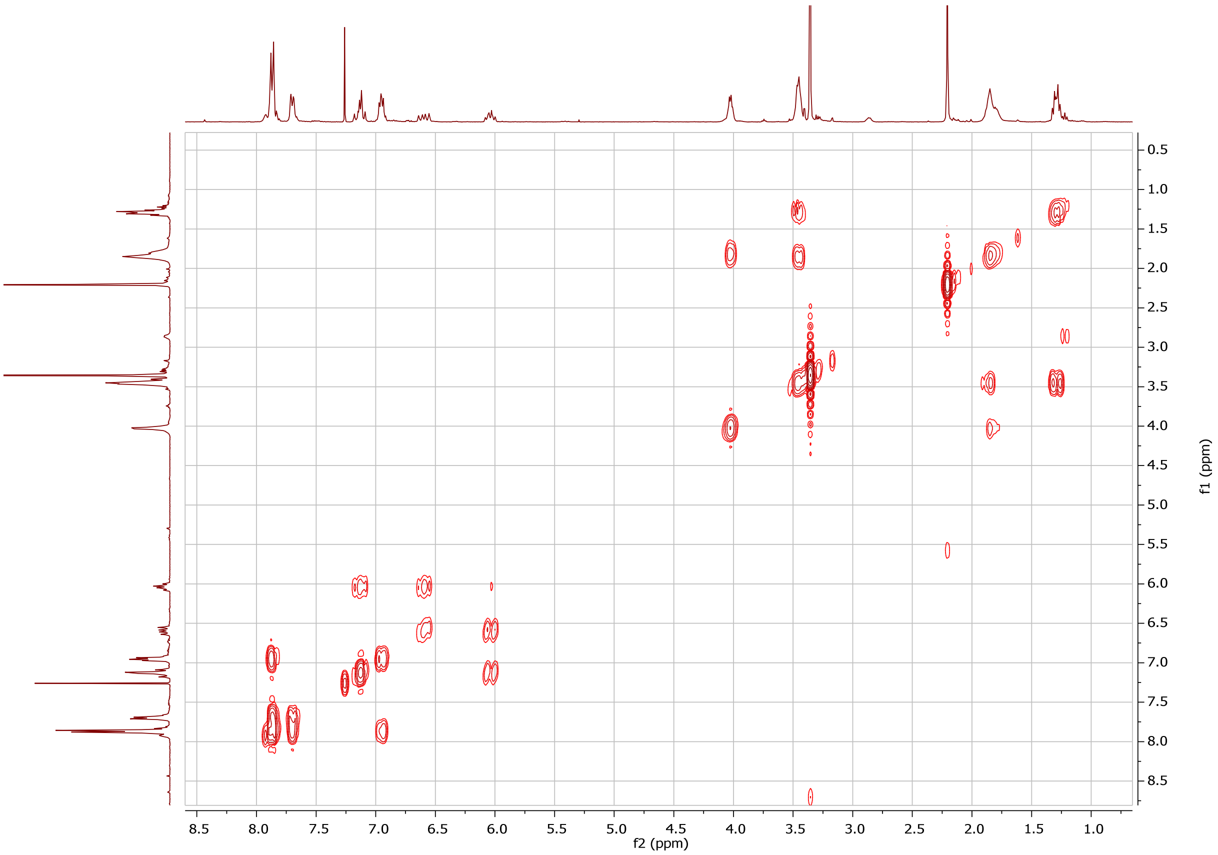

Supplementary Figure 78 | ^{1}H- COSY-NMR spectrum of compound **7.**

Compound S7

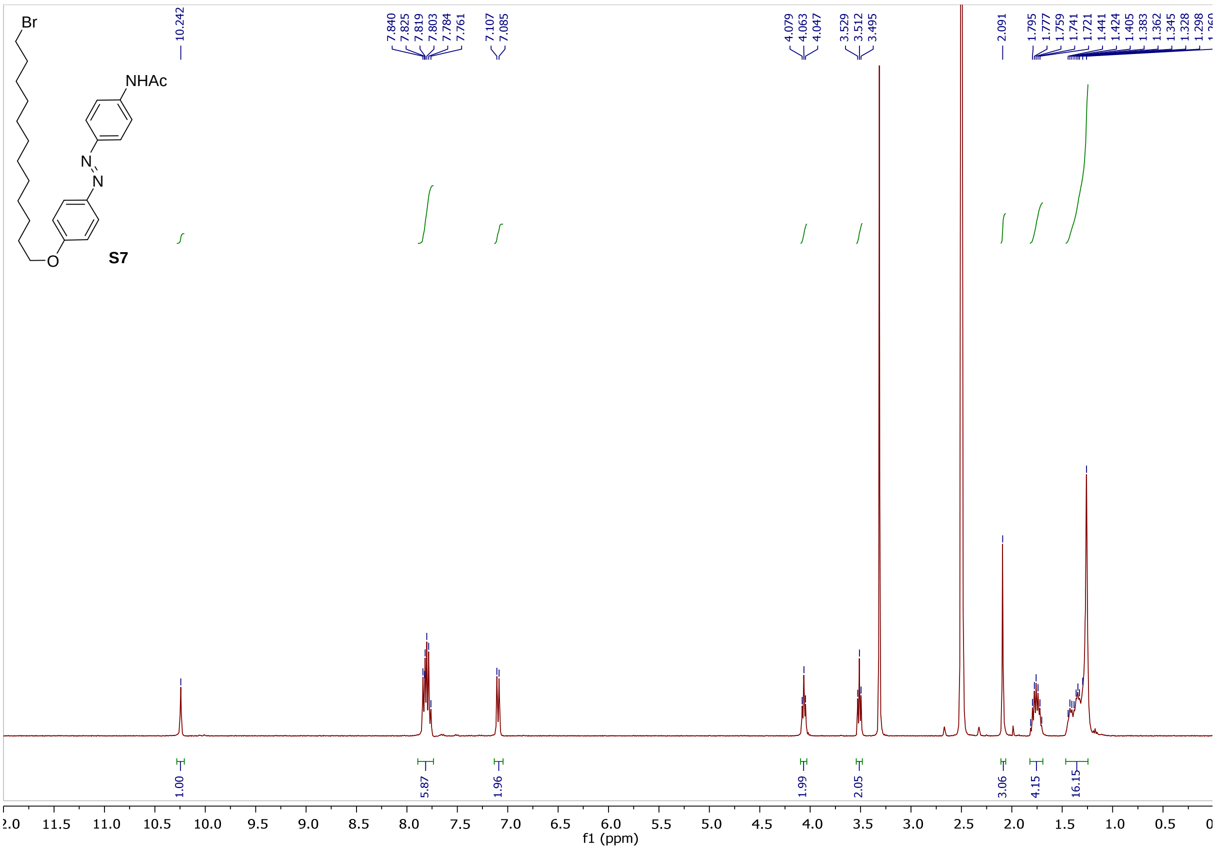

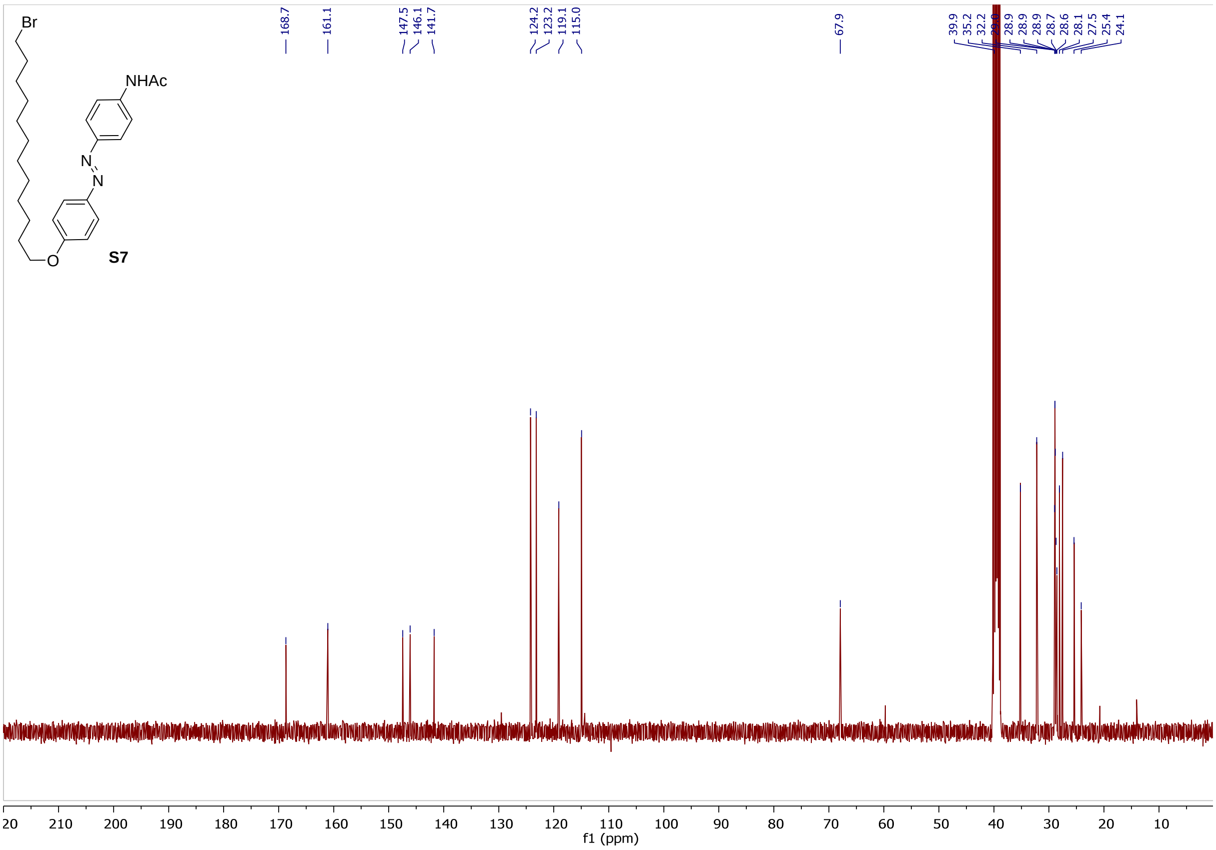

Supplementary Figure 79 | ^{1}H- and ^{13}C{^{1}H}-NMR spectra of compound **S7.**

Compound S8

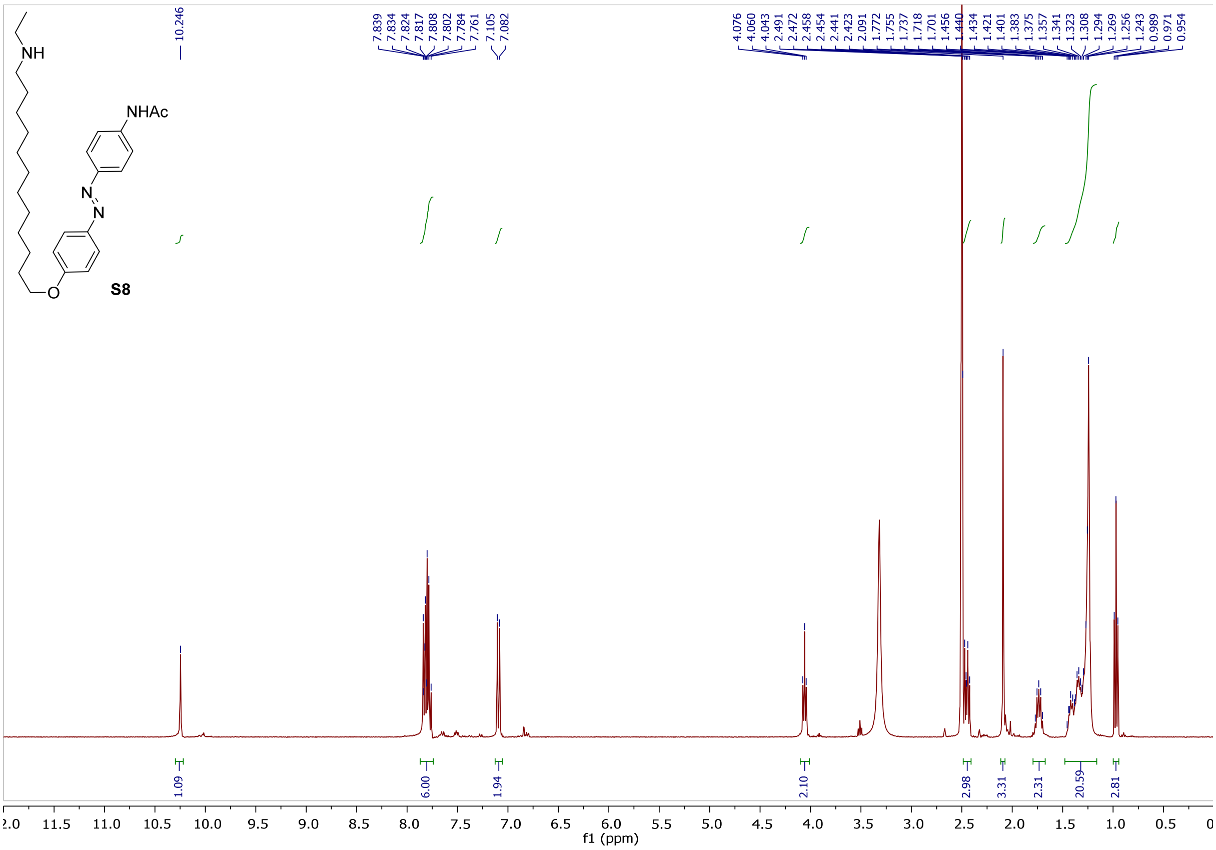

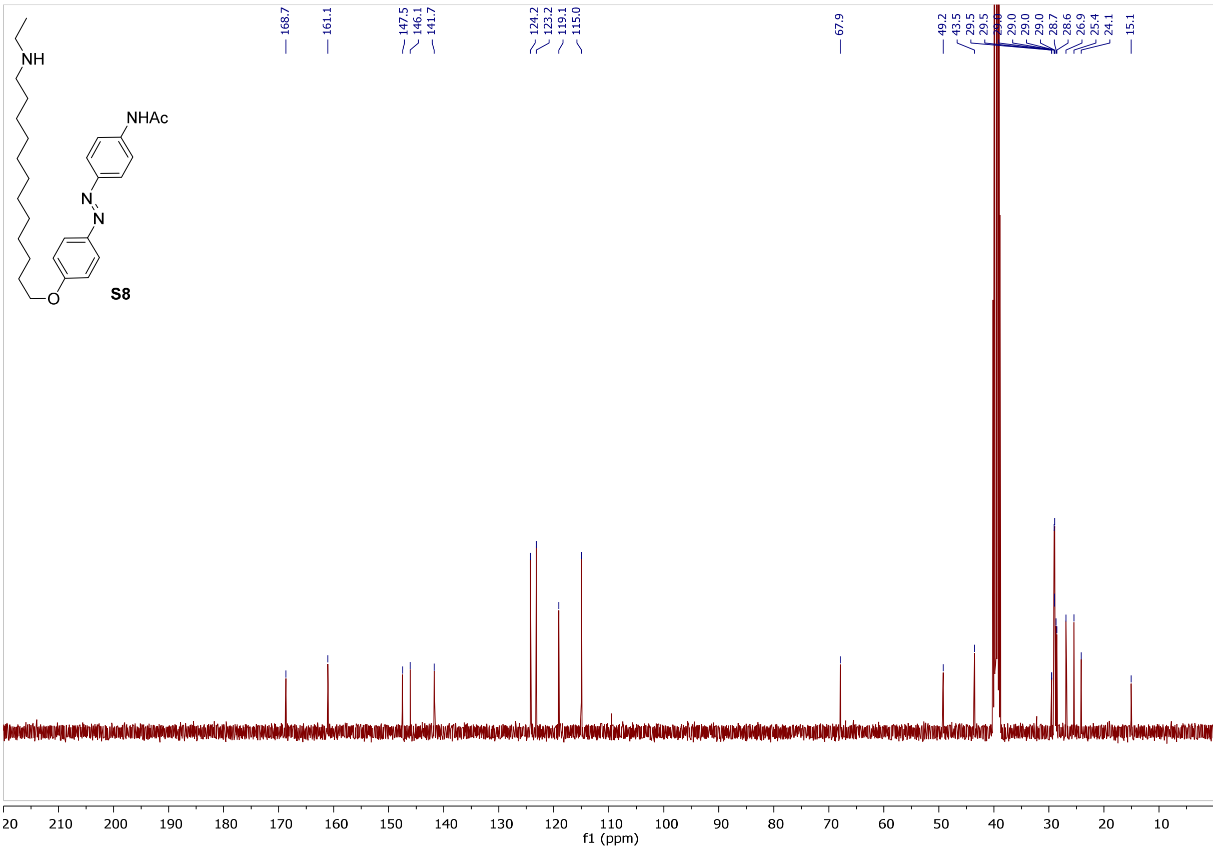

Supplementary Figure 80 | ^{1}H- and $^{13}C\{^{1}H\}$-NMR spectra of compound **S8.**

Compound 8

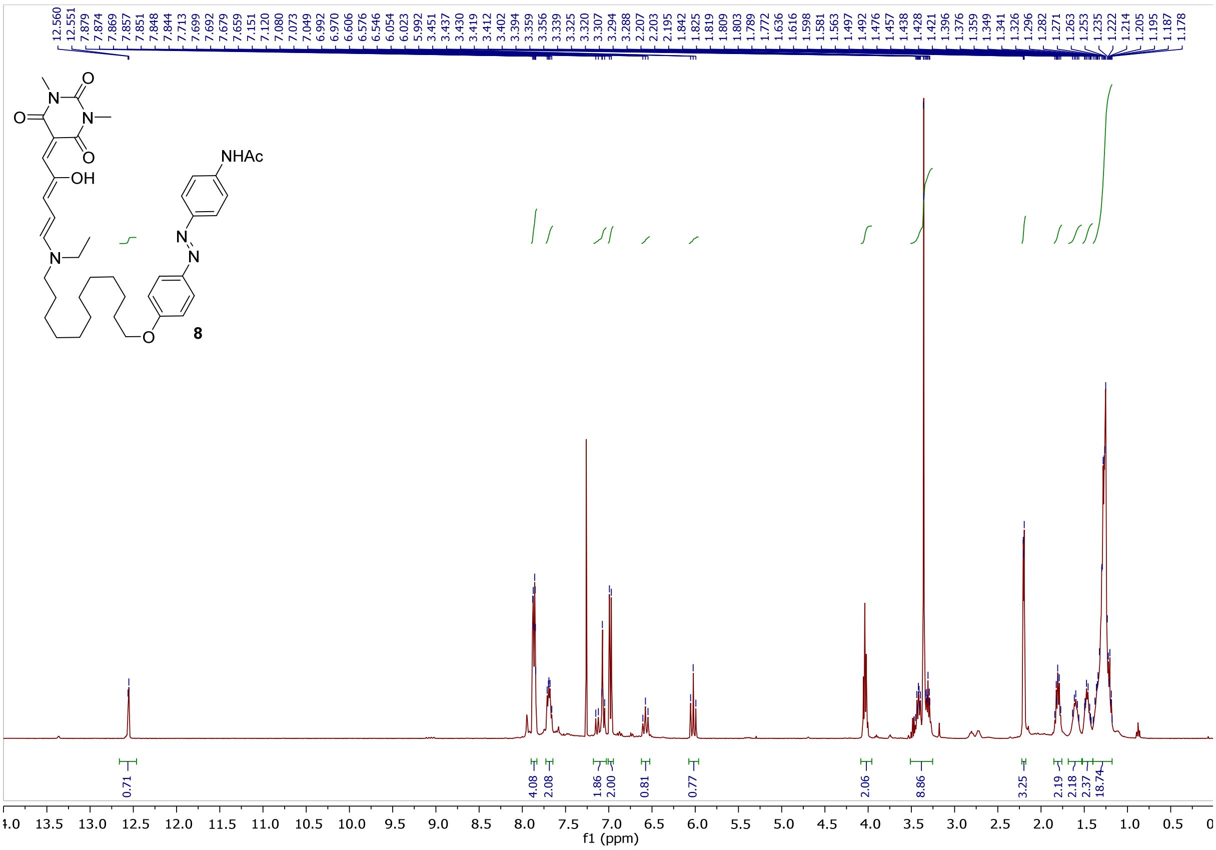

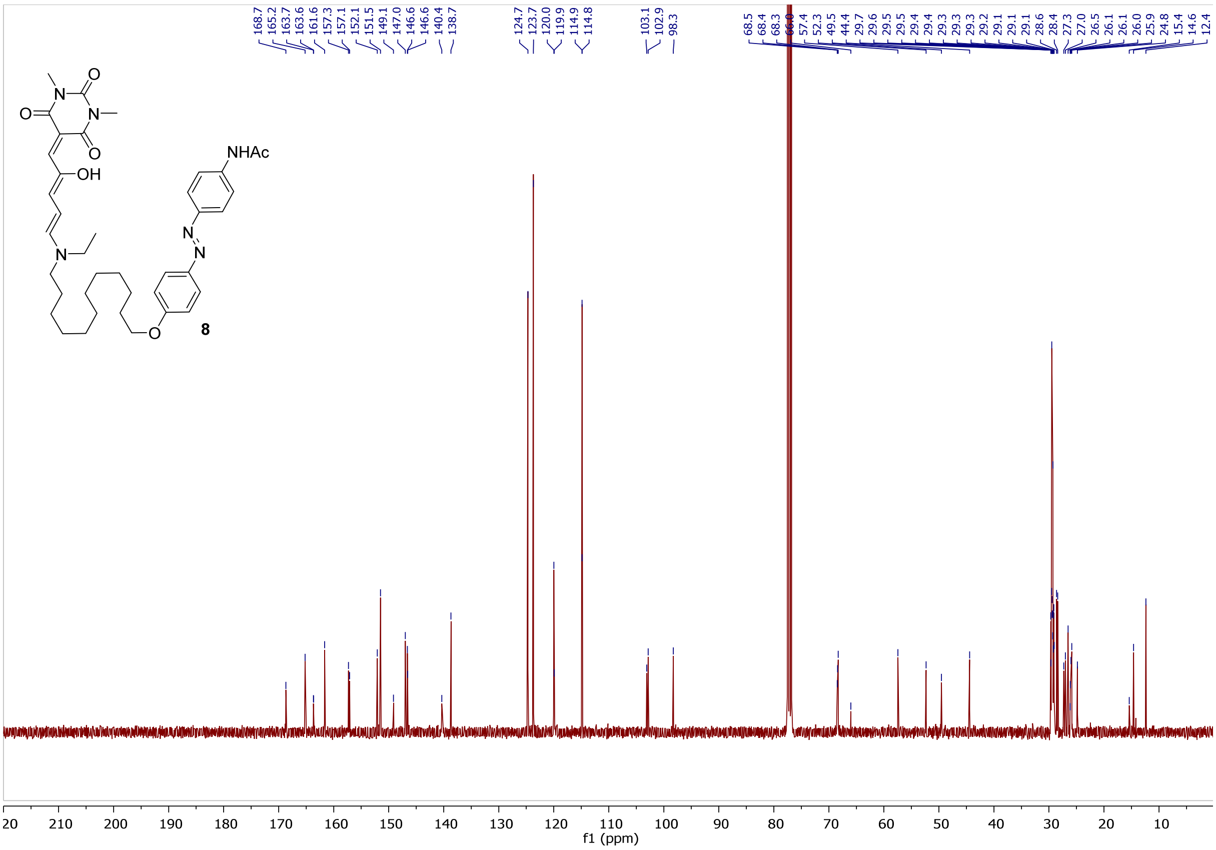

Supplementary Figure 81 | ^{1}H- and ^{13}C{^{1}H}-NMR spectra of compound **8.**

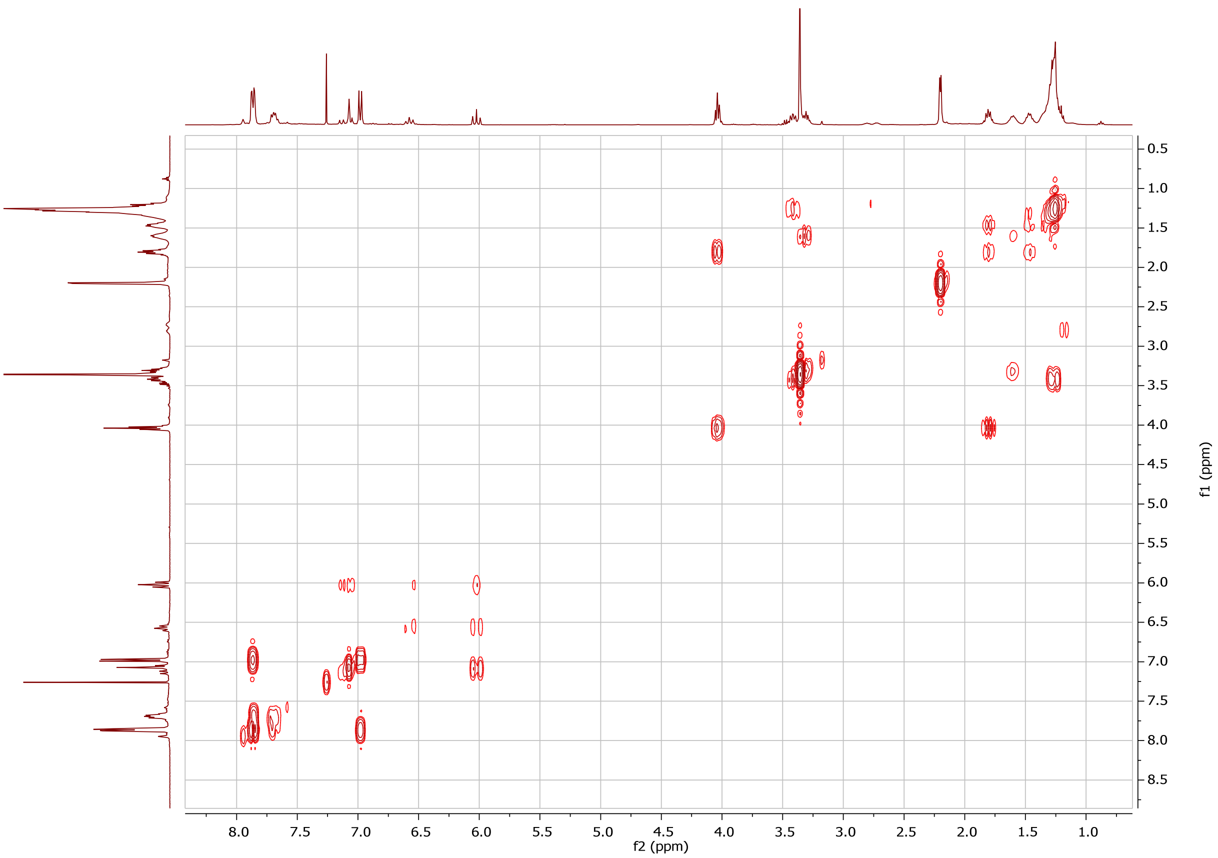

Supplementary Figure 82 | ^{1}H-COSY-NMR spectrum of compound **8.**

Compound 9

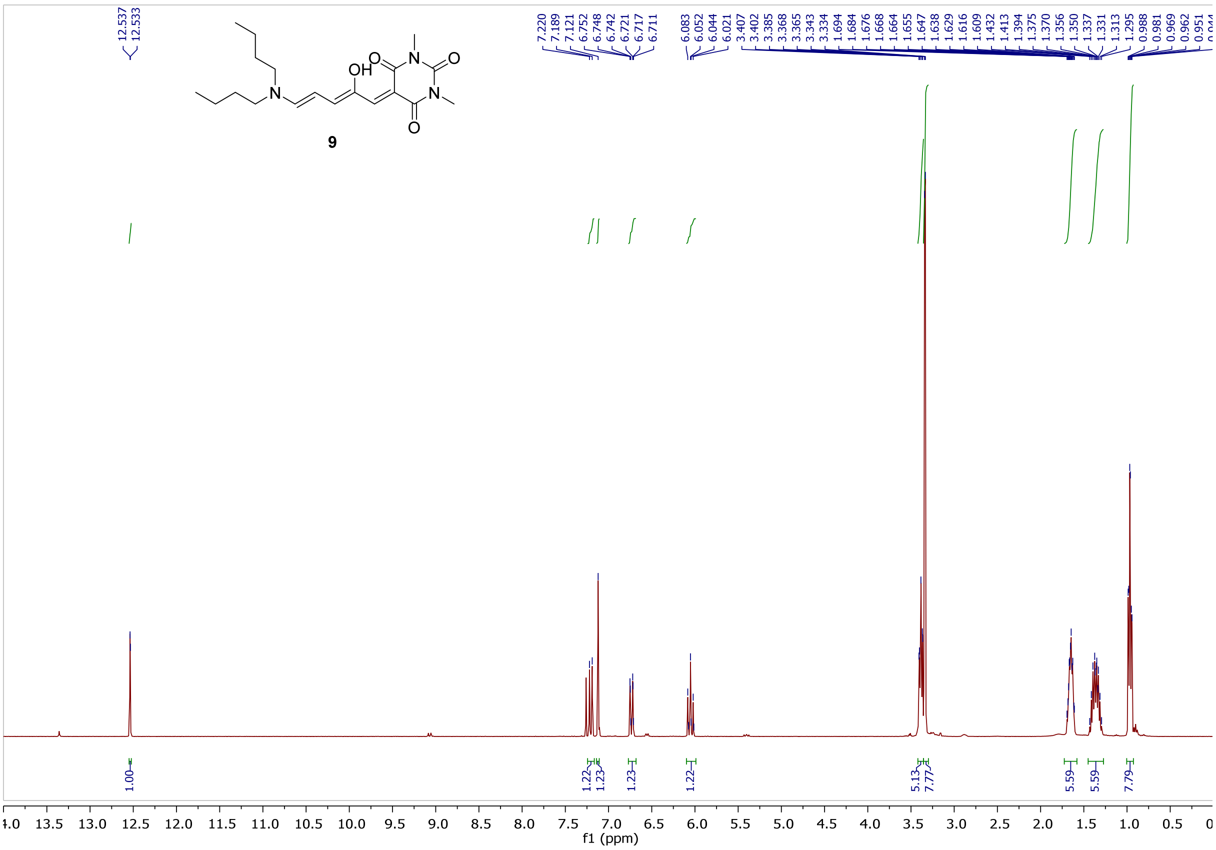

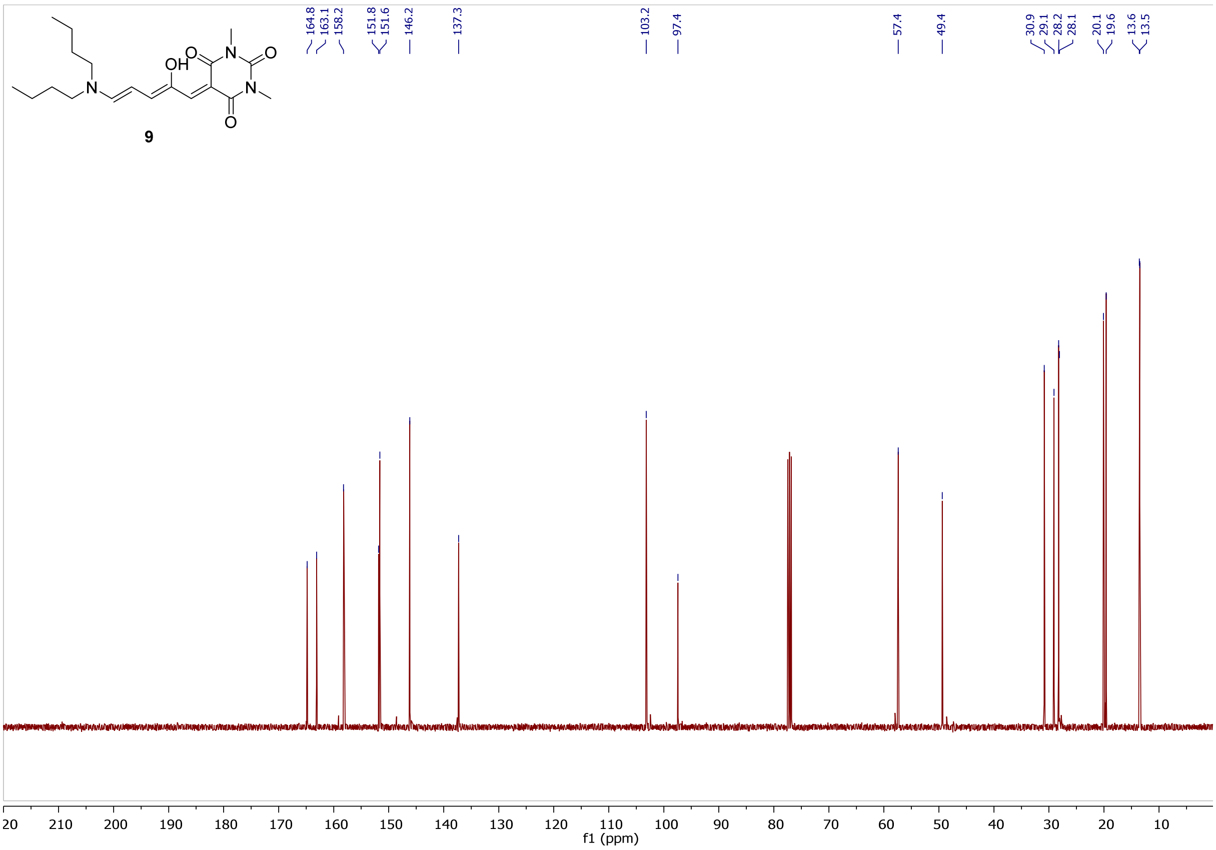

Supplementary Figure 83 | ^{1}H- and $^{13}C\{^{1}H\}$-NMR spectra of compound **9.**

Light Sources

Supplementary Table 1 | Comparison of used commercial light sources.

entry	color[a]	λ_{max} (nm)[b]	intensity (mW)[c]	type	supplier
**1**		312	*ND*	ENB-280C/FE	Spectroline
**2**		365 (br.)	*ND*	ENB-280C/FE	Spectroline
**3**		365	1 x [4.1]	M365F1	ThorLabs
**4**		370	5 x [1.0]	MARL 260019 UV EMITTER, TO-46, 100DEG	Farnell
**5**		400	9.1[d]	LED light source 3x 400 nm	Sahlmann Photochemical Solutions
**6**		430	0.4[e]	607-4304H6	Mouser
**7**		460	0.7[d]	604-APTD1608QBC/D	Mouser
**8**		471	12[e]	859-LTL2R3TBV3KS	Mouser
**9**		515	1.1[d]	604-APTD1608ZGC	Mouser
**10**		590	1.0[d]	604-APTD1608SYC/J3	Mouser
**11**		640	2.5[d]	604-APTD1608SEC/J3	Mouser
**12**		white light (wl)	*ND*	OSL1-EC	ThorLabs
**13**		white light (halogen)	*ND*	Plusline ES Small 160W 3100 lm	Philips

[a] Color code used throughout the manuscript and supporting information; [b] Absorption maximum (λ_{max}) of the light source as indicated by the supplier. [c] Values in [] denote intensities reported by the supplier. *ND* = not determined [d] Distance from light source: 5 cm. [e] Distance from light source: 1 cm.

Supplementary Table 2 | Used optical band-pass filters were purchased from Andover Corporation and used in combination with the high-intensity white light source (Supplementary Table 1, entry 12; OSL1-EC,).

entry	color[a]	λ_{center} (nm)[b]	Bandwidth (nm)[b]	Transmission (%)[b]	type
**1**		430.0 +3/-0	10.0 +2/-2	45	430FS10-50
**2**		546.1 +2/-0	10.0 +2/-2	55	546FS10-50
**3**		577.0 +2/-0	10.0 +2/-2	55	577FS10-50

[a] Color code used throughout the manuscript and supporting information; [b] As indicated by the supplier.

Titrations

Thermally adapted ***cyclized*-8**

Supplementary Table 3 | Amounts of solutions **A** and **B** and concentrations obtained for the titration of thermally adapted ***cyclized*-8.**[a,b] All solution were prepared in water (pH ≥ 9; room temperature). The concentration of ***cyclized*-8** was hold constant during the titration.

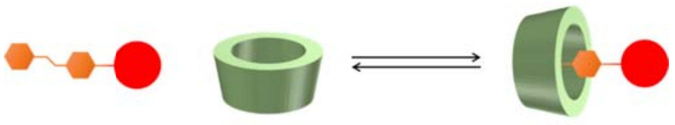

entry	**B** added (µL)	total amount (µL)	[***cyclized*-8**] conc. (µM)	**α-CD** conc. (µM)	equiv. α-CD
1		2070	11	0	0
2	1	2071	11	1.9	0.18
3	2	2072	11	3.9	0.35
4	4	2074	11	7.7	0.70
5	8	2078	11	15.4	1.40
6	12	2082	11	23.1	2.10
7	16	2086	11	30.7	2.79
8	20	2090	11	38.3	3.48
9	24	2094	11	45.9	4.17
10	28	2098	11	53.4	4.85
11	32	2102	11	60.9	5.54
12	36	2106	11	68.4	6.22
13	40	2110	11	75.8	6.89
14	52	2122	11	98.0	8.91
15	64	2134	11	112.0	10.91
16	76	2146	11	141.7	12.88
17	88	2158	11	163.1	14.83
18	100	2170	11	184.3	16.76

19	140	2210	11	253.4	23.04
20	180	2250	11	320.0	29.09
21	220	2290	11	384.3	34.93
22	260	2330	11	446.4	40.58
23	300	2370	11	506.3	46.03

[a] **Solution A:** 11 µM *cyclized*-8; **Solution B:** 11 µM *cyclized*-8; 4 mM α–CD; [b] *non-irradiated/thermally adapted.*

PSS 365 nm ***cyclized*-8**

Supplementary Table 4 | Amounts of solutions **A** and **B** and concentrations obtained for the titration of irradiated ***cyclized*-8** for 180s λ = 365 nm (br.) (Supplementary Table **1**, entry **2**; ENB-280C/FE,).[a,b] All solution were prepared in water (pH ≥ 9; room temperature). The concentration of ***cyclized*-8** was hold constant during the titration. Both solutions were irradiated concomitantly.

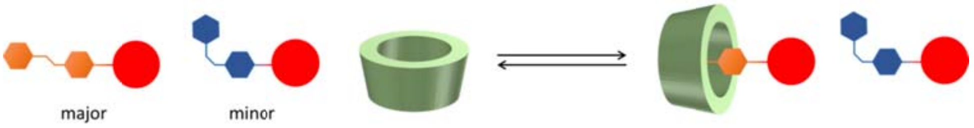

entry	**B** added (µL)	total amount (µL)	[*cyclized*-**8**] conc. (µM)	**α-CD** conc. (µM)	equiv. α-CD
1		2070	11	0	0
2	40	2110	11	75.8	6.89
3	80	2150	11	148.8	13.53
4	120	2190	11	219.2	19.93
5	160	2230	11	287.0	26.09
6	200	2270	11	352.4	32.04
7	240	2310	11	415.6	37.78
8	280	2350	11	476.6	43.33
9	320	2390	11	535.6	48.69

[a] **Solution A:** 11 µM *cyclized*-8; **Solution B:** 11 µM *cyclized*-8; 4 mM α–CD; [b] *Irradiation:* 180s λ = 365 nm (br.) (Supplementary Table **1**, entry **2**; ENB-280C/FE,).

Control experiment 1

Supplementary Table 5 | Amounts of solutions **A** and **B** and concentrations obtained for the titration of irradiated ***cyclized*-8** for 30s λ = 365 nm (br.) (Supplementary Table **1**, entry **2**; ENB-280C/FE,).[a,b] All solution were prepared in water (pH ≥ 9; room temperature). The concentration of ***cyclized*-8** was hold constant during the titration. Both solutions were irradiated concomitantly.

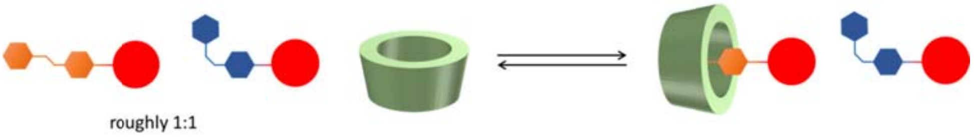

entry	**B** added (µL)	total amount (µL)	[*cyclized*-**8**] conc. (µM)	**α-CD** conc. (µM)	equiv. α-CD
1		2070	11	0	0

2	40	2110	11	75.8	6.89
3	80	2150	11	148.8	13.53
4	120	2190	11	219.2	19.93
5	160	2230	11	287.0	26.09
6	200	2270	11	352.4	32.04
7	240	2310	11	415.6	37.78
8	280	2350	11	476.6	43.33
9	320	2390	11	535.6	48.69

[a] **Solution A:** 11 µM *cyclized*-8; **Solution B:** 11 µM *cyclized*-8; 4 mM α–CD; [b] *Irradiation: 30s* λ = 365 nm (br.) (Supplementary Supplementary Table **1**, entry **2**; ENB-280C/FE,).

Control experiment 2

20s 365 nm ***cyclized*-8**

Supplementary Table 6 | Amounts of solutions **A** and **B** and concentrations obtained for the titration of irradiated ***cyclized*-8** for 20s λ = 365 nm (br.) (Supplementary Table **1**, entry **2**; ENB-280C/FE,).[a,b] All solution were prepared in water (pH ≥ 9; room temperature). The concentration of ***cyclized*-8** was hold constant during the titration. Only solution A was irradiated.

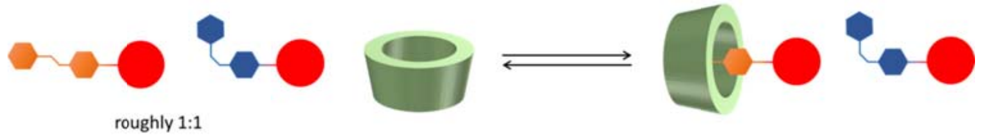

			[***cyclized*-8**]	**α-CD**	
entry	**B** added (µL)	total amount (µL)	conc. (µM)	conc. (µM)	equiv. α-CD
1		2070	11	0	0
2	40	2110	11	75.8	6.89
3	80	2150	11	148.8	13.53
4	120	2190	11	219.2	19.93
5	160	2230	11	287.0	26.09
6	200	2270	11	352.4	32.04
7	240	2310	11	415.6	37.78
8	280	2350	11	476.6	43.33
9	320	2390	11	535.6	48.69

[a] **Solution A:** 11 µM *cyclized*-8; **Solution B:** 11 µM *cyclized*-8; 4 mM α–CD; [b] *Irradiation of solution A: 20s* λ = 365 nm (br.) (Supplementary Table **1**, entry **2**; ENB-280C/FE,).

PSS 365 nm ***cyclized*-8**

Supplementary Table 7 | Amounts of solutions **A** and **B** and concentrations obtained for the titration of irradiated ***cyclized*-8** for 60s λ = 365 nm (br.) (Supplementary Table **1**, entry **2**; ENB-280C/FE,).[a,b]

All solution were prepared in water (pH ≥ 9; room temperature). The concentration of ***cyclized*-8** was hold constant during the titration. Only solution A was irradiated.

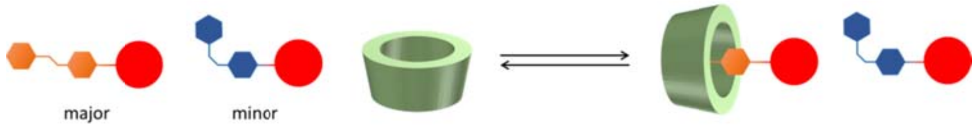

			[*cyclized*-**8**]	**α-CD**	
entry	**B** added (µL)	total amount (µL)	conc. (µM)	conc. (µM)	equiv. α-CD
1		2070	11	0	0
2	40	2110	11	75.8	6.89
3	80	2150	11	148.8	13.53
4	120	2190	11	219.2	19.93
5	160	2230	11	287.0	26.09
6	200	2270	11	352.4	32.04
7	240	2310	11	415.6	37.78
8	280	2350	11	476.6	43.33
9	320	2390	11	535.6	48.69

[a] **Solution A:** 11 µM *cyclized*-8; **Solution B:** 11 µM *cyclized*-8; 4 mM α–CD; [b] *Irradiation of solution A: 60s* λ = 365 nm (br.) (Supplementary Table **1**, entry **2**; ENB-280C/FE,).

Supplementary Methods

General Reagent Information: Preparation of commercially unavailable compounds: unless stated otherwise, all reactions were carried out in oven- and flame-dried glassware using standard Schlenk techniques and were run under nitrogen atmosphere. The reaction progress was monitored by TLC. Starting materials, reagents and solvents were purchased from *Sigma–Aldrich, Acros, Fluka, Fischer, Combi–Blocks, TCI, J.T. Baker or Macron* and were used as received, unless stated otherwise. Solvents for the reactions were of quality puriss., p.a.. Anhydrous solvents were purified by passage through solvent purification columns[3] (MBraun SPS-800). For aqueous solutions, deionized water was used.

Furfural, dimethylamine, ethylamine solution (2 M in THF), oxone (monopersulfate compound), anisidine, *N*,*N*-dimethylaniline, 1,4-dibromobutane, 1,12 dibromododecane and methyl 4-aminobenzoate were purchased from Sigma Aldrich. 1,3-Dimethylbarbituric acid and azobenzene were purchased from TCI Europe. 4-Aminoacetanilide was purchased from Acros Organics. 4-Methoxyazobenzene was purchased from Combi-Blocks.

General Considerations: Thin Layer Chromatography analyses were performed on commercial Kieselgel 60, F254 silica gel plates with fluorescence-indicator UV_{254} (*Merck*, TLC silica gel *60 F_{254}*). For detection of components, UV light at λ = 254 nm or λ = 365 nm was used. Alternatively, oxidative staining using aqueous basic potassium permanganate solution ($KMnO_4$) or aqueous acidic cerium phosphomolybdic acid solution (Seebach's stain[4]) was used. Drying of solutions was performed with $MgSO_4$ and volatiles were removed with a rotary evaporator. For lyophilization of compounds, samples were frozen in liquid N_2 and then freeze-dried with a LaboGene ScanVac CoolSafe 110-4 Pro Freeze Dryer at 168–163 K and 0.01 mbar for 16 h.

General Analytical Information: Nuclear Magnetic Resonance spectra were measured with an Agilent Technologies 400-MR (400/54 Premium Shielded) spectrometer (400 MHz). All spectra were measured at room temperature (22–24 °C). Chemical shifts for the specific NMR spectra were reported relative to the residual solvent peak [in ppm; $CDCl_3$: δ_H = 7.26; $CDCl_3$: δ_C = 77.16; CD_2Cl_2: δ_H = 5.32; CD_2Cl_2: δ_C = 53.84; d_6-DMSO: δ_H = 2.50; d_6-DMSO: δ_C = 39.52; toluene-d_8: δ_H = 2.08, 6.97, 7.01, 7.09; toluene-d_8: δ_C = 137.48, 128.87, 127.96, 125.13, 20.43; CD_3OD: δ_H = 3.31; CD_3OD: δ_C = 49.00][5]. The multiplicities of the signals are denoted by *s* (singlet), *d* (doublet), *t* (triplet), *q* (quartet), *m* (multiplet), *br* (broad signal), *app* (apparent). All ^{13}C-NMR spectra are ^{1}H-broadband decoupled.

High-resolution mass spectrometric measurements were performed using a Thermo scientific LTQ OrbitrapXL spectrometer with ESI ionization. The molecule-ion M^+, $[M + H]^+$ and $[M–X]^+$ respectively are given in *m/z*-units. Melting points were recorded using a Stuart analogue capillary melting point SMP11 apparatus. UV-vis absorption spectra were recorded on an Agilent 8453 UV-Visible Spectrophotometer in a 10 mm quartz cuvette using Uvasol® grade solvents. Half-lives of photoswitches were measured at λ_{max} and fitted with single exponential process. Intensities of light sources at λ = 400 nm and above were measured using a PM160 Power Meter with Photodiode Sensor (ThorLabs) at their corresponding peak-wavelength (λ_{max}). The distance from the light source is indicated.

Synthesis and Characterization

Synthesis of donor-acceptor Stenhouse adduct (DASA, 1):[6]

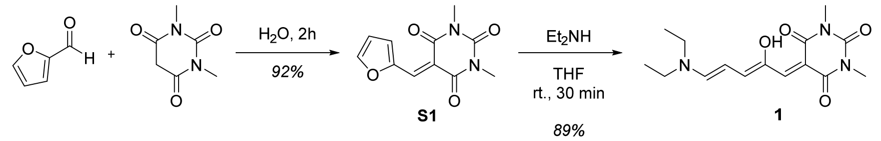

Compound **S1** has been prepared according to a reported procedure.[6a] Spectral properties matched previously reported values.

5-((2*Z*,4*E*)-5-(diethylamino)-2-hydroxypenta-2,4-dien-1-ylidene)-1,3-dimethylpyrimidine-2,4,6(1*H*,3*H*,5*H*)-trione (1):[4]

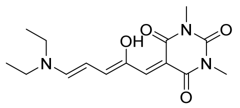

S1 (234 mg, 1.00 mmol) was suspended in tetrahydrofuran (10 mL). Subsequently, diethylamine (104 µL, 1.00 mmol) was added to the suspension at room temperature, followed by water (few drops). The reaction mixture was stirred for 30 min. at room temperature. The color of the reaction mixture turned dark purple.

Upon consumption of the starting material (TLC), additional tetrahydrofuran (10 mL) was added and the product was precipitated from the reaction mixture with cold pentane. Compound **1** was filtered to obtain dark purple crystals (275 mg, 89% yield) that can be further purified by recrystallization from warm acetonitrile if needed. **Mp.** 141-142 °C; **^{1}H NMR** (400 MHz, $CDCl_3$) δ 1.31 (t, *J* = 7.2 Hz, 3H, $NCH_2C\mathbf{H_3}$), 1.34 (t, *J* = 7.2 Hz, 3H, $NCH_2C\mathbf{H_3}$), 3.34 (s, 3H, $NC\mathbf{H_3}$), 3.35 (s, 3H, $NC\mathbf{H_3}$), 3.48 (q, *J* = 7.2 Hz, 2H, $NC\mathbf{H_2}CH_3$), 3.50 (q, *J* = 7.4 Hz, 2H, $NC\mathbf{H_2}CH_3$), 6.07 (t, *J* = 12.4 Hz, 1H, vinyl**H**), 6.75 (dd, *J* = 12.3, 1.5 Hz, 1H, vinyl**H**), 7.15 (s, 1H, vinyl**H**), 7.22 (d, *J* = 12.3 Hz, 1H, vinyl**H**), 12.54 (s, 1H, O**H**); **^{13}C NMR** (101 MHz, $CDCl_3$) δ 12.5, 14.7, 28.4, 28.6, 44.2, 51.9, 98.7, 102.7, 139.5, 146.7, 151.1, 152.1, 156.4, 163.5, 165.2; **HRMS** (ESI+) calc. for $C_{15}H_{22}N_3O_4$ $[M + H]^+$: 308.1608, found: 308.1605.

Synthesis of donor-acceptor Stenhouse adduct (DASA, 2):[6]

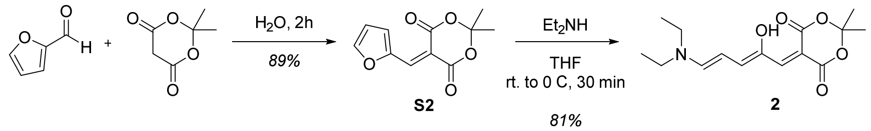

Compound **S2** has been prepared according to a reported procedure.[6a] Spectral properties matched previously reported values.

5-((2*Z*,4*E*)-5-(diethylamino)-2-hydroxypenta-2,4-dien-1-ylidene)-2,2-dimethyl-1,3-dioxane-4,6-dione (2):[4]

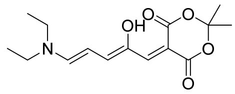

S2 (1.00 g, 4.50 mmol) was suspended in tetrahydrofuran (10 mL). Subsequently, diethylamine (465 µL, 4.50 mmol) was added to the suspension at room temperature, followed by water (few drops). The reaction mixture was stirred for 10 min. at room temperature and 20 min. at 0 °C. The color of the reaction mixture turned dark purple. Upon cooling, a precipitate was formed which was then collected by filtration and washed with cold diethyl ether and cold pentane and dried. The final product (**2**) was obtained as dark red crystals (1.08 g, 81% yield). **Mp.** 132 - 134 °C; **^{1}H NMR** (400 MHz, $CDCl_3$) δ 1.29 (t, *J* = 7.3 Hz, 3H, NCH_2C**H_3**), 1.33 (t, *J* = 7.2 Hz, 3H, NCH_2C**H_3**), 1.70 (s, 6H, C(C**H_3)$_2$**), 3.49 (q, *J* = 7.2 Hz, 4H, 2 x NC**H_2**CH_3), 6.05 (t, *J* = 12.3 Hz, 1H, vinyl**H**), 6.73 (dd, *J* = 12.4, 1.5 Hz, 1H, vinyl**H**), 7.28 (d, *J* = 12.3 Hz, 1H, vinyl**H**), 11.41 (s, 1H, O**H**); **^{13}C NMR** (101 MHz, $CDCl_3$) δ 12.4, 14.6, 26.8, 44.2, 52.0, 90.6, 102.4, 103.5, 139.0, 145.0, 151.5, 157.2, 165.4, 167.2; **HRMS** (ESI+) calc. for $C_{15}H_{22}NO_5$ $[M + H]^+$: 296.1493, found: 296.1496.

Synthesis of compound 3:[7–9]

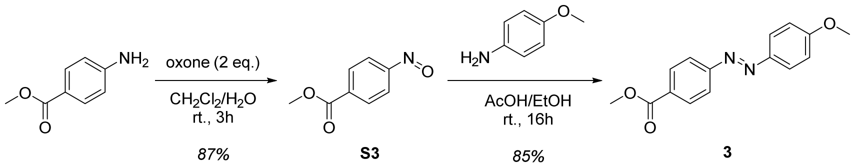

Compound **S3** has been prepared according to a reported procedure.[8] Spectral properties matched previously reported values.

Methyl (*E*)-4-((4-methoxyphenyl)diazenyl)benzoate (3):

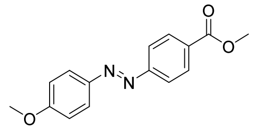

To a solution of methyl 4-nitrosobenzoate (**S3**, 1.00 g, 6.10 mmol) in EtOH (10 mL) was added 4-methoxyaniline (0.75 g, 6.10 mmol) and glacial acetic acid (10 mL). The reaction mixture was stirred at room temperature for 16 h.

After consumption of the substrates (TLC), water (20 mL) was added and the aqueous phase was extracted with CH_2Cl_2 (3 x 20 mL). The combined organic extracts were washed subsequently with 1M aq. HCl-solution (20 mL), sat. aq. $NaHCO_3$-solution (20 mL), water (20 mL) and sat. aq. NaCl-solution (20 mL) and were dried over $MgSO_4$, filtered and concentrated *in vacuo*. Recrystallization from hot EtOAc furnished compound **3** as bright orange crystals (1.39 g, 85% yield). **Mp.** 165-167 °C; **^{1}H NMR** (400 MHz, $CDCl_3$) δ 3.90 (s, 3H, OC**H₃**), 3.95 (s, 3H, CO_2C**H₃**), 7.03 (d, *J* = 8.9 Hz, 2H, Ar**H**), 7.91 (d, *J* = 8.5 Hz, 2H, Ar**H**), 7.95 (d, *J* = 9.0 Hz, 2H, Ar**H**), 8.17 (d, *J* = 8.5 Hz, 2H, Ar**H**); **^{13}C NMR** (101 MHz, $CDCl_3$) δ 52.4, 55.8, 114.5, 122.5, 125.3, 130.7, 131.3, 147.1, 155.5, 162.8, 166.8; **HRMS** (ESI+) calc. for $C_{15}H_{15}N_2O_3$ $[M + H]^+$: 271.1077, found: 271.1081. Spectral properties matched previously reported values.[9]

Characterization in toluene-*d₈*:

trans-3: ^{1}H NMR (400 MHz, toluene-d_8) δ 3.24 (s, 3H), 3.52 (s, 3H), 6.71 (d, *J* = 9.0 Hz, 2H), 7.90 (d, *J* = 8.8 Hz, 2H), 7.97 (d, *J* = 8.9 Hz, 2H), 8.14 (d, *J* = 8.8 Hz, 2H).

cis-3: ^{1}H NMR (400 MHz, toluene-d_8) δ 3.09 (s, 3H), 3.44 (s, 3H), 6.35 (d, *J* = 8.9 Hz, 2H), 6.56 (d, *J* = 8.4 Hz, 2H), 6.73 (d, *J* = 8.9 Hz, 2H), 7.82 (d, *J* = 8.5 Hz, 2H).

Methyl (*E*)-4-((4-(dimethylamino)phenyl)diazenyl)benzoate (4):

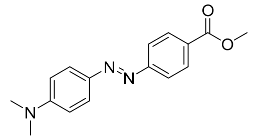

To a solution of methyl-4-aminobenzoate (300 mg, 2.00 mmol) in aq. 1M HCl (10 mL) was added drop-wise a solution of $NaNO_2$ (138 mg, 2.00 mmol) in H_2O (1.0 mL) on ice. This solution was stirred for 15 min at room temperature. The resulting mixture was slowly added to a solution of *N,N*-dimethylaniline (242 mg, 2.00 mmol) and NaOAc (57.0 mg, 0.70 mmol) in EtOH (10 mL), in an ice-water bath. Upon addition, the reaction mixture turned dark red and it was subsequently stirred for 1h in an ice-water bath and 1h at room temperature. CH_2Cl_2 (50 mL) was added to the reaction mixture and the layers were separated and the aqueous layer extracted with CH_2Cl_2 (3 x 50 mL). The combined organic extracts were washed with H_2O (50 mL), aq. 0.1 M HCl-solution (50 mL) and sat.

aq. NaCl-solution (2 x 50 mL) and dried over $MgSO_4$, filtered and concentrated *in vacuo.* Recrystallization from acetonitrile yielded **4** as bright red crystals. (250 mg, 45%). **Mp.** 183-184 °C; **^{1}H NMR** (400 MHz, $CDCl_3$) δ 3.11 (s, 6H, N(C**H$_3$**)$_2$), 3.94 (s, 3H, OC**H$_3$**), 6.76 (d, *J* = 9.2 Hz, 2H, Ar**H**), 7.87 (d, *J* = 8.5 Hz, 2H, Ar**H**), 7.91 (d, *J* = 9.1 Hz, 2H, Ar**H**), 8.14 (d, *J* = 8.6 Hz, 2H, Ar**H**); **^{13}C NMR** (101 MHz, $CDCl_3$) δ 40.4, 52.3, 111.7, 122.1, 125.7, 130.2, 130.7, 143.8, 153.0, 167.0; **HRMS** (ESI+) calc. for $C_{16}H_{18}N_3O_2$ $[M + H]^+$: 284.1394, found: 284.1395.

Characterization in toluene-*d$_8$*:
trans-4: ^{1}H NMR (400 MHz, toluene-*d$_8$*) δ 2.39 (s, 6H), 3.52 (s, 3H), 6.40 (d, *J* = 9.1 Hz, 2H), 7.98 (d, *J* = 8.5 Hz, 2H), 8.09 (d, *J* = 9.1 Hz, 2H), 8.17 (d, *J* = 8.5 Hz, 2H);
cis-4: ^{1}H NMR (400 MHz, toluene-*d$_8$*) δ 2.26 (s, 6H), 3.47 (s, 3H), 6.04 (d, *J* = 9.0 Hz, 2H), 6.71 (d, *J* = 8.3 Hz, 2H), 6.95 – 7.00 (m, 2H), 7.92 (d, *J* = 8.2 Hz, 2H).

(*E*)-azobenzene (5):

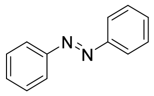

Characterization in toluene-*d$_8$*:
trans-5: ^{1}H NMR (400 MHz, toluene-*d$_8$*) δ 7.07 – 7.12 (m, 1H), 7.14 – 7.20 (m, 2H), 7.93 – 7.98 (m, 2H);
cis-5: ^{1}H NMR (400 MHz, toluene-*d$_8$*) δ 6.60 – 6.64 (m, 2H), 6.68 – 6.73 (m, 1H), 6.77 – 6.84 (m, 2H).

(*E*)- 4-Methoxyazobenzene (6):

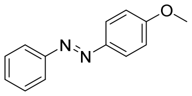

Characterization in toluene-*d$_8$*:
trans-6: ^{1}H NMR (400 MHz, toluene-*d$_8$*) δ 6.72 (d, *J* = 8.9 Hz, 2H), 7.10 – 7.13 (m, 1H), 7.21 (t, *J* = 7.7 Hz, 2H), 7.99 (d, *J* = 8.6 Hz, 4H).
cis-6: ^{1}H NMR (400 MHz, toluene-*d$_8$*) δ 6.37 (d, *J* = 8.9 Hz, 2H), 6.67 – 6.71 (m, 2H), 6.74 – 6.79 (m, 3H), 6.89 (t, *J* = 7.9 Hz, 2H).

Synthesis of compound 7:

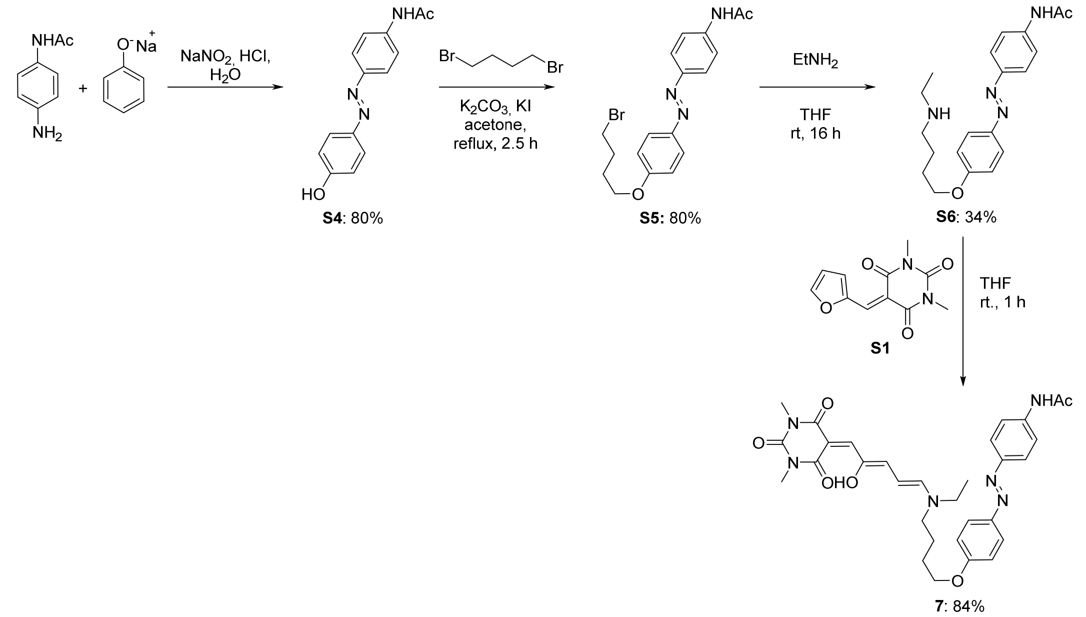

(*E*)-4-(4'-hydroxyphenylazo)acetanilide (S4, following a literature procedure**)**:[10]

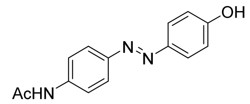

To an ice-cold solution of 4-amino-acetanilide (1.35 g, 9.00 mmol, 1.04 equiv.) in aq. 6N HCl (9.0 mL) was added drop-wise a solution of $NaNO_2$ (635 mg, 9.20 mmol, 1.02 equiv.) in water (2.0 mL), followed by a few crystals of urea. The resultant yellow mixture was added slowly to a cooled (ice-water bath) solution of phenol (828 mg, 8.80 mmol, 1.00 equiv.) and NaOH (1.70 g, 42.5 mmol, 4.70 equiv.) in water (6.0 mL). After 2h, the mixture was allowed to warm to room temperature and was acidified with 30% aq. HCl to pH = 6. The resultant precipitate was filtered off, washed with water and dried, furnishing product **S4** (1.79 g, 80%) as a brown solid. **^{1}H NMR** (400 MHz, DMSO-d_6): δ 2.07 (s, 3H, C**H$_3$**CO), 6.91 (d, ^3J = 8.0 Hz, 2H, Ar**H**), 7.71-7.90 (m, 6H, Ar**H**), 10.19 (s, 1H, N**H**). Spectral properties matched previously reported values.[8]

(*E*)-*N*-(4-((4-(4-bromobutoxy)phenyl)diazenyl)phenyl)acetamide (S5):

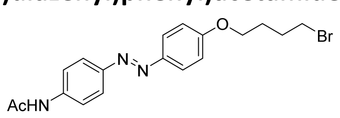

A solution containing (*E*)-4-(4'-hydroxyphenylazo)acetanilide (**S4**, 255 mg, 1.00 mmol), 1,4-dibromobutane (2.00 mL, 17.0 mmol, 17.0 equiv.), potassium carbonate (276 mg, 2.00 mmol, 2.00 equiv.) and potassium iodide (17 mg, 0.10 mmol, 0.10 equiv.) in acetone (10 mL) was heated under reflux for 2.5h. The reaction mixture was diluted with EtOAc (100 mL), washed with 1N aq. HCl (2 x 100 mL), sat aq. $NaHCO_3$ (100 mL) and sat. aq. NaCl solution (100 mL) and dried over $MgSO_4$. Solvents were partially evaporated, and the product was precipitated with pentane, to give **S5** (310 mg, 80% yield) as an orange solid. **Mp.** 172-174 °C; **^{1}H NMR** (400 MHz, DMSO-d_6): δ 1.75-1.96 (m, 4H, BrCH$_2$C**H$_2$**C**H$_2$**), 2.08 (s, 3H, C**H$_3$**CO), 3.60 (t, ^3J = 6.8 Hz, 2H, BrC**H$_2$**), 4.09 (t, ^3J = 6.4 Hz, 2H, OC**H$_2$**), 7.08 (d, ^3J = 8.0 Hz, 2H, Ar**H**), 7.70-7.85 (m, 6H, Ar**H**), 10.23 (s, 1H, N**H**); **^{13}C NMR** (101 MHz,

DMSO-d_6): δ 24.6, 27.8, 29.5, 35.2, 67.5, 115.4, 119.5, 123.7, 124.7, 142.2, 146.6, 147.9, 161.4, 169.1; **HRMS** (ESI+) calc. for $C_{18}H_{21}BrN_3O_2$: 390.0812, found: 390.0563.

(*E*)-*N*-(4-((4-(4-(ethylamino)butoxy)phenyl)diazenyl)phenyl)acetamide (S6):

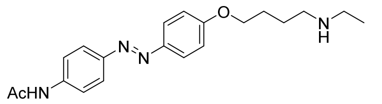

(*E*)-*N*-(4-((4-(4-bromobutoxy)phenyl)diazenyl)phenyl)acetamide (**S5**, 195 mg, 0.50 mmol) was added to ethylamine (2M in THF, 5.0 mL) and the resulting solution was stirred at room temperature for 16 h. The reaction mixture was added to 1N aq. HCl (50 mL) and the resulting mixture was washed with EtOAc (50 mL). The aqueous phase was alkalized with aq. KOH solution to pH > 10. The product was extracted with EtOAc (2 x 70 mL). The organic phase was dried ($MgSO_4$) and the solvent was evaporated. The product was recrystallized from hot Et_2O to furnish **S6** (60 mg, 34%) as an orange powder. **Mp.** 114-116 °C; **^{1}H NMR** (400 MHz, $CDCl_3$): δ 1.13 (t, ^3J = 7.2 Hz, 3H, C**H$_3$**CH$_2$), 1.60-1.90 (m, 5H, OCH$_2$C**H$_2$**C**H$_2$**, CH$_2$N**H**), 2.20 (s, 3H, C**H$_3$**CO), 2.65-2.75 (m, 4H, C**H$_2$**NHC**H$_2$**), 4.05 (t, ^3J = 6.4 Hz, 2H, OC**H$_2$**), 6.97 (d, ^3J = 8.4 Hz, 2H, Ar**H**), 7.58 (br s, 1H, AcN**H**), 7.64 (d, ^3J = 8.4 Hz, 2H, Ar**H**), 7.84-7.88 (m, 4H, Ar**H**); **^{13}C NMR** (101 MHz, DMSO-d_6): δ 15.6, 24.6, 26.4, 27.0, 43.9, 49.2, 68.4, 115.4, 119.5, 123.6, 124.7, 142.2, 146.6, 147.9, 161.5, 169.1; **HRMS** (ESI+) calc. for $C_{20}H_{27}N_4O_2$: 355.2129, found: 355.2126.

***N*-(4-((*E*)-(4-(4-((5-(1,3-dimethyl-2,4,6-trioxotetrahydropyrimidin-5(2*H*)-ylidene)-4-hydroxypenta-1,3-dien-1-yl)(ethyl)amino)butoxy)phenyl)diazenyl)phenyl)acetamide (7):**

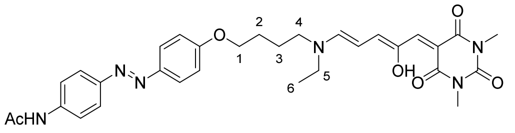

(*E*)-*N*-(4-((4-(4-(ethylamino)butoxy)phenyl)diazenyl)phenyl)acetamide (**S6**, 18 mg, 51 µmol) was suspended in tetrahydrofuran (0.5 mL). 5-(Furan-2-ylmethylene)-1,3-dimethylpyrimidine-2,4,6(1*H*,3*H*,5*H*)-trione (**S1**, 13 mg, 56 µmol, 1.10 equiv.) was subsequently added to the suspension at room temperature, followed by H_2O (three drops). The reaction mixture was stirred for 60 min at room temperature resulting in a dark purple solution. Upon consumption of the starting material (^{1}H NMR), the reaction mixture was diluted with tetrahydrofuran (3.0 mL) and addition of cold pentane (100 mL) to the solution resulted in deposition of product **7** on the walls of the flask. The product was washed subsequently with cold pentane (3 x 10 mL) and diethylether (3 x 10 mL) to furnish **7** (25.2 mg, 84% yield) as a mixture of protomers, which was observed before for donor-acceptor Stenhouse adducts.[4] **^{1}H NMR** (400 MHz, $CDCl_3$): δ 1.29 and 1.31 (two t, J = 7.2 Hz, 3H; NCH$_2$C**H$_3$**, *#6*), 1.73 – 1.94 (m, 4H; OCH$_2$C**H$_2$**C**H$_2$**, *#2 – #3*), 2.21 (s, 3H, C**H$_3$**CO), 3.36 (s, 6H, 2 x NC**H$_3$**), 3.45 (m, 4H; C**H$_2$**NC**H$_2$**, *#4, #6*), 4.03 (m, 2H; OC**H$_2$**, *#1*), 6.01 and 6.04 (two t, J = 12.3 Hz, 1H, vinyl**H**), 6.60 (dd, J = 22.4, 12.3 Hz, 1H, vinyl**H**), 6.90 – 6.99 (m, 2H, Ar**H**), 7.06 – 7.20 (m, 2H, vinyl**H**), 7.65 – 7.73 (m, 2H, Ar**H**), 7.84 – 7.90 (m, 4H, Ar**H**), 12.47 and 12.63 (two s, 1H, O**H**); **^{13}C NMR** (101 MHz, $CDCl_3$): δ 12.4, 14.7, 24.3, 24.9, 26.1, 26.2, 26.6, 28.4, 28.6, 44.3, 49.0, 52.2, 57.0, 67.4, 67.5, 98.8, 102.6, 102.8, 114.7, 114.8, 114.8, 115.3, 119.9, 123.7, 123.8, 124.7, 124.8, 139.6, 140.4, 140.5, 146.6, 146.7, 147.3, 147.3, 149.1, 149.1, 149.1, 150.9, 151.0, 152.0, 156.7, 156.9, 160.9, 161.0, 163.6, 163.6, 165.2, 168.7; **HRMS** (ESI+) calc. for $C_{31}H_{37}N_6O_6$: 589.2769, found: 589.2752.

Synthesis of compound 8:

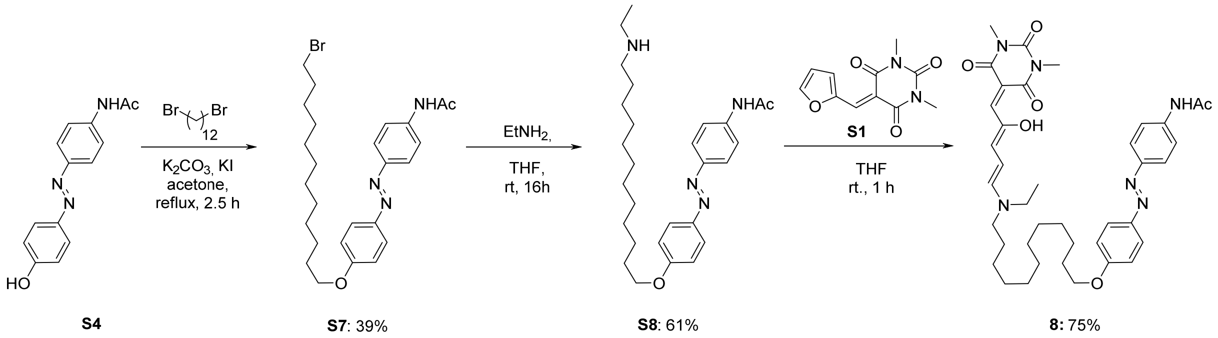

(*E*)-*N*-(4-((4-((12-bromododecyl)oxy)phenyl)diazenyl)phenyl)acetamide (S7):

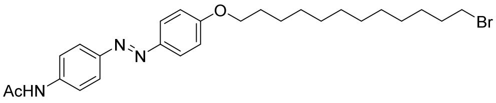

A solution containing (*E*)-4-(4'-hydroxyphenylazo)acetanilide (**S4**, 127 mg, 0.50 mmol), 1,12-dibromododecane (2.61 g, 8.00 mmol, 16.0 equiv.), potassium carbonate (138 mg, 1.00 mmol, 2.00 equiv.) and potassium iodide (8.3 mg, 0.05 mmol, 0.10 equiv.) in acetone (5.0 mL) was heated under reflux for 3 h. The reaction mixture was diluted with EtOAc (100 mL), washed with 1N aq. HCl solution (2 x 100 mL), sat aq. $NaHCO_3$ solution (100 mL) and sat. aq. NaCl solution (100 mL) and dried over $MgSO_4$. Volatiles were partially evaporated, and the product was precipitated with pentane, to give **S7** (97 mg, 39% yield) as a brownish solid. **Mp.** 120-121 °C; **^{1}H NMR** (400 MHz, DMSO-d_6): δ 1.20 – 1.46 (m, 16H, alkyl**H**), 1.68 – 1.82 (m, 4H, alkyl**H**), 2.09 (s, 3H, C**H$_3$**CO), 3.50 (t, *J* = 6.6 Hz, 3H, BrC**H$_2$**), 4.05 (t, *J* = 6.5 Hz, 2H, OC**H$_2$**), 7.09 (d, *J* = 8.6 Hz, 2H, Ar**H**), 7.75 – 7.86 (m, 6H, Ar**H**), 10.25 (s, 1H, AcN**H**); **^{13}C NMR** (101 MHz, DMSO-d_6): δ 24.1, 25.4, 27.5, 28.1, 28.6, 28.7, 28.9, 28.9, 28.9, 29.0, 32.2, 35.2, 67.9, 114.9, 119.1, 123.2, 124.2, 141.7, 146.1, 147.5, 161.1, 168.7; **HRMS** (ESI+) calc. for $C_{26}H_{37}BrN_3O_2$ $[M + H]^+$: 502.2064, found: 502.2049.

(*E*)-*N*-(4-((4-((12-(ethylamino)dodecyl)oxy)phenyl)diazenyl)phenyl)acetamide (S8):

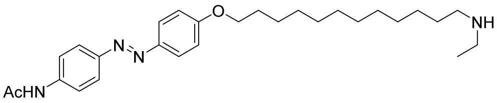

N-(4-((4-((12-bromododecyl)oxy)phenyl)diazenyl)phenyl)acetamide (**S7**, 60 mg, 119 µmol) was added to ethylamine (2M in THF, 4.0 mL) and the resulting solution was stirred at room temperature for 16 h. After completion of the reaction, sat. aq. $NaHCO_3$ solution (20 mL) was added to the reaction mixture, the phases were separated and the aqueous layer was extracted with EtOAc (3 x 20 mL). The combined organic layers were dried over $MgSO_4$, solvents were partially evaporated and the product was precipitated with pentane, to give **S8** (34 mg, 61% yield) as an orange solid. **Mp.** 109-111 °C; **^{1}H NMR** (400 MHz, $CDCl_3$) δ 0.97 (t, *J* = 7.1 Hz, 3H, NCH$_2$C**H$_3$**), 1.18 – 1.47 (m, 18H, alkyl**H**), 1.74 (m, 2H, alkyl**H**), 2.09 (s, 3H, C**H$_3$**CO), 2.44 (t, *J* = 7.0 Hz, 2H, NHC**H$_2$**); 2.48 (t, *J* = 7.6 Hz, 2H, NHC**H$_2$**CH$_3$); 4.06 (t, *J* = 6.5 Hz, 2H, OC**H$_2$**), 7.09 (d, *J* = 9.0 Hz, 2H, Ar**H**), 7.75 – 7.86 (m, 6H, Ar**H**), 10.25 (s, 1H, AcN**H**); **^{13}C NMR** (101 MHz, $CDCl_3$) δ 15.1, 24.1, 25.4, 26.9, 28.6, 28.7, 29.0, 29.0, 29.0, 29.0, 29.5, 29.5, 43.5, 49.2, 67.9, 115.0, 119.1, 123.2, 124.2, 141.7, 146.1, 147.5, 161.1, 168.7; **HRMS** (ESI+) calc. for $C_{28}H_{43}N_4O_2$ $[M + H]^+$: 467.3381, found: 467.3371.

***N*-(4-((*E*)-(4-((12-((5-(1,3-dimethyl-2,4,6-trioxotetrahydropyrimidin-5(2*H*)-ylidene)-4-hydroxypenta-1,3-dien-1-yl)(ethyl)amino)dodecyl)oxy)phenyl)diazenyl)phenyl) acetamide (8):**

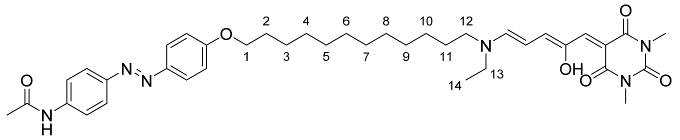

(*E*)-*N*-(4-((4-((12-(ethylamino)dodecyl)oxy)phenyl)diazenyl)phenyl)acetamide (**S8**, 19 mg, 41 µmol) was suspended in tetrahydrofuran (0.4 mL). 5-(Furan-2-ylmethylene)-1,3-dimethylpyrimidine-2,4,6(1*H*,3*H*,5*H*)-trione (**S1**, 10 mg, 45 µmol, 1.10 equiv.) was subsequently added to the suspension at room temperature, followed by H_2O (three drops). The reaction mixture was stirred for 60 min at room temperature resulting in a dark purple solution.

Upon consumption of the starting material (^{1}H NMR), the reaction mixture was diluted with tetrahydrofuran (3.0 mL) and addition of cold pentane (100 mL) to the solution resulted in precipitation of product **6**. The resulting dark purple crystals were collected by filtration and washed subsequently with cold pentane (3 x 10 mL) and diethylether (3 x 10 mL) to furnish **8** (21.4 mg, 75% yield) as a mixture of protomers. Protomers have been observed before with donor-acceptor Stenhouse adducts.[4] **Mp.** 111-112 °C; **^{1}H NMR** (400 MHz, $CDCl_3$) δ 1.17 – 1.40 (m, 17H; alkyl**H** *#4 – #10*; $NCH_2C\mathbf{H_3}$, *#14*), 1.41 – 1.52 (m, 2H; alkyl**H**, *#3*), 1.56 – 1.66 (m, 2H; alkyl**H**, *#11*), 1.76 – 1.86 (m, 2H; alkyl**H**, *#2*), 2.18 – 2.21 (m, 3H; $C\mathbf{H_3}CO$), 3.36 (s, 6H; 2 x $NC\mathbf{H_3}$), 3.25 – 3.47 (m, 4H; $C\mathbf{H_2}NC\mathbf{H_2}$, *#12 – #13*), 4.04 (t, *J* = 6.5 Hz, 2H; $OC\mathbf{H_2}$, *#1*), 6.02 (t, *J* = 12.3 Hz, 1H, vinyl**H**), 6.58 (t, *J* = 12.0 Hz, 1H, vinyl**H**), 6.98 (d, *J* = 9.0 Hz, 2H, Ar**H**), 7.08 (s, 1H, vinyl**H**), 7.10 (dd, *J* = 28.5, 12.3 Hz, 1H, vinyl**H**), 7.65 – 7.73 (m, 2H, Ar**H**), 7.83 – 7.89 (m, 4H, Ar**H**), 12.55 and 12.56 (two s, 1H, O**H**); **^{13}C NMR** (101 MHz, $CDCl_3$) δ 12.4, 14.6, 15.4, 24.8, 25.9, 26.0, 26.1, 26.1, 26.5, 27.0, 27.3, 28.4, 28.6, 29.1, 29.1, 29.1, 29.2, 29.3, 29.3, 29.3, 29.4, 29.4, 29.5, 29.5, 29.6, 29.7, 44.4, 49.5, 52.3, 57.4, 66.0, 68.3, 68.4, 68.5, 98.3, 102.9, 103.1, 114.8, 114.9, 119.9, 120.0, 123.7, 124.7, 138.7, 140.4, 146.6, 146.6, 147.0, 149.1, 151.5, 152.1, 157.1, 157.3, 161.6, 163.6, 163.7, 165.2, 168.7; **HRMS** (ESI+) calc. for $C_{39}H_{53}N_6O_6$ $[M + H]^+$: 701.4021, found: 701.4003.

5-((2*Z*,4*E*)-5-(dibutylamino)-2-hydroxypenta-2,4-dien-1-ylidene)-1,3-dimethylpyrimidine-2,4,6(1*H*,3*H*,5*H*)-trione (9):

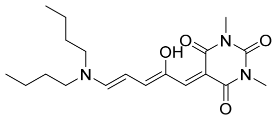

S1 (1.00 g, 4.27 mmol) was suspended in tetrahydrofuran (10 mL). Subsequently, di-*n*-butylamine (719 µL, 4.27 mmol) was added to the suspension at room temperature, followed by water (few drops). The reaction mixture was stirred for 30 min. at room temperature. The color of the reaction mixture turned dark purple. Upon consumption of the starting material (TLC), all volatiles were evaporated under reduced pressure. The dark purple residue was taken up in dichloromethane (20 mL) and 1M aq. HCl-solution (20 mL) was added. The phases were separated and the aqueous phase was extracted with CH_2Cl_2 (3 x 20 mL). The combined organic extracts were washed subsequently with 1M aq. HCl-solution (20 mL), sat. aq. $NaHCO_3$-solution (20 mL), water (20 mL) and sat. aq. NaCl-solution (20 mL) and were dried over $MgSO_4$, filtered and concentrated *in vacuo*. Compound **9** was obtained as dark purple solid (1.13 g, 73% yield). **^{1}H NMR** (400 MHz, $CDCl_3$) δ 0.96 (t, J = 7.4 Hz, 3H, $N(CH_2)_3C\mathbf{H_3}$), 0.97 (t, J = 7.3 Hz, 3H, $N(CH_2)_3C\mathbf{H_3}$), 1.35 (m, 4H, 2 x $NCH_2C\mathbf{H_2}CH_2CH_3$), 1.66 (m, 4H, 2 x $N(CH_2)_2C\mathbf{H_2}CH_3$), 3.33 (s, 3H, $NC\mathbf{H_3}$), 3.34 (s, 3H, $NC\mathbf{H_3}$), 3.36 – 3.43 (m, 4H, 2 x $NC\mathbf{H_2}(CH_2)_2CH_3$), 6.05 (t, J = 12.3 Hz, 1H, vinyl**H**), 6.73 (dd, J = 12.4, 1.4 Hz, 1H, vinyl**H**), 7.12 (s, 1H, vinyl**H**), 7.20 (d, J = 12.3 Hz, 1H, vinyl**H**), 12.53 (m, 1H, O**H**); **^{13}C NMR** (101 MHz, $CDCl_3$) δ 13.5, 13.6, 19.6, 20.1, 28.1, 28.2, 29.1, 30.9, 49.4, 57.4, 97.4, 103.2, 137.3, 146.2, 151.6, 151.8, 158.2, 163.1, 164.8; **HRMS** (ESI+) calc. for $C_{19}H_{30}N_3O_4$ $[M + H]^+$: 364.2231, found: 364.2234.

Determination of the Photochemical Quantum Yield[11]

The quantum yield (ϕ_{370}) for the photoswitching process of compound **3** at wavelengths λ = 370 nm (*trans-cis*; Supplementary Table **1**, entry **4**; MARL 260019 UV EMITTER, TO-46, 100DEG,) was determined in toluene (58.3 µM) at room temperature. The determined value is a result of triplicate measurements. The switching process was followed by UV-vis spectroscopy and the change in absorbance determined at λ_{max} = 360 nm. Care was taken that the toluene solution containing **3** absorbs ≥ 95% of the incident light (Absorbance ≥ 1.98). Furthermore, short irradiation times were used to keep conversions low (~10%, linear regime of Δt and ΔA). The incident light intensity was determined using standard ferrioxalate actinometry[11a] under identical irradiation conditions (Reference value: $\phi_{act,365.6}$ = 1.21[11b]). The quantum yield was determined as ϕ = 0.15.

Binding Studies

Phase-Transfer

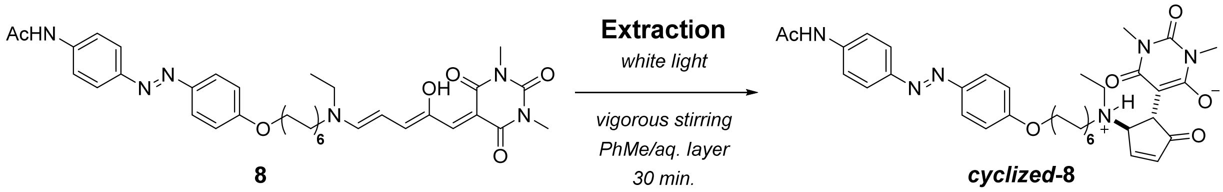

In a 4 mL vial with cap, compound **8** (1–2 mg) was dissolved in toluene (1.0 mL). Basic aqueous K_2CO_3-solution (pH ≥ 9) was added (1.0 mL). The biphasic mixture was stirred vigorously for 30 min. under white light (halogen) irradiation (Supplementary Table **1**, entry **13**; Plusline ES Small 160W 3100 lm,). Phase transfer of compound **8** from the organic layer to the aqueous layer is apparent through color change. No remaining DASA was detected by ^{1}H-NMR spectroscopy. The organic layer was lyophilized overnight to obtain a mixture of ***cyclized*-8** and K_2CO_3.

UV-vis Studies on Host-Guest Binding[1,2]

UV-vis titrations were performed to assess the binding of the azobenzene moiety of ***cyclized*-8** to α-CD. To a solution **A** (11 µM ***cyclized*-8**), a solution **B** (11 µM ***cyclized*-8**; 4 mM α–CD) was added stepwise and the change in the absorption spectrum quantified.

The following titrations were performed:

Binding:

- Thermally adapted ***cyclized*-8** (Supplementary Table 3, Supplementary Figure 63)
- PSS 365 nm ***cyclized*-8** (Supplementary Table 4, Supplementary Figure 64)

Controls:

- *Control experiment 1* (Supplementary Table 5, Supplementary Figure 65)
  Rationale: An intermediate ratio of ***cis/trans-cyclized*-8** would be expected to show an intermediate change of the absorption spectra upon binding.
- *Control experiment 2*
  Rationale: Irradiation of solution **A**, but not **B** should result in a more pronounced change of the absorption spectra upon binding during titration as the concentration of ***cyclized*-8** remains unaltered, but the ratio of *cis/trans* changes.
  - 20s 365 nm ***cyclized*-8** (Supplementary Table 6, Supplementary Figure 66)
  - PSS 365 nm ***cyclized*-8** (Supplementary Table 7, Supplementary Figure 67)

Supplementary References

1 Rekharsky, M. V. & Inoue, Y. Complexation Thermodynamics of Cyclodextrins. *Chem. Rev.* **98,** 1875–1918 (1998).

2 a) Matsui, Y. & Mochida, K. *Bull. Chem. Soc. Jpn.* Binding forces contributing to the association of cyclodextrin with alcohol in an aqueous solution. **52,** 2808–2814 (1979); b) Lin, S.-F. & Connors, K. A. Complex formation between α-cyclodextrin and 4-substituted phenols studied by potentiometric and competitive spectrophotometric methods. *J. Pharm. Sci.* **72,** 1333–1338 (1983); c) Ebel, S.& Karger, A. Precision of parameters determined by spectrophotometric measurements: Part 1. Precision of the 1:1 β-cyclodextrin ligand-binding constant as obtained by spectrophotometric determination. *Chemom. Intell. Lab. Syst.* **6,** 301–311 (1989); d) Darrington, R. T.; Xiang, T.-X. & Anderson, B. D. Inclusion complexes of purine nucleosides with cyclodextrins: I. Complexation and stabilization of a dideoxypurine nucleoside with 2-hydroxypropyl-β-cyclodextrin. *Int. J. Pharm.* **59,** 35–44 (1990); e) Kitamura, K. & Imayoshi, N. Second-derivative spectrophotometric determination of the binding constant between chlorpromazine and -cyclodextrin in aqueous solutions. *Anal. Sci.* **8,** 497–501 (1992).

3 Pangborn, A. B., Giardello, M. A., Grubbs, R. H., Rosen, R. K. & Timmers, F. J. Safe and convenient procedure for solvent purification. *Organometallics* **15,** 1518–1520 (1996).

4 Seebach, D., Imwinkelried, R. & Stucky, G. Optically active alcohols from 1,3-Dioxan-4-ones. A practical version of enantioselective synthesis with nucleophilic substitution at acetal centers. *Helv. Chim. Acta* **70,** 448–464 (1987).

5 Fulmer, G. R. *et al.* NMR chemical shifts of trace impurities: Common laboratory solvents, organics, and gases in deuterated solvents relevant to the organometallic chemist. *Organometallics* **29,** 2176–2179 (2010).

6 a) Helmy, S. *et al.* Photoswitching using visible light: A new class of organic photochromic molecules. *J. Am. Chem. Soc.* **136,** 8169–8172 (2014); b) Helmy, S., Oh, S., Leibfarth, F. A., Hawker, C. J. & Read de Alaniz, J. Design and synthesis of donor-acceptor Stenhouse adducts: A visible light photoswitch derived from furfural. *J. Org. Chem.* **79,** 11316–11329 (2014).

7 Hansen, M. J. *et al.* Proteasome inhibitors with photocontrolled activity. *ChemBioChem* **15,** 2053–2057 (2014).

8 Priewisch, B. & Rück-Braun, K. Efficient preparation of nitrosoarenes for the synthesis of azobenzenes. *J. Org. Chem.* **70,** 2350–2352 (2005).

9 Davey, M.H.; Lee, V.Y.; Miller, R.D. & Marks, T.J. Synthesis of aryl nitroso derivatives by tert-butyl hypochlorite oxidation in homogeneous media. Intermediates for the preparation of high-hyperpolarizability chromophore skeletons. *J. Org. Chem.* **64,** 4976–4979 (1999)

10 Cisnetti, F. *et. al.* Photochemical and electronic properties of conjugated bis(azo) compounds: An experimental and computational study. *Chem. Eur. J.* **10,** 2011–2021 (2004).

11 a) Hatchard, C. G. & Parker, C. A. A new sensitive chemical actinometer. II. Potassium ferrioxalate as a standard chemical actinometer. *Proc. R. Soc. London Ser. A Math. Phys. Eng. Sci.* **235,** 518–536 (1956); b) Montalti, M., Credi, A, Prodi, L. & Gandolfi, M.T. *Handbook of Photochemistry*, Third Edition, (CRC Press, 2006).
